# The oldest ceratosaurian (Dinosauria: Theropoda), from the Lower Jurassic of Italy, sheds light on the evolution of the three-fingered hand of birds

**DOI:** 10.7717/peerj.5976

**Published:** 2018-12-19

**Authors:** Cristiano Dal Sasso, Simone Maganuco, Andrea Cau

**Affiliations:** 1 Museo di Storia Naturale di Milano, Milano, Italy; 2 Museo Geologico “Giovanni Capellini”, Bologna, Italy

**Keywords:** Dinosauria, Theropoda, Ceratosauria, Taphonomy, Osteology, Phylogeny, Hand evolution, Aves, Lower Jurassic, Italy

## Abstract

The homology of the tridactyl hand of birds is a still debated subject, with both paleontological and developmental evidence used in support of alternative identity patterns in the avian fingers. With its simplified phalangeal morphology, the Late Jurassic ceratosaurian *Limusaurus* has been argued to support a II–III–IV digital identity in birds and a complex pattern of homeotic transformations in three-fingered (tetanuran) theropods. We report a new large-bodied theropod, *Saltriovenator zanellai* gen. et sp. nov., based on a partial skeleton from the marine Saltrio Formation (Sinemurian, lowermost Jurassic) of Lombardy (Northern Italy). Taphonomical analyses show bone bioerosion by marine invertebrates (first record for dinosaurian remains) and suggest a complex history for the carcass before being deposited on a well-oxygenated and well-illuminated sea bottom. *Saltriovenator* shows a mosaic of features seen in four-fingered theropods and in basal tetanurans. Phylogenetic analysis supports sister taxon relationships between the new Italian theropod and the younger Early Jurassic *Berberosaurus* from Morocco, in a lineage which is the basalmost of Ceratosauria. Compared to the atrophied hand of later members of Ceratosauria, *Saltriovenator* demonstrates that a fully functional hand, well-adapted for struggling and grasping, was primitively present in ceratosaurians. Ancestral state reconstruction along the avian stem supports 2-3-4-1-X and 2-3-4-0-X as the manual phalangeal formulae at the roots of Ceratosauria and Tetanurae, confirming the I–II–III pattern in the homology of the avian fingers. Accordingly, the peculiar hand of *Limusaurus* represents a derived condition restricted to late-diverging ceratosaurians and cannot help in elucidating the origin of the three-fingered condition of tetanurans. The evolution of the tridactyl hand of birds is explained by step-wise lateral simplification among non-tetanuran theropod dinosaurs, followed by a single primary axis shift from digit position 4 to 3 at the root of Tetanurae once the fourth finger was completely lost, which allowed independent losses of the vestigial fourth metacarpal among allosaurians, tyrannosauroids, and maniraptoromorphs. With an estimated body length of 7.5 m, *Saltriovenator* is the largest and most robust theropod from the Early Jurassic, pre-dating the occurrence in theropods of a body mass approaching 1,000 Kg by over 25 My. The radiation of larger and relatively stockier averostran theropods earlier than previously known may represent one of the factors that ignited the trend toward gigantism in Early Jurassic sauropods.

## Introduction

Although most of the skeletal features differentiating birds from other extant vertebrates can be tracked back to the Mesozoic dinosaurs (Makovicky & Zanno, 2011; Xu et al., 2014a), the integration of the fossil record of stem-avians (all taxa closer to birds than crocodiles) with the developmental biology of living birds is more controversial. The evolution of the three-fingered hand of birds from the ancestral pentadactyl condition of tetrapods is still debated, the former having been considered alternatively as homologous to the medialmost three (I–II–III) or the central (II–III–IV) fingers of reptiles (Wagner & Gauthier, 1999; Bever, Gauthier & Wagner, 2011; Xu et al., 2014a). This controversy has often been depicted as a dichotomy between a paleontological approach supporting the I–II–III pattern in three-fingered theropods (tetanurans), and a developmental approach supporting the II–III–IV pattern based on the topology of the embryonic mesenchymal condensations from which the avian digits develop (Wagner & Gauthier, 1999). Yet, both fossil and embriological data are involved in the two alternative interpretations (Bever, Gauthier & Wagner, 2011; Vargas et al., 2008; Xu et al., 2009; Tamura et al., 2011), and may eventually support additional, more complex, homology frameworks (Xu et al., 2014a). Pivotal among the fossil evidence, the unusual hand of the Late Jurassic ceratosaurian *Limusaurus* has been argued to support a II–III–IV digital identity in birds and a complex pattern of homeotic transformations in three-fingered (tetanuran) theropods (Xu et al., 2009; Bever, Gauthier & Wagner, 2011), although criticism to this interpretation has been raised from both paleontological and developmental perspectives (Wang et al., 2011; Carrano & Choiniere, 2016). Following the reinterpretation of the digital identity along the avian stem of Xu et al. (2009), a series of paleontological studies in the last decade used the II–III–IV homology pattern as morphological framework for three-fingered theropods, challenging the I–II–III pattern traditionally followed in the interpretation of the theropod hand (Xu, Han & Zhao, 2014b). It must be remarked that the evolutionary scenario supporting the II–III–IV homology pattern of Xu et al. (2009) makes predictions that can be falsified in the fossil record (Bever, Gauthier & Wagner, 2011): the phalangeal formula at the root of Ceratosauria should be markedly simplified, compared to the ancestral theropod formula (i.e., 0-3-3/2-1-X vs 2-3-4-1-0).

Here, we report a new ceratosaurian theropod, *Saltriovenator zanellai*, from the Saltrio Formation (Lower Jurassic, lower Sinemurian, ∼198 Mya) of Northern Italy (Dal Sasso, 2003), which shows a mosaic of features seen in four-fingered theropods and in basal tetanurans. Although fragmentary, the new theropod allows to reconstruct the ancestral condition for ceratosaurian hand, shedding light on the evolutionary digit pattern in tetanuran fingers and thus along the lineage leading to bird origin. The occurrence of large averostran theropods in the fossil record is also analyzed in the light of the reconstructed body size of the new Italian specimen and its stratigraphic and geochronological context.

### The new find, in the context of Early Jurassic neotheropods

Skeletal remains of theropod dinosaurs are extremely rare in the Lower Jurassic and most reports are of only fragmentary remains (Benton, Martill & Taylor, 1995; Owen, 1863; Woodward, 1908; Andrews, 1921; Cuny & Galton, 1993; Delsate & Ezcurra, 2014). Moreover, ceratosaurian-grade taxa are absent until Middle Jurassic times (Maganuco et al., 2007; Pol & Rauhut, 2012), with one exception from the Pliensbachian–Toarcian of Northern Africa (Allain et al., 2007). This paucity of skeletal remains results in a considerable gap in our knowledge of these animals at a time when theropods were diversifying rapidly in the aftermath of the Triassic–Jurassic mass extinction event, as it is proven by the rich and worldwide distributed ichnofossil record (Delsate & Ezcurra, 2014, and references therein).

In Europe, theropod remains are reported from Hettangian times and are mostly non-diagnostic at generic level: Scotland (Benton, Martill & Taylor, 1995), England (Owen, 1863; Woodward, 1908; Andrews, 1921), France (Cuny & Galton, 1993), and Luxembourg (Delsate & Ezcurra, 2014). Two species of the genus *Sarcosaurus* have been reported from the Hettangian of England, *S. woodi* from Barrow upon Soar, Leicestershire, based on an isolated pelvis, vertebra, and proximal femur (BMNH 4840/1), and *S. andrewsi* (Huene, 1932), based on a partial tibia (NHMUK R3542) (see also Woodward, 1908). The neotheropod *Dracoraptor hanigani*, from the Hettangian of Wales, has been recently described by Martill et al. (2016) on the basis of a 40% complete skeleton including cranial and postcranial material.

In the rest of the world, the most famous Early Jurassic theropod is certainly *Dilophosaurus wetherilli* from the Hettangian of Arizona (Welles, 1954, 1984), which is known from several specimens. Other relevant taxa are *Sinosaurus (=“Dilophosaurus” sinensis)* from the Hettangian–Sinemurian of China (Hu, 1993), *Coelophysis rhodesiensis* from the Hettangian–Pliensbachian of South Africa and Zimbabwe (Raath, 1990), *Dracovenator* from the Hettangian of South Africa (Yates, 2005), *Cryolophosaurus* from the Early Jurassic (?Sinemurian–Pliensbachian) of Antarctica (Hammer & Hickerson, 1994), *Podokesaurus* from the Pliensbachian to Toarcian of Massachussetts (Talbot, 1911), *Segisaurus* from the Pliensbachian to Toarcian of Arizona (Carrano, Hutchinson & Sampson, 2005), “*Syntarsus*” *kayentakatae* from the Hettangian of Arizona (Rowe, 1989), and *Berberosaurus* from the Toarcian of Morocco (Allain et al., 2007). We do not take into consideration the enigmatic genus *Eshanosaurus* from the Lower Jurassic of China, tentatively dated as Hettangian (Xu, Zhao & Clark, 2001), pending correct identification and reliably dating, as this purported therizinosaurian coelurosaur might be a sauropodomorph as well.

In this context, the discovery of a new specimen from the Sinemurian of Italy is extremely relevant as it is among the oldest Jurassic theropods, it is larger than all other pre-Aalenian theropods (see Skeletal reconstruction and body size section, below) and it improves our knowledge on some of the macroevolutionary patterns that would have characterized the evolution of Theropoda during the Jurassic. It also represents the first dinosaur skeleton from the Italian Alps, the first of Jurassic age, and the second theropod skeleton found in Italy after *Scipionyx samniticus* (Dal Sasso & Signore, 1998; Dal Sasso & Maganuco, 2011).

The discovery of the specimen here described was accidental (for a more detailed account, see Dal Sasso, 2004). In the summer of 1996, Angelo Zanella, fossil amateur and collaborator of the Museo di Storia Naturale di Milano (MSNM), spotted some bones emerging from large blocks of rock in a huge quarry located in the Alpine foothills, at the Swiss–Italian border near Saltrio, less than 80 km north of Milan (Varese Province, Lombardy). Mr. Zanella reported the bones to the MSNM, which arranged a rapid prospection and recovered more remains. The research was difficult because the explosives used for industrial quarrying had blown up the fossil-bearing layer and had broken it into hundreds of pieces. In fact, the Saltrio quarry is active since the 15th century as one of the finest sites of marble production, and the “Saltrio Stone” provided high quality matter during the building of famous Italian monuments, such as the Scala Theatre in Milan, and the Mole Antonelliana in Turin.

In 1999, after 1,800 h of chemical preparation in the Laboratory of the MSNM, 132 remains were extracted from three main blocks. Although fragmentary, jaw fragments, one tooth, rib remains, pectoral and limb bones were resulted to be part of a large theropod dinosaur. The Saltrio theropod (MSNM V3664) became popular by the name “saltriosauro” and so it was reported (Dal Sasso, 2001a) and preliminarily described (Dal Sasso, 2001b, 2004). Actually, even though sometimes latinized (Dalla Vecchia, 2001), any pseudo-scientific name given to the specimen in the past is a *nomen nudum*, not valid because its erection did not follow the International Commission on Zoological Nomenclature (ICZN) rules (i.e., no diagnosis, neither accession number were provided in the publication erecting that name): that is one of the aims of the present contribution.

## Materials and Methods

### Fossil preparation

Removal of the fossil bones from the hard dolomitic matrix took more than 1 year (Dal Sasso, 2001b, 2004; Dal Sasso, Magnoni & Fogliazza, 2001). The methods used were a combination of mechanical preparation and controlled chemical preparation. Once the largest portions of matrix devoid of bones were cut away, the fossiliferous blocks were repeatedly immersed in a water solution of formic acid (5%) previously saturated with calcium triphosphate, then washed under abundant water current, then dried up, and the gradually surfacing bone was protected with an ethyl methacrylate co-polymer (Paraloid B72). This cycle involved about half a ton of limestone and took about 1,800 h.

### Material

A total number of 132 bone pieces were recovered in close association, all clearly belonging to a single individual (except for one tooth and one jaw fragment, pertaining to a bony fish). The material consists of: 35 determinable bones, representing the holotypic material and belonging to the right lower jaw, pectoral girdle, rib cage and forelimbs, right manus, right ankle, and metapodium; 29 partially determinable bone pieces (five cranio-mandibular fragments, four rib fragments; five coracoidal, five scapular, and three sternal fragments; four appendicular skeletal fragments, including three possibly ungual fragments); 68 totally indeterminate bone pieces, including 16 small fragments surfaced in situ and 52 very small fragments recovered during preparation.

### Methods

Measurements of the bones were taken with a digital caliper and a goniometer. In the present paper, if not differently specified, length of a given fragmentary element indicates its maximum length, and its height or width or diameter were taken perpendicular to the maximum length.

Thin sections of the embedding sediment were made, in order to observe microfossils and study the sedimentology and the depositional environment; microfossils were also collected by sieving the residual fraction of the acid preparation process.

Two bone samples were obtained from selected skeletal elements, for paleohistological analysis. The samples were mounted on glass slides, polished down to obtain thin sections with a thickness of ∼50 μm, and analyzed under a Nikon Eclipse E600 POL mineralogical microscope. Photographs were taken with the gypsum plate inserted. Definition and terminology of lines of arrested growth (LAGs), external fundamental system (EFS), and vascular categorization follow Chinsamy (2005), Erickson (2005) and Francillon-Vieillot et al. (1990).

X-ray computed tomography (CT) of selected appendicular elements was performed at the Radiology Department of the Fondazione Ospedale Maggiore di Milano, with a Siemens Somatom Definition Dual Source CT Scanner. The best CT imaging was obtained with a bone algorithm on transverse (axial) slices, with scan parameters 120 kV, 120 mA, and slice thickness of 0.3 mm. Data was exported in DICOM format using eFilm (v. 1.5.3; Merge eFilm, Toronto, Canada). Analysis and post-processing were performed at Siemens Milano, Italy, with SyngoVia post-processing system using Region Growing Algorithm to segment volumes and see internal anatomical structures and vacuities.

We used photogrammetry to better show and study the mobility of the manus. 3D models of the bones were obtained with Agisoft PhotoScan, by processing 60 shots for each bone element. The photos were taken with a Nikon D90 camera, using a light box. The models were animated and rendered with Maxon Cinema 4D.

For the anatomical nomenclature, following Weishampel, Dodson & Osmólska (2004) we adopted the terminology of the *Nomina Anatomica Veterinaria* (World Association of Veterinary Anatomist (WAVA), 2005) and the *Nomina Anatomica Avium* (Baumel et al., 1993). Concerning the dental nomenclature, we followed the standardization established by Hendrickx, Mateus & Araújo (2015a).

### Phylogenetic taxonomy

In this study, we adopted the following clade name definitions. **Dinosauria**: the least inclusive clade containing *Megalosaurus bucklandii*, *Hylaeosaurus armatus*, *Plateosaurus engelhardti*, and *Iguanodon bernissartensis* (emended). **Saurischia**: the most inclusive clade containing *Allosaurus fragilis* and *Diplodocus longus* but not *I. bernissartensis*. **Theropoda**: the most inclusive clade containing *Allosaurus fragilis* but not *Plateosaurus engelhardti* or *Heterodontosaurus tuckii* (Naish et al., in press). **Neotheropoda**: the least inclusive clade containing *Allosaurus fragilis*, *Ceratosaurus nasicornis* and *Coelophysis bauri* (emended). **Coelophysoidea**: the most inclusive clade containing *Coelophysis bauri* but not *Allosaurus fragilis* or *Ceratosaurus nasicornis*. **Dilophosauridae**: the most inclusive clade containing *Dilophosaurus wetherilli* but not *Allosaurus fragilis*, *Coelophysis bauri*, or *Ceratosaurus nasicornis* (new definition). **Averostra**: the least inclusive clade containing *Vultur gryphus* and *Ceratosaurus nasicornis* but not *Coelophysis bauri* (emended). **Tetanurae**: the most inclusive clade containing *Vultur gryphus* but not *Ceratosaurus nasicornis* (emended). **Ceratosauria**: the most inclusive clade containing *Ceratosaurus nasicornis* but not *Vultur gryphus* (emended). **Neoceratosauria**: the least inclusive clade containing *Ceratosaurus nasicornis* and *Abelisaurus comahuensis* (emended). **Ceratosauridae**: the most inclusive clade containing *Ceratosaurus nasicornis* but not *Abelisaurus comahuensis* or *Noasaurus leali*. **Abelisauroidea**: the least inclusive clade containing *Abelisaurus comahuensis* and *Noasaurus leali*.

Following Bristowe & Raath (2004), the binomial “*Syntarsus rhodesiensis*” is considered a junior synonym of *Coelophysis rhodesiensis*. The binomial “*Syntarsus kayentakatae*” is provisionally used for the Kayenta Formation coelophysid (Rowe, 1989), pending the formal definition of a genus name for the latter (see Bristowe & Raath, 2004; Tykoski & Rowe, 2004).

### Phylogenetic analysis

The phylogenetic data set used for investigating the affinities of the new Italian theropod includes 87 operational taxonomic units scored for 1,781 morphological character statements (Data S1). Character statement definitions are based on Cau (2018). The data set was analyzed using maximum parsimony as tree search strategy. Parsimony analyses were performed using TNT version 1.5 (Goloboff, Farris & Nixon, 2008). Given the large size of the data set, the search strategy involved 100 “New Technology” search analyses using the default setting, followed by a series of “New Technology” search analyses exploring the tree islands found during the first round. Then, the analysis explored the tree islands recovered during the “New Technology” analysis rounds, using “Traditional Search” analysis and saving up to 99.999 shortest trees (default maximum storage in TNT). Nodal support was calculated saving all trees up to 10 steps longer than the shortest topologies found and using the “Bremer Supports” function of TNT.

### Nomenclatural acts

The electronic version of this article in portable document format will represent a published work according to the ICZN, and hence the new names contained in the electronic version are effectively published under that Code from the electronic edition alone. This published work and the nomenclatural acts it contains have been registered in ZooBank, the online registration system for the ICZN. The ZooBank LSIDs (Life Science Identifiers) can be resolved and the associated information viewed through any standard web browser by appending the LSID to the prefix http://zoobank.org/. The LSID for this publication is:

LSID urn:lsid:zoobank.org:pub:DBF732EB-6D24-48D2-294 8E5E-1C83EB380FD2.

The online version of this work is archived and available from the following digital repositories: PeerJ, PubMed Central, and CLOCKSS.

## Geological Setting

The recovery of terrestrial vertebrates in the marine Jurassic beds of Europe is not rare, to the point that most of the fragmentary theropod remains from the Hettangian of Europe have been obtained from marine or marginal marine strata (Martill et al., 2016). This situation was probably favored by peculiar and similar paleogeographic conditions (see also Benton, Martill & Taylor, 1995), which are not much different from our case. Specimen MSNM V3664 comes from the Saltrio Fm. (*sensu* Gnaccolini, 1964), a limestone very rich in marine macro- and microfossils, which deposited at the bottom of an open sea basin during Early Jurassic (Sinemurian) times (Wiedenmayer, 1963; Sacchi Vialli, 1964; Gaetani, 1975; Kalin & Trumpy, 1977).

**Figure 1:**
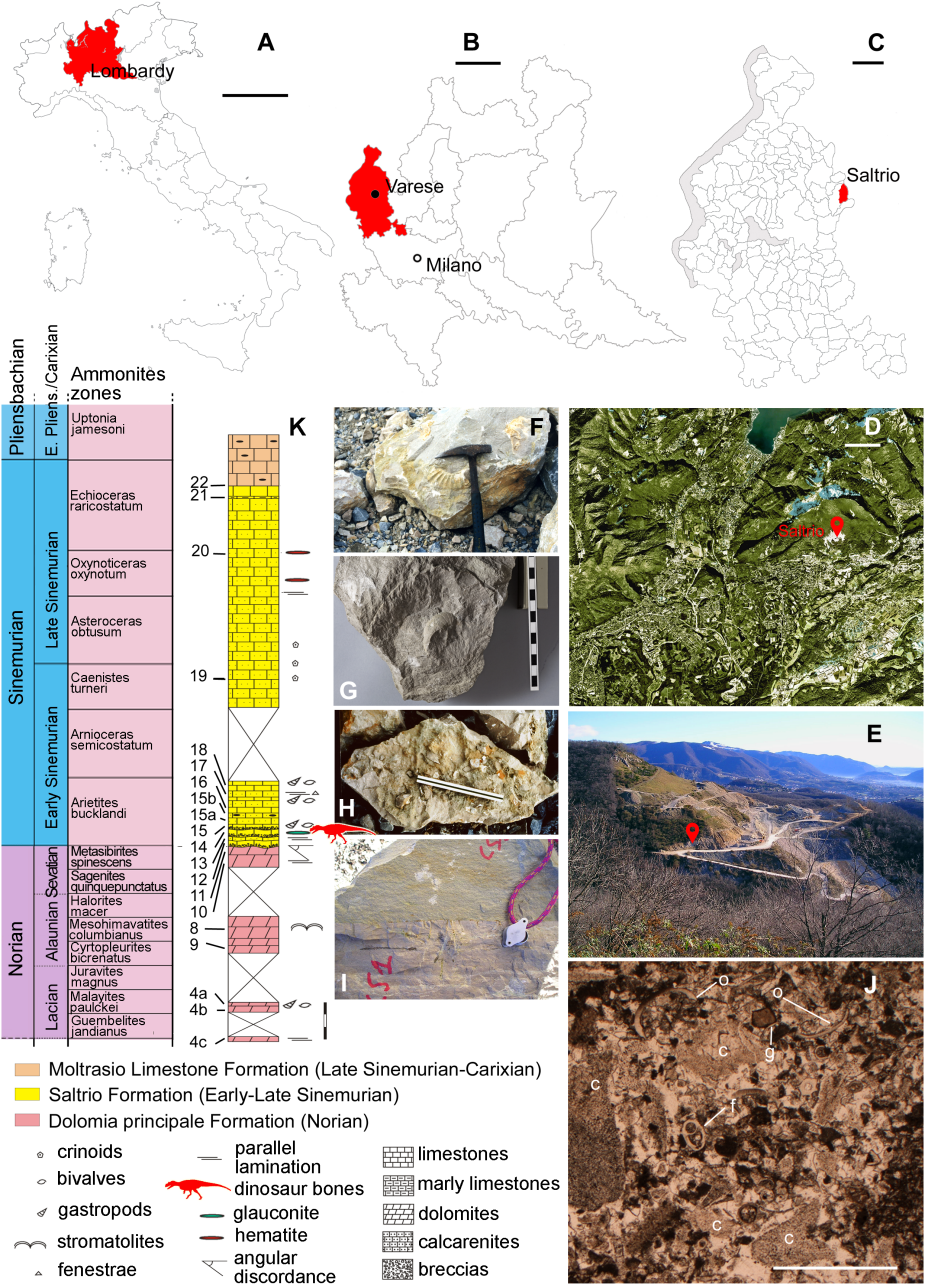
Fossil location and geological setting. (A–C) Outline maps of Italy, Lombardy, Varese Province, and Saltrio Municipality; (D) satellite view of the Saltrio area, with map marker indicating the Saltrio quarry; (E) map marker indicating the stratigraphic log in the Saltrio quarry; (F) the ammonite *Paracoroniceras* cf. *gmuendense* and (G) the nautiloid *Cenoceras striatum*, both found associated in the layer containing the dinosaur bones; (H) glauconite present as accessory mineral in block C (counterpart of block A of Fig. 2); (I) the discordance between the Dolomia Principale Fm. and the Saltrio Fm.; (J) thin sections of the layer embedding the dinosaur bones; (K) stratigraphic log of the Saltrio quarry, based on Croce (2005), with geological time scale and ammonites zones based on Sacchi Vialli (1964) and Ogg & Hinnov (2012). Abbreviations: c, crinoids; f, foraminifers; g, gastropods; o, ostracods. Scale bars equal 200 km in (A), 30 km in (B), six km in (C), one km in (D), one mm in (K), and 150 cm in (L). Photos by F. Berra, G. Bindellini, M. Croce, and G. Pasini; drawings by M. Croce and S. Maganuco.

The Saltrio Fm. makes the lower portion of the Calcari Selciferi Lombardi Unit, which is part of the Upper Triassic–Lower Jurassic succession cropping out in the western Lombard Prealps (Varese province) (Figs. 1A–1C). In the “Salnova” quarry, located on the southern slope of Mt. Orsa (Figs. 1D–1E), the Saltrio Fm. reaches its maximum thickness (about 20 m) and unconformably (Wiedenmayer, 1963) does overly (some 15°) the Dolomia Principale Fm., of Triassic age, which was partly eroded under subaerial conditions at the beginning of the Jurassic (Fig. 1I). In facts, the discordance ranges in age from the Norian–Rhaetian to the early lower Sinemurian (Leuzinger, 1926; Van Houten, 1929; Gnaccolini, 1964; Kalin & Trumpy, 1977, and references therein). On the top of the Saltro Fm., it is the dark-gray Moltrasio Limestone Fm. (Stoppani, 1857), also called Lombardischer Kieselkalk (Bernoulli, 1964), which documents deposition of finer sediments in a deeper basin, from the late Sinemurian on (Fig. 1K).

### Lithology and sedimentology

Kalin & Trumpy (1977) recognized four lithofacies in the Saltrio Fm., all mostly consisting of litho-bioclastic calcarenites rich in crinoid remains, gray-brown and sometimes greenish in color, with grainstone–packstone microfacies embedding ooliths, peloids, and bioclasts. Extraclasts, consisting of reworked penecontemporaneous shallow-water dolomitic and phosphatic grains eroded from the Triassic substratum, are also present.

### Age

The Simenurian age of the Saltrio Fm. is well-supported by a hundred species of marine invertebrates, among which 19 ammonites are index fossils of that time (Sacchi Vialli, 1964; Jadoul et al., 2005). The stratigraphic position of specimen MSNM V3664 was confirmed in situ by the co-occurrence, in the bank embedding the bones, of the ammonite *Paracoroniceras* cf. *gmuendense* (Fig. 1F) and the nautiloid *Cenoceras striatum* (Fig. 1G) (V. Pieroni, 2017, personal communication), whose association is typical of the layers S3 and S5 (*sensu* Sacchi Vialli, 1964) of the Saltrio Fm., that is, of the *bucklandi* and *semicostatum* Zone. Of the two layers, according to the authors who investigated the Saltrio Fm. in the past decades (Sacchi Vialli, 1964; F. Jadoul, 2004, personal communication; Croce, 2005), the S3 is the only one containing glauconite as accessory mineral, therefore there is no doubt that the theropod bones were embebbed in the *bucklandi* Zone, which is then referable, more precisely, to the earliest portion of the early Sinemurian substage (199.3–197.5 Mya) (Ogg & Hinnov, 2012).

### Depositional environment

In the Saltrio quarry, the sedimentary succession shows a deepening-upward trend, but it lacks frankly shallow-marine sedimentary structures (e.g., shoreface facies) at the base. In facts, the stratigraphic transition is from the unconformity on Upper Triassic deposits to dolomitic breccias with green marly matrix, which represent debris flow deposits, thus already subtidal conditions (Croce, 2005). In other words, the depositional environment of the Saltrio Fm. was a likely tectonic slope that connected differently subsiding areas. After long subaerial exposure, these areas became subject to intense rifting and sunk. Due to these tectonics, the shore facies were bypassed and a subtidal environment was established directly, with debris flow deposits supplied by active tectonic slopes (M. Croce, 2018, personal communication). The texture and irregular thickness of the Saltrio Fm., the sedimentological data, and the presence of normal-salinity marine biofacies in the bone-bearing layer, with abundant crinoids, outer-shelf lagenids, and benthic foraminifera, indicate that the depositional environment of the Saltrio theropod was a proximal slope or ramp, that is, an open subtidal zone reached by the effects of storm waves and with constant bottom currents, where re-sedimentation phenomena were frequent (Jadoul et al., 2005; Croce, 2005). A depth of some dozen of meters can be reasonably estimated (F. Berra, 2018, personal communication). The parautochthonous glauconite (*sensu* Amorosi, 1997) indicates intervals of reduced sedimentation, in sectors adjacent to the seafloor where the dinosaur carcass deposited (F. Berra, 2018, personal communication).

## Taphonomy of the Saltrio Theropod

### Encasing sediment

Specimen MSNM V3664 comes from the lower banks of the Saltrio Fm., which are characterized by abundant inclusions of glauconite (Fig. 1H), a green-colored iron potassium phyllosilicate which is considered a bathymetric indicator, as it originates typically in shallow marine depositional environments, during periods of slow rates of accumulation (Amorosi, 1997). Thin sections of the layer embedding the bones (Fig. 1J) show bioclastic packstone and grainstone, with abundant and sometimes large fragments of crinoids, echinoids, ostracods, brachiopods, bivalves, gastropods, and benthic foraminifers (F. Berra, 2018, personal observation). The skeletal grains are often rounded and sometimes micritized, which indicates the presence of continuous reworking bottom currents.

### Taphonomical description

**Figure 2:**
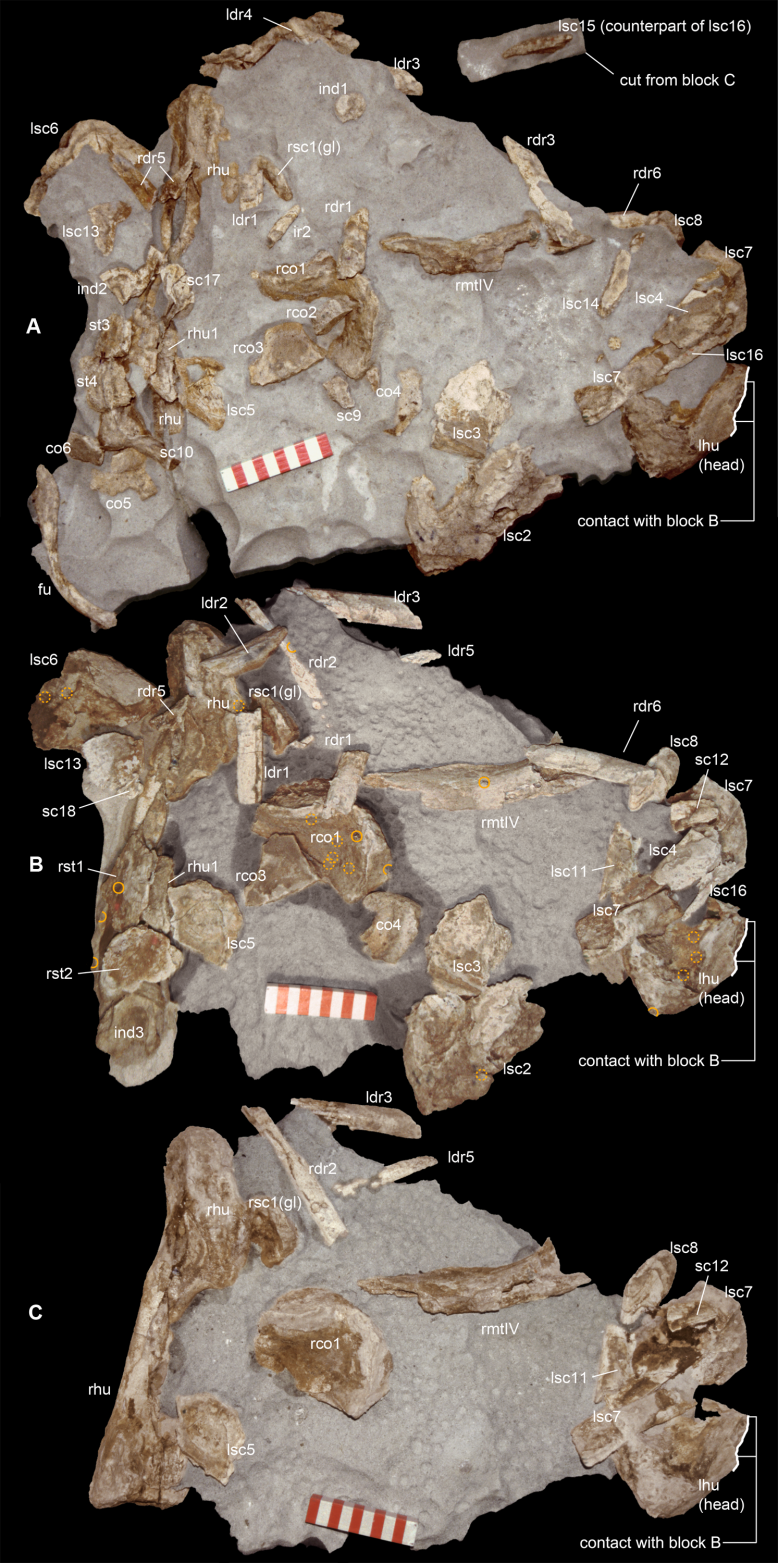
Taphonomy of the Saltrio theropod (block A). Bones of *Saltriovenator* mapped in temporal sequence (A–C), gradually emerging from the embedding rock during acid preparation of block A. Numbers refer to each fragment, not to a specific anatomical position. The latter is reported in other figures, for fragments that were later reconnected into more complete bones. Abbreviations as in text, and as follows: ind, indeterminate bone; ir, indeterminate rib; l (left) and r (right) are specified for fragments of paired bones certainly (appendicular elements) or tentatively (ribs) positioned in the skeleton. Macroborings facing front, side and back are mapped respectively with yellow circles, semicircles, and hatched circles. Scale bars equal 10 cm. Photos by G. Bindellini and C. Dal Sasso.

**Figure 3:**
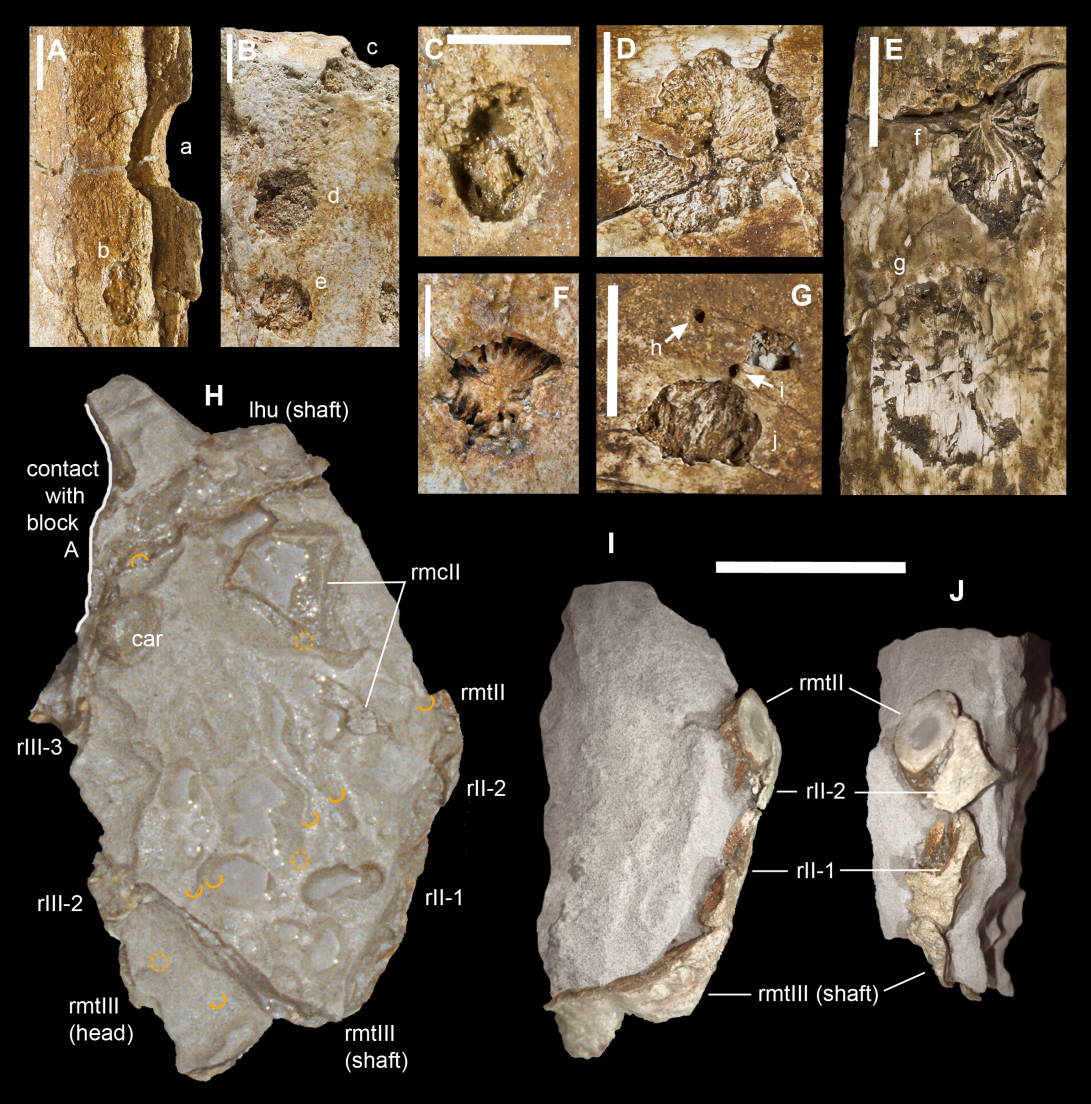
Macroborings, and taphonomy of the Saltrio theropod (block B). Selected macroborings (A–G) on bones from blocks A and B, and bones of *Saltriovenator* mapped in temporal sequence (H–J), gradually emerging from block B during acid preparation. (A) Semicircular feeding mark (a) splitted after diagenetic crushing of the bone (right metatarsal II); elliptical and flat-bottomed boring (b). (B) Feeding mark trenching the left humeral head (c); subcircular boring (d) and feebly septate circular boring (e). (C) Elliptical boring with deepening edges and a central peak on the shaft of the left humerus. (D) Circular boring with radial waves on the right humerus. (E) Dorsal rib with wavy, markedly septate elliptical boring (f) and feebly septate circular mark with zigzagging margin (g). (F) Asterisk-like septate boring on the shaft of right metatarsal II. (G) Tiny burrows penetrating the cortex of the right coracoid (h, i), and an enigmatic boring with irregular bottom and margins (j). Abbreviations as in text, symbols as in Fig. 2. Scale bars equal 10 mm in (A)–(E) and (G), five mm in (F), and 10 cm in (1)–(3). Photos by G. Bindellini, C. Dal Sasso, and M. Zilioli.

The blocks embedding the dinosaur, photographed during the acid preparation stages (Figs. 2 and 3), provided important taphonomical data, showing that the bones were: (1) laying in a single bedding plane and all disarticulated, albeit close one another; (2) not oriented but randomly scattered; (3) mostly broken into small pieces, but very rarely deformed by diagenesis. Of a hundred of specimens, only half a dozen of small and delicate bones have been compressed (two phalanges, rib ends, indeterminate bone laminae). Even the numerous scapular fragments, once reconnected, rendered a gentle continuous curvature, which is consistent with the shape of the left scapula naturally embracing the rib cage and with only the acromion taking a counter curve.

Long bones from both fore- and hindlimb show “coherent” anatomical proportions, which are consistent with the skeletal composition of one single individual, without any homologous overlapping element. No other vertebrate remains were found associated to this bone assemblage, except for one tooth and one jaw fragment pertaining to a bony fish. Likely, the Saltrio theropod fossilized almost in its entirety, but with some dispersal of body chunks in different clusters. This might explain why no vertebrae were found in blocks A and B, which contained mostly appendicular elements (about 85% of the total bone content). The complete lack of gastralia in the block that trapped the furcula, the pectoral girdle and the dorsal ribs, raises the suspect that the ventral dermal bones abandoned the carcass before it reached the sea bottom, when the decay gases caused the “explosion” of the abdomen and eventually its separation.

In block A—the largest and quantitatively most informative cluster of bones—the flattest bone (mostly scapular) fragments appear to be sorted as to floor the front side, and come to light contemporarily, during early preparation stages (Figs. 2A and 2B). In all likelihood, this apparent bedding plane represents the water-sediment paleosurface, thus the depositional succession must be imagined as upside down with respect to Figs. 2 and 3. In facts, the largest and most irregularly shaped bone (right humerus) covers the flooring fragments on the “back” side.

Furcula excluded, the bones of the pectoral girdle have suffered intense longitudinal and mosaic cracking (*sensu* Behrensmeyer, 1978) and moderate to intense abrasion (stage 1–2 of Boessenecker, Perry & Schmitt, 2014). Such a high degree of fragmentation, coupled with the taphonomical observations above listed, and the paleontological content and the sedimentology of the Saltrio calcarenites, suggests that the dinosaur carcass floated, entered a marine basin and sunk to the bottom not far from the shoreline, then decayed in shallow waters, remaining on the sea bottom for quite a long time before being completely buried. This hypothesis is further supported by a relevant taphonomical evidence: several bones of the Saltrio theropod suffered bioerosion, mostly by marine invertebrates.

#### Macroborings on the bones

A minimum of 30 certain macroborings (*sensu* Wilson, 2007) are present, which are distinguished from other doubtful traces (physical abiological damages, known as pseudoborings) by their regular semicircular contour (a in Fig. 3A) or cross-section (c in Fig. 3B), circular to elliptical shape with sharp subvertical edges and more or less flat-bottomed (b in Fig. 3A and d in Fig. 3B), or with deepening edges and a central peak (Fig. 3C), and in some cases by a peculiar differential bioerosion that produced radial-wavy (e in Figs. 3B, 3D and 3E) or asterisk-like excavations (Fig. 3F).

Three types of borings have been recognized, differing in size, shape and position with respect to the bony substrate: (1) semicircular traces produced in sharp edges of bones, with depth half than width, and dimensions ranging 15–18 mm longitudinally and five to eight mm perpendicularly to the bone edge (a in Fig. 3A and c in Fig. 3B); (2) circular wide and shallow traces produced on flat bone surfaces, with diameter ranging 8–20 mm, and one to four mm deep (b in Fig. 3A, d–e in Figs. 3B, 3C and 3F); (3) tiny holes (0.5–1 mm), penetrating the bone cortex (i in Fig. 3G). The first type can be interpreted as a feeding structure, likely produced by vertebrate jaws gnawing the bone edge, a praedichnia in the sense of Gibert, De Domenech & Martinell (2004). The second type of trace is interpreted as an anchorage trace, or fixichnia (Gibert, De Domenech & Martinell, 2004; Bromley & Heinberg, 2006) of unknown invertebrates (probably, more than one taxon). The third type can be referred to the ichnogenus *Sedilichnus* (Zonneveld & Gingras, 2014) and could be a fixichnia, a permanent dwelling structure (domichnia), or a structure produced by a worm-like animal during osteophagy (praedichnia).

### Taphonomical interpretation

A map of the macroborings on the bones in situ (Figs. 2 and 3) shows that 27 of 30 marks faced the back (*n* = 15) and side (*n* = 12) directions, and only three marks faced the front of blocks A + B. This distribution confirms that the bedding layer was upside down with respect to Figs. 2 and 3, and that the bones of the Saltrio theropod remained exposed for most of their surface to bottom currents and scavengers, which easily rolled the elements with rounded cross-sections, in this case favoring the marks on multiple sides (e.g., humeri and metatarsals). The evaluation of the exposure time depends also on the estimated grazing and colonizing speed of the bone tissue, thus on the scavenging fauna (Boessenecker, Perry & Schmitt, 2014).

The identification of the tracemakers is beyond the aims of this study; however, it is worth to note that this is likely the first record of marine bioerosions on dinosaur bones. In turn, it is well-documented that whale falls at the sea floor can nourish subsequent communities of scavengers for several years (Smith & Baco, 2003), and there is evidence of the same processes in the fossil record of cetaceans (Dominici, Danise & Benvenuti, 2018), plesiosaurs (Kaim et al., 2008), and ichthyosaurs (Danise, Twitchett & Matts, 2014).

Similarly, necrophagy on the bones of the Saltrio theropod by a variety of taxa indicates that the dinosaur carcass remained exposed to the water-sediment interface for months, maybe years, long enough to being first defleshed by mobile scavengers, then colonized by a microbial community that spanned the bone–water interface, which in turn attracted slow-moving grazers and epibionts. The bones of the dinosaur were locally bioeroded by these opportunistic macroinvertebrates, furthermore fragmented, and partially abraded by the bottom currents and the sandblasting action of the calcarenites, which eventually covered them.

The fact that the main scavengers of the Saltrio theropod were benthic marine invertebrates is a further confirmation that the dinosaur carcass deposited on a well-oxygenated and well-illuminated sea bottom, in any case comprised within the photic zone, where the biotic activity was intense but, at the same time, the sedimentation rate was high enough to cover skeletal material before its complete destruction (Dominici, Danise & Benvenuti, 2018). In our material, this sequence of events (i.e., partial scavenging followed by burial and diagenesis) is best documented by a deep semilunate “bite,” produced along the shaft of metacarpal II (Fig. 3A). The gnawing action trenched a perfect semicircle; much later, the edge of the bite was splitted in two by subsequent collapse of the bone wall onto the hollow central cavity, caused by diagenetic pressure of the sediment that accumulated on top.

## Paleobiogeographical Remarks

According to recent geological studies (Jadoul et al., 2005, and reference therein), from Hettangian to earliest Sinemurian times the Early Jurassic paleogeography of the western Lombardy Basin was dominated by a continental area that was wider than previously thought, and characterized by a warm humid paleoclimate. The nearest emerged land which the carcass of *Saltriovenator* could maybe come from, was the Arbostora swell (Kalin & Trumpy, 1977), a structural high close to the Saltrio area, which divided the subsiding basins of Mt. Nudo (East) and Mt. Generoso (West). The Arbostora swell was settled on a carbonate platform that emerged with other wider areas, in the west to southeast, bordering a shallow-water gulf that deepened northwards. A horst and graben tectonic setting controlled the alternated distribution of these marine and terrestrial environments.

Unconformities with “terra rossa” paleosoils (Leuzinger, 1926; Van Houten, 1929; Wiedenmayer, 1963; Gnaccolini, 1964; Kalin & Trumpy, 1977), including one outcropping at Castello Cabiaglio-Orino, a dozen of kilometers West of Saltrio (Jadoul et al., 2005), testify that the emerged areas located in the southern and western sectors of the present Maggiore Lake were covered with forests. This reconstruction is supported by the occurrence of large plant fragments, immediately above the unconformities and in the basal Moltrasio Fm. (Jadoul et al., 2005). Most of these fossils have been found between Cellina and Arolo, along the eastern side of Lake Maggiore, in a stratigraphic succession that turned out to be coeval to the basal Saltrio Fm. (Lualdi, 1999), that is, to the dinosaur-bearing strata. Lualdi (1999) found and described a varied flora, which is quite informative in paleoecological terms. In facts, the abundant plant debris fossilized in those arenitic beds included Bennettitales, with one genus (*Ptilophyllum*) that occupied the same ecological niches of the modern mangroves, frankly terrestrial Araucariaceae (*Pagiophyllum*), and Cheirolepidiaceae with small and scaly leaves (*Brachyphyllum*), which indicate inland areas with dry-warm conditions. The duration and extent of the Early Jurassic emersion in the western Lombardy Basin cannot be assessed precisely, and paleogeographic relationships at larger scale are even more difficult to assess (we can only tell that this region was closer to southern Laurasia than to northern Gondwana—Scotese, 2014). However, as stated above, there is compelling evidence that emerged areas were present in the late Hettangian-earliest Sinemurian, with local emersion stages starting, on structural highs, during the late Rhaetian and the early Hettangian (Bernoulli, 1964), and that the region became a subsiding basin only in the late early Sinemurian (Kalin & Trumpy, 1977).

Detailed stratigraphic prospections in and around the Saltrio area (Croce, 2005) indicate that the paleogeography of the Arbostora swell was initially (Norian–Rhaetian) characterized by shallow marine peritidal–subtidal environments, with more protected areas (lagoons, bays) receiving terrigenous contributions from a portion of platform (Mt. Orsa) that, as testified by the sedimentary gap of the Dolomia Principale underlying the Saltrio Fm., was already emerged. Later, from the entire Hettangian up to the earliest Sinemurian (i.e., for 3 million years), the whole Arbostora swell emerged and became a barrier between the Mt. Nudo and Mt. Generoso basins. In the early Sinemurian, the swell became again a shallow open sea (ramp-slope), still surrounded South and South-West by emerged land. In this period the holotype of *Saltriovenator* lived and died, and luckily its bones flowed into a gulf of the Mt. Nudo basin, where they became fossilized. On top of them, in the late Sinemurian, the Moltrasio Limestone accumulated: the area became a deeper basin with emipelagic sedimentation, and the Arbostora swell became fused to the two adjacent basins (Mt. Nudo and Mt. Generoso).

With regards to the land extent, it is worth to note that a regressive trend, with large emerged areas and karstified surfaces since Hettangian times, has been proposed by Pasquini & Vercesi (2002) in some sectors of the “Triangolo Lariano” (Corni di Canzo-M. Cornizzolo). A local emersion area, documented by inter-supratidal horizons, was certainly present in the eastern Lombardy high (Jadoul et al., 2005). Moreover, a major Early Jurassic emerged area was located between the Lake Maggiore and the Lombardy plane southwards, up to Monza (Pieri & Groppi, 1975, 1981). To the west, this continental area extended to the Mt. Fenera high, and possibly up to the Canavese Zone (Bernoulli et al., 1979). If those structural highs were really connected, as Jadoul et al. (2005: fig. 189) seem to conclude, then those lands were certainly enough vast to sustain >7-m-long predatory dinosaurs, and the trophic chain connected to them, which may imply the presence of herbivorous vertebrates and plant communities. In the end, there is not even the need for hypothetical (and quite unlikely) continental bridges, to ask oneself whether or not the western Lombardy continental areas were linked to the contemporary terrestrial habitats of the Trento Platform, where, based on a number of famed ichnosites, a variety of dinosaurs, including theropods the size of *Saltriovenator*, was certainly roaming (Petti et al., 2011, and references therein). Indirect size correlation with the abundant and coeval large theropod tracks from NE Italy suggests that our new taxon could have been among the most common trackmakers in the Early Jurassic shoreline habitats of western Tethys.

## Systematic Paleontology

DINOSAURIA Owen, 1842
THEROPODA Marsh, 1881
NEOTHEROPODA Bakker, 1986
CERATOSAURIA Marsh, 1884
*Saltriovenator zanellai* gen. et sp. nov.
LSID urn:lsid:zoobank.org:act:8C9F3B56-F622-4C39-8E8B-C2E890811E74 (*Saltriovenator*)
LSID urn:lsid:zoobank.org:act:BDD366A7-6A9D-4A32-9841-F7273D8CA00B (*Saltriovenator zanellai*)

**Etymology.** *Saltrio*, Italian toponym name, from the locality where the holotype was found; *venator*, Latin word for hunter, it also refers to a type of Roman gladiator; *zanellai*, Latin genitive dedicated to Angelo Zanella, who discovered the fossil.

**Figure 4:**
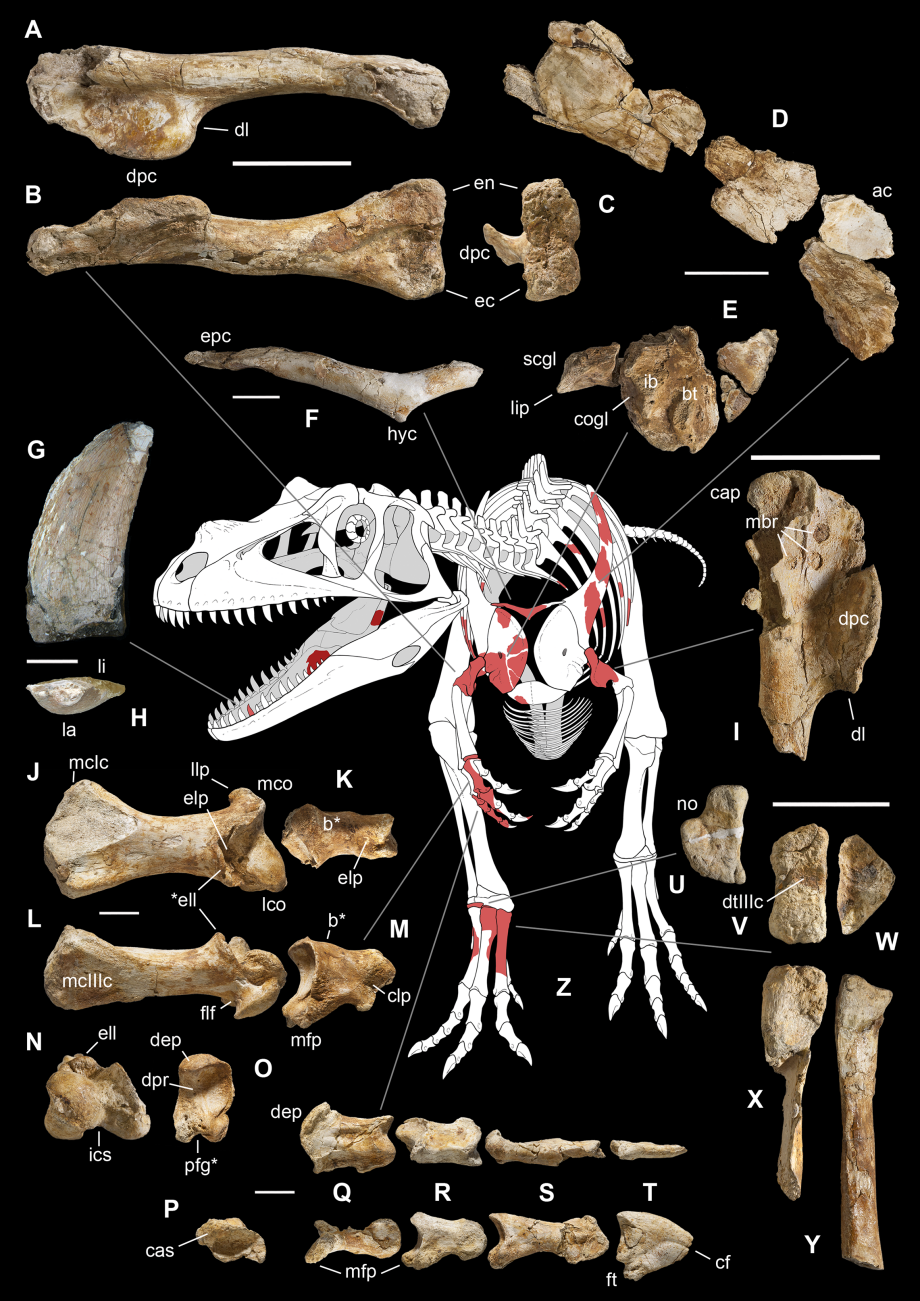
Selected elements used in the diagnosis of *Saltriovenator zanellai* n. gen. n. sp. Right humerus in medial (A), frontal (B) and distal (C) views; (D) left scapula, medial view; (E) right scapular glenoid and coracoid, lateral view; (F) furcula, ventral view; tooth, labial (G) and apical (H) views; (I) left humerus, medial view; right second metacarpal in dorsal (J), lateral (L) and distal (N) views; first phalanx of the right second digit in dorsal (K), lateral (M) and proximal (O) views; (P–T) right third digit in proximal, dorsal and lateral views; (U) right distal tarsal IV, proximal view; third right metatarsal in proximal (V) and frontal (X) views; second right metatarsal, proximal (W) and frontal (Y) views; (Z) reconstructed skeleton showing identified elements (red). Abbreviations as in text, asterisks mark autapomorphic traits. Scale bars: 10 cm in (A)–(E), (I), and (U)–(Y); two cm in (F), and (J)–(T); one cm in (G). Photos by G. Bindellini, C. Dal Sasso and M. Zilioli; drawing by M. Auditore.

**Figure 5:**
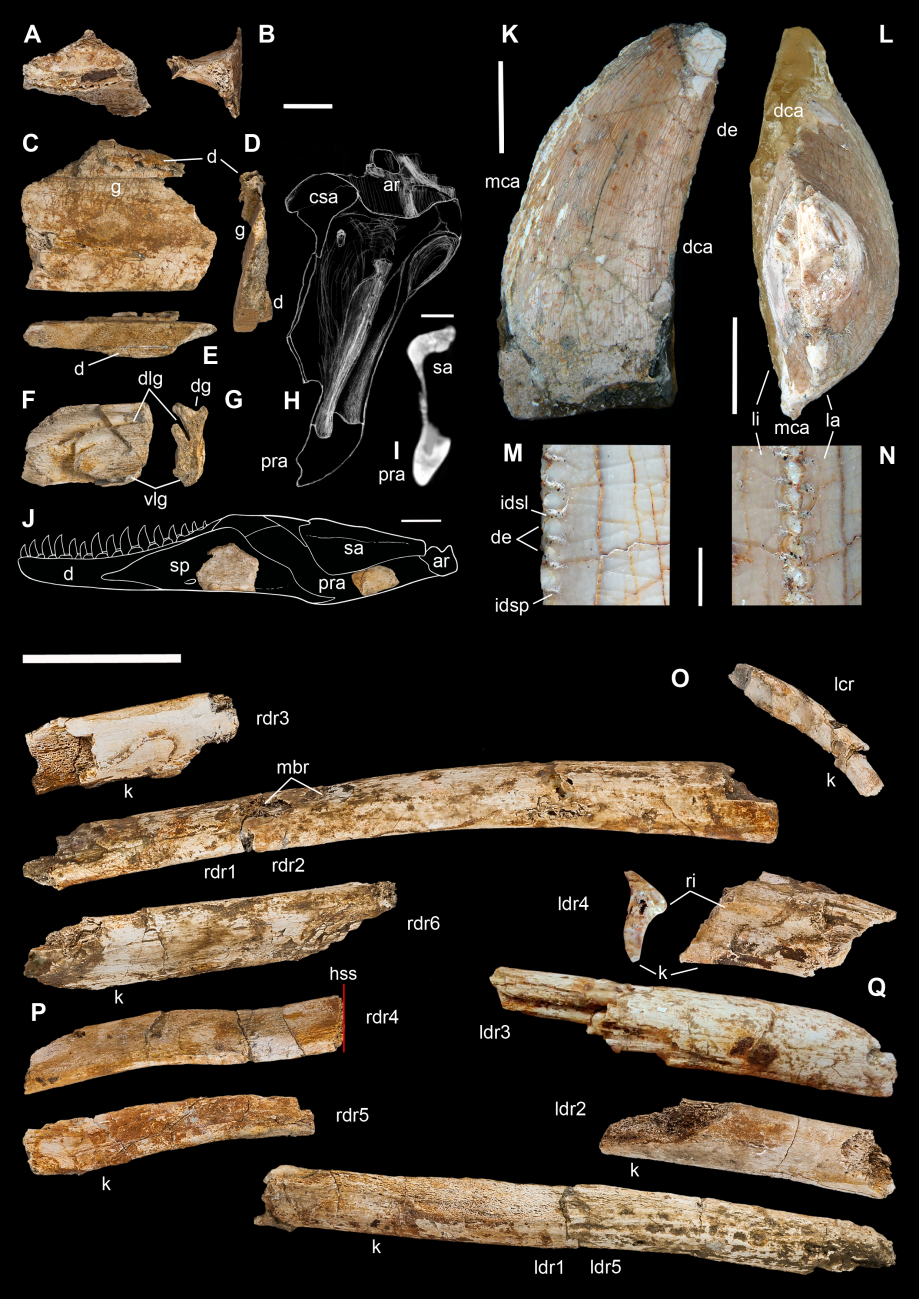
Cranio-mandibular fragments, tooth, and ribs of *Saltriovenator zanellai*. Indeterminate cranial fragment (A–B); right splenial in lateral (C), rostral (D) and ventral (E) views; right prearticular in lateral (F) and rostral views (G); sketch of the right prearticular of MOR 693 (*Allosaurus fragilis*) with virtual cross-section (H) diagnostic for G, also confirmed by CT slicing of the left side element of MOR 693 (I); splenial and prearticular in medial view, positioned in a reconstructed right lower jawof *Saltriovenator* (J). Maxillary or dentary tooth in labial (K) and apical (L) views; close-up of the distal carina and denticles in lingual (M) and distal (N) views. Left cervical rib (O) in craniolateral view; fragmentary right (P) and left (Q) dorsal ribs in craniolateral view. Abbreviations as in text, ribs labeled as in Fig. 2 maps and caption. Scale bars equal two cm in (A)–(I), five cm in (J), one cm in (K), five mm in (L), one mm in (M)–(N), five cm in (O)–(Q). Photos by G. Bindellini, C. Dal Sasso, and M. Zilioli; drawing by C. Dal Sasso.

**Figure 6:**
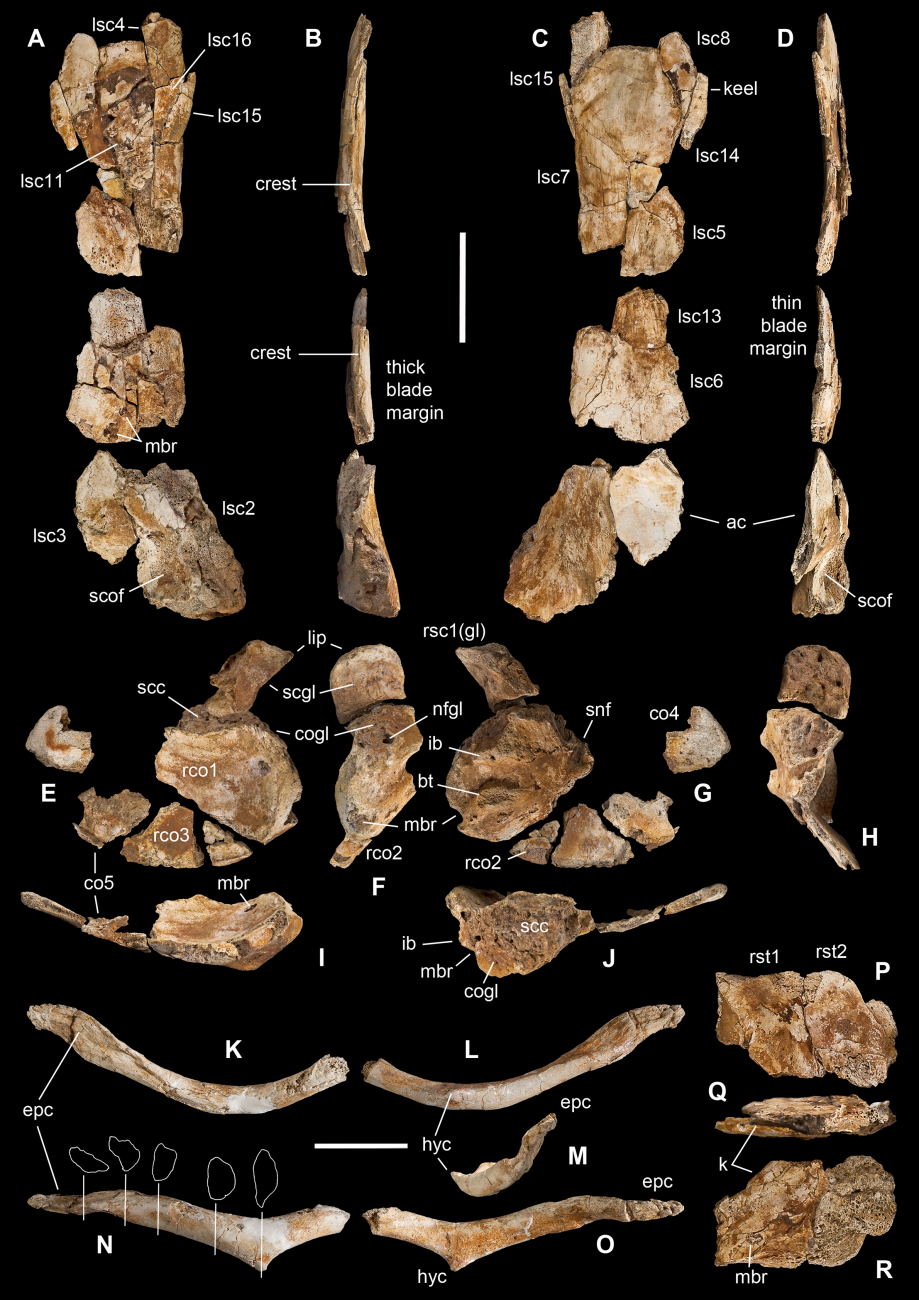
Pectoral girdle of *Saltriovenator zanellai*. Left scapula in lateral (A), caudal (B), medial (C), and cranial (D) views; right scapular glenoid and coracoid in medial (E), caudal (F), lateral (G), and cranial (H) views; right coracoid in ventral (I) and dorsal (J) views; furcula in cranial (K), caudal (L), right lateral (M), ventral (N, with selected craniocaudal cross-sections), and dorsal (O) views; caudolateral portion of the right sternal plate in dorsal (P), lateral (Q), and ventral (R) views. Each bone fragment is labeled on the side cropping out in Fig. 2. Abbreviations as in text. The position of co4 and co6 is hypothetical. Scale bars equal 10 cm in (A)–(J), five cm in (K)–(R). Photos by G. Bindellini and C. Dal Sasso; drawings by M. Auditore.

**Figure 7:**
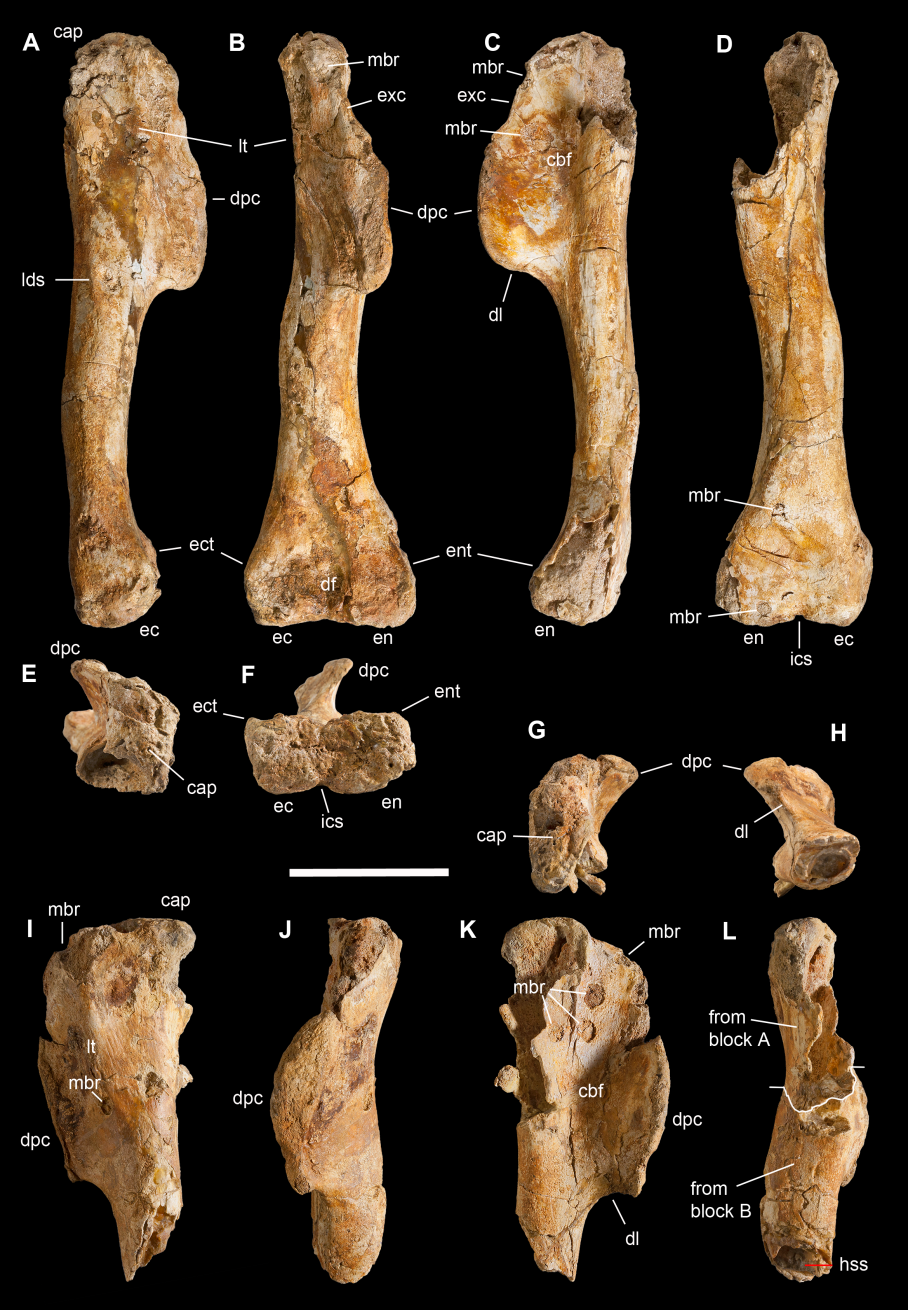
Humeri of *Saltriovenator zanellai*. Right humerus in (A) lateral, (B) cranial, (C) medial, (D) caudal, (E) proximal, and (F) distal views; left humerus in (G) proximal, (H) distal, (I) lateral, (J) cranial, (K) medial, and (L) caudal views. Abbreviations as in text. Scale bar equals 10 cm. Photos by G. Bindellini.

**Figure 8:**
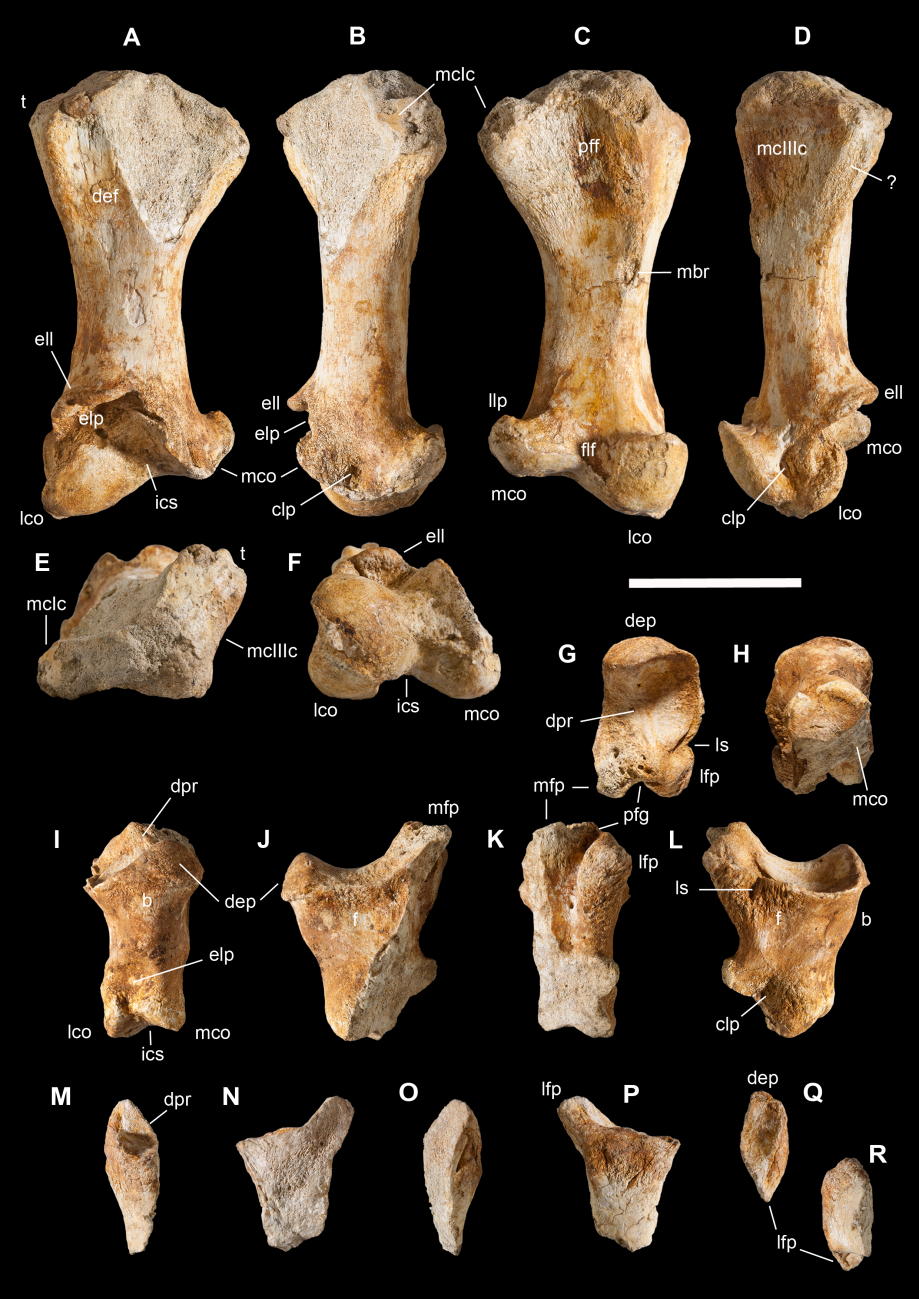
Manus of *Saltriovenator zanellai*, second metacarpal and digit. Right second metacarpal (II) in (A) dorsal, (B) medial, (C) palmar, (D) lateral, (E) proximal, and (F) distal views; first phalanx of the right second digit (II-1) in (G) proximal, (H) distal, (I) dorsal, (J) medial, (K) palmar, and (L) lateral views; second phalanx of the right second digit (II-2) in (M) dorsal, (N) medial, (O) palmar, (P) lateral, (Q) proximal, and (R) distal views;. Abbreviations as in text. Scale bar equals five cm. Photos by G. Bindellini.

**Figure 9:**
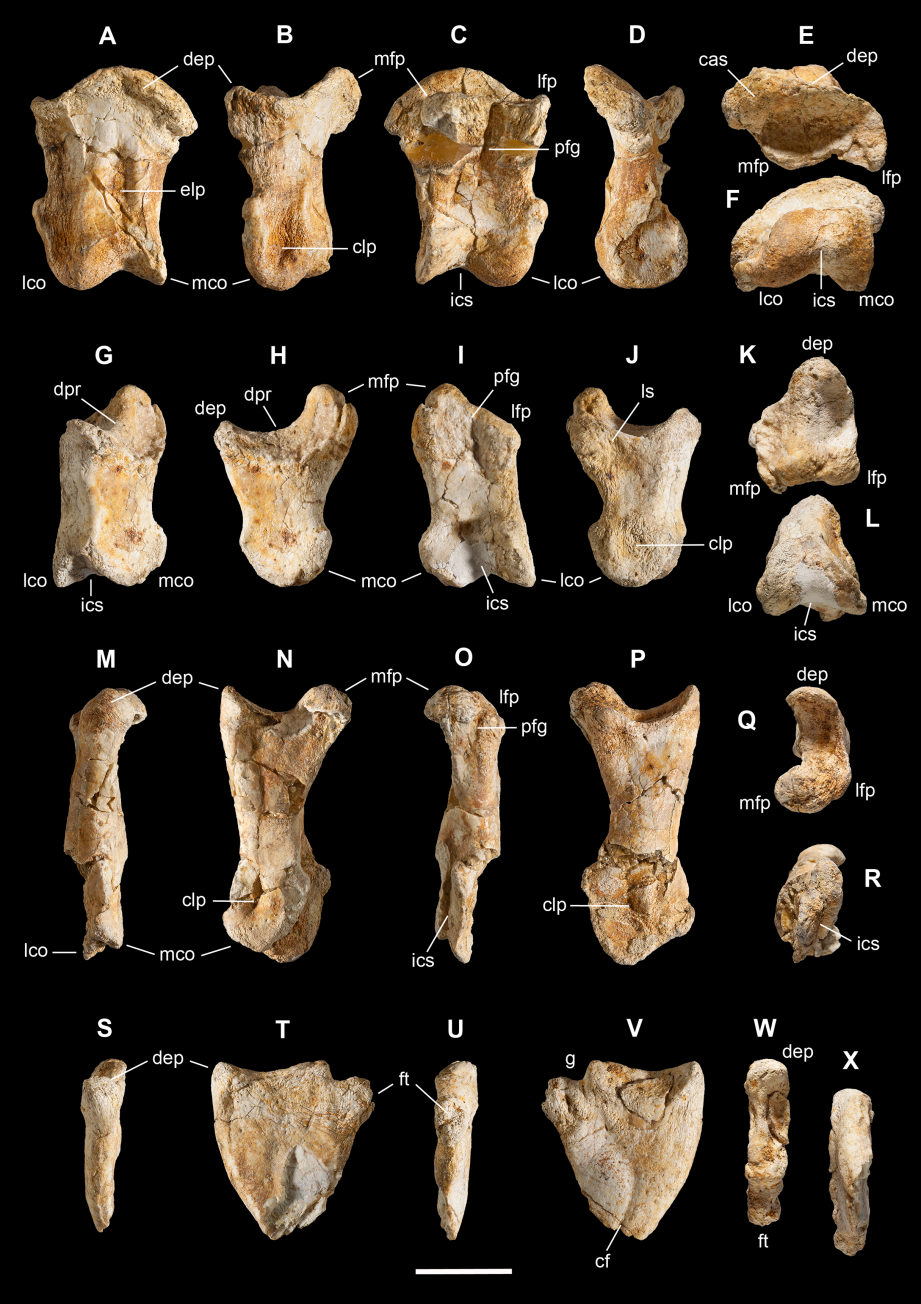
Manus of *Saltriovenator zanellai*, third digit. Right first phalanx (III-1) in (A) dorsal, (B) medial, (C) palmar, (D) lateral, (E) proximal, and (F) distal views; right second phalanx (III-2) in (G) dorsal, (H) medial, (I) palmar, (J) lateral, (K) proximal, and (L) distal views; right third phalanx (III-3) in (M) dorsal, (N) medial, (O) palmar, (P) lateral, (Q) proximal, and (R) distal views; right fourth (ungual) phalanx (III-4) in (S) dorsal, (T) medial, (U) palmar, (V) lateral, (W) proximal, and (X) distal views. Abbreviations as in text. Scale bar equals two cm. Photos by G. Bindellini.

**Figure 10:**
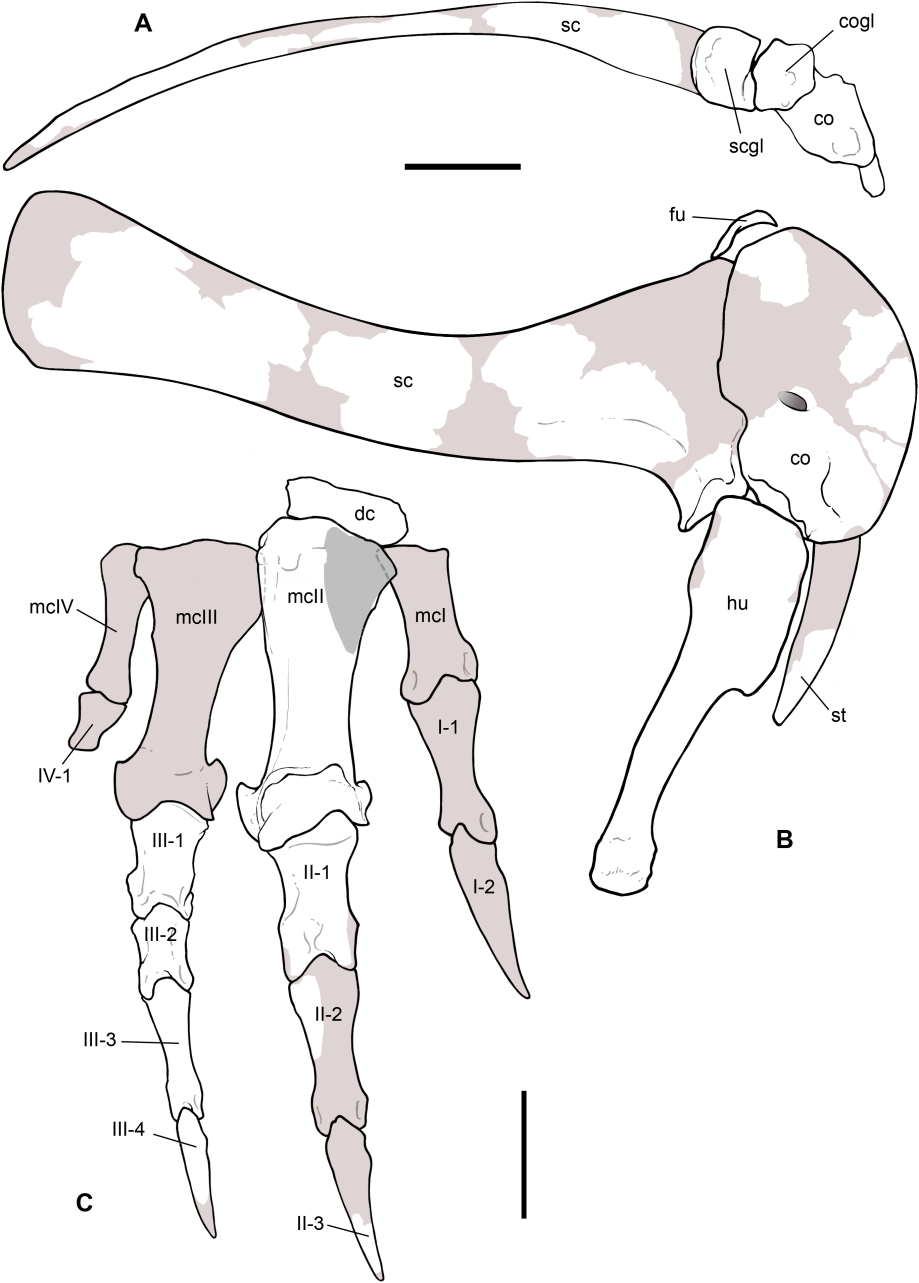
Reconstruction of the pectoral girdle and forelimb of *Saltriovenator zanellai*. Composite right scapula and coracoid in ventral view (A), and composite right pectoral girdle, humerus and manus in lateral view (B–C). The scapular body and blade, and the proximal portion of the humerus are reversed left elements. Preserved elements in white, reconstructed bone in light gray, exposed inner bone in gray, hidden bone in dotted lines. Abbreviations as in text. Scale bar equals 10 cm in (A) and (B), five cm in (C). Drawings by M. Auditore.

**Figure 11:**
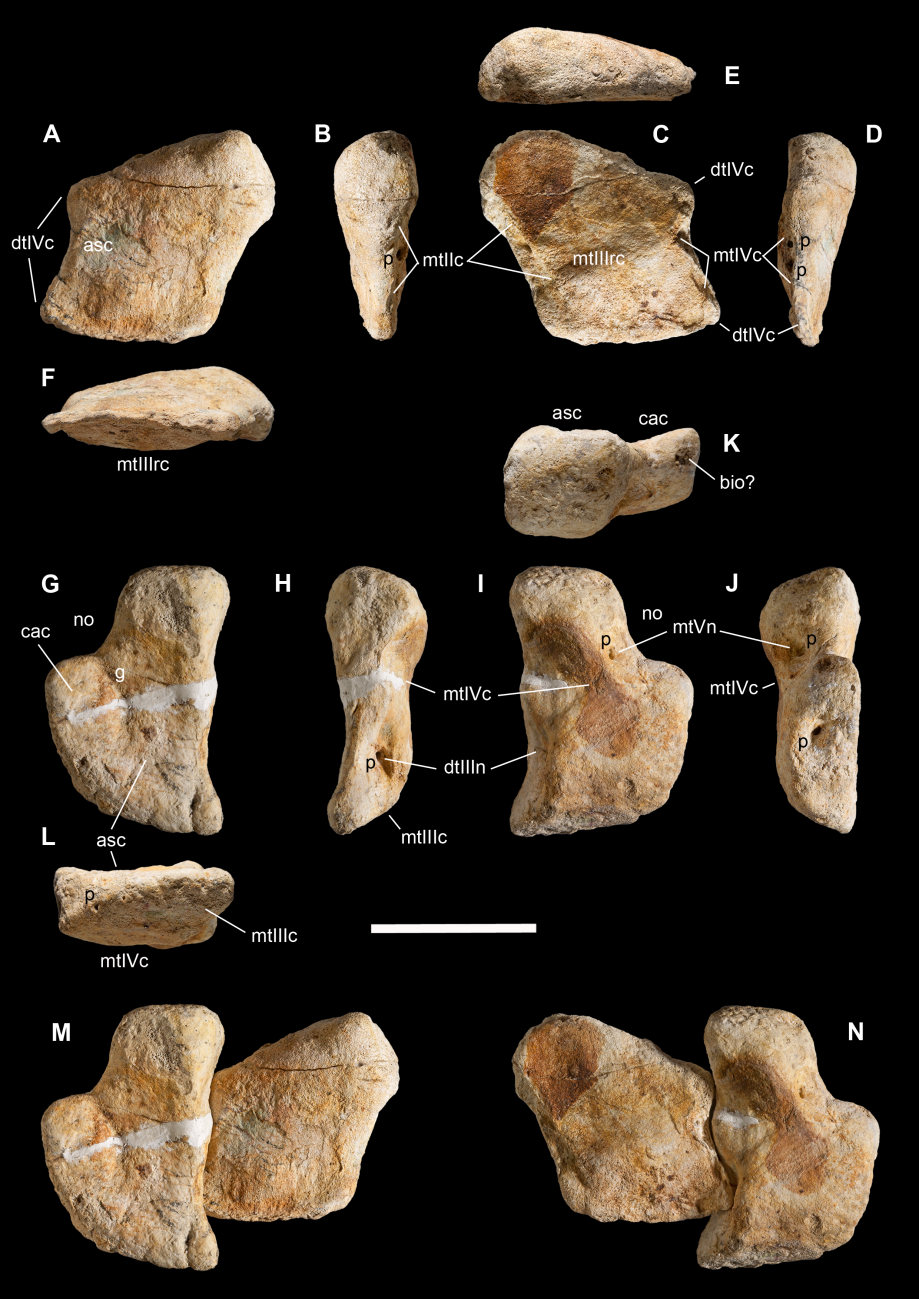
Distal tarsals of *Saltriovenator zanellai*. Right distal tarsal III in (A) proximal, (B) medial, (C) distal, (D) lateral, (E) caudal, (F) cranial views; right distal tarsal IV in (G) proximal, (H) medial, (I) distal, (J) lateral, (K) caudal, (L) cranial views; articulated right distal tarsals III + IV in proximal (M) and distal (N) views. Abbreviations as in text. Scale bar equals five cm. Photos by G. Bindellini.

**Figure 12:**
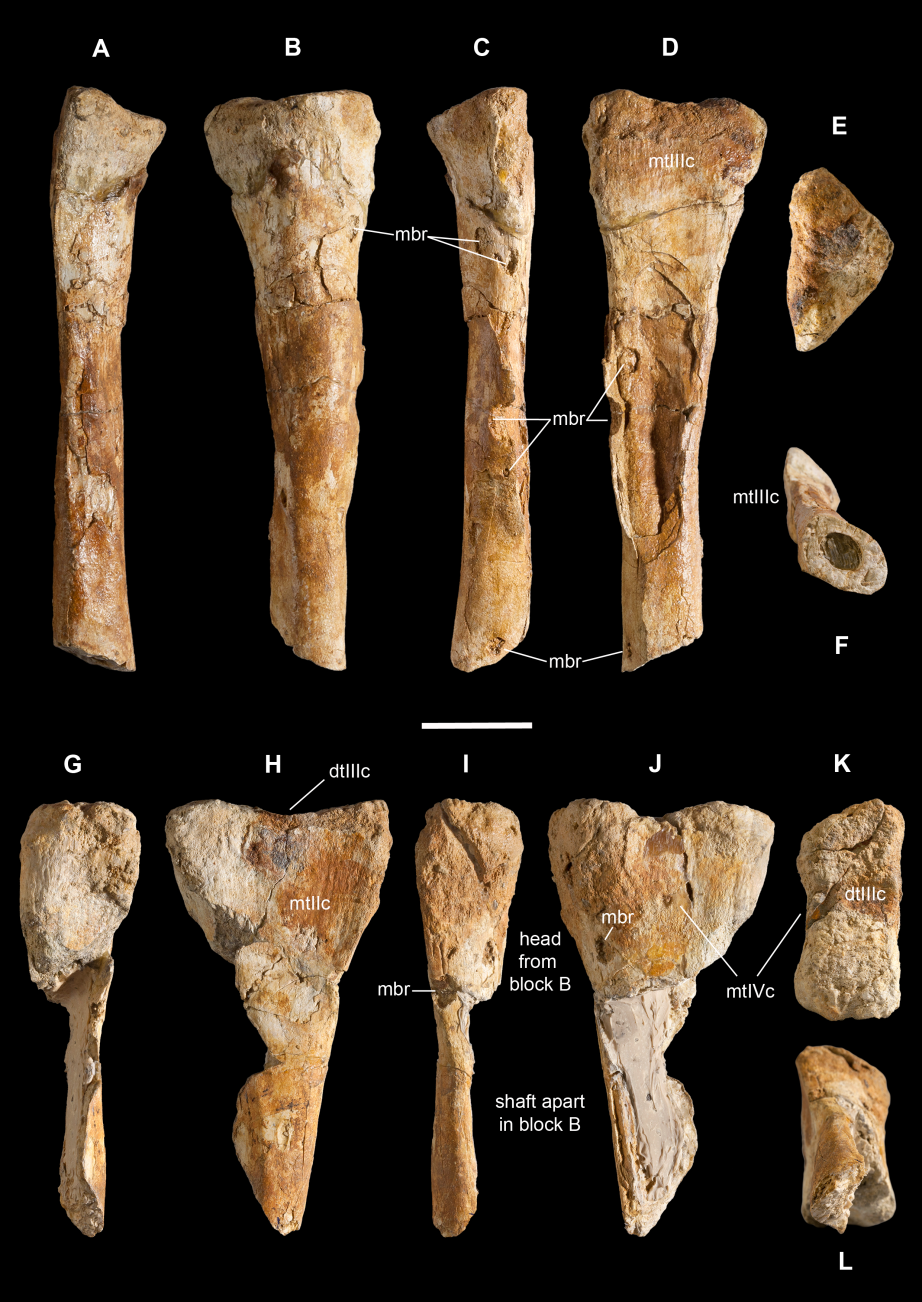
Metatarsals II and III of *Saltriovenator zanellai*. Right metatarsal II in (A) cranial, (B) medial, (C) caudal), (D) lateral, (E) proximal, and (F) distal views; right metatarsal III in (G) cranial, (H) medial, (I) caudal), (J) lateral, (K) proximal, and (L) distal views. Abbreviations as in text. Scale bar equals five cm. Photos by G. Bindellini.

**Figure 13:**
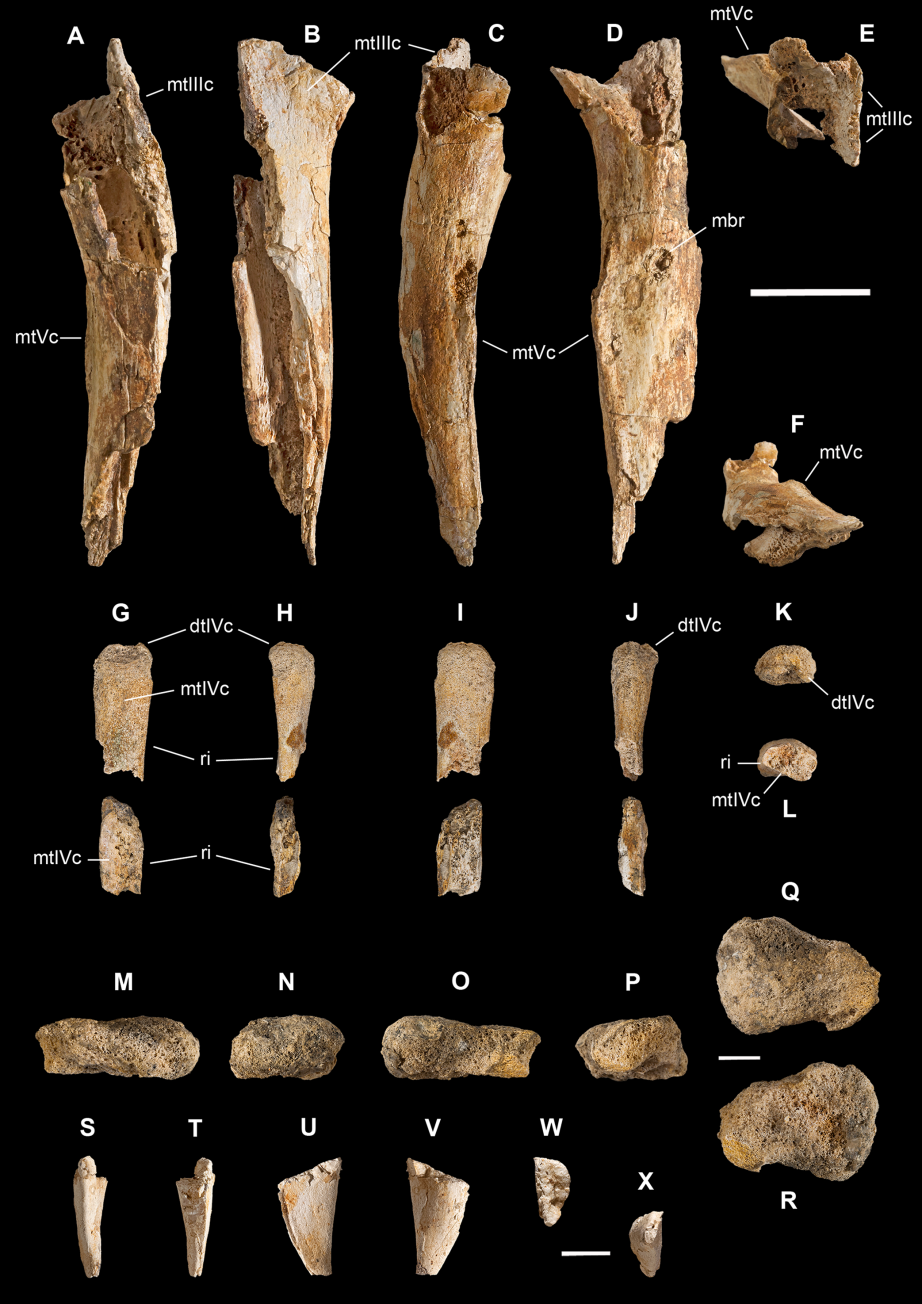
Metatarsals IV and V, carpal and ungual of *Saltriovenator zanellai*. Right metatarsal IV in (A) cranial, (B) medial, (C) caudal), (D) lateral, (E) proximal, and (F) distal views; right metatarsal IV in (G) cranial, (H) medial, (I) caudal), (J) lateral, (K) proximal, and (L) distal views. Carpal bone in (M) ?cranial, (N) ?medial, (O) ?caudal), (P) ?lateral, (Q) ?proximal, and (R) ?distal views; distal portion of manual ungual phalanx (?II-3) in (S) dorsal, (T) palmar, (U) ?medial, (V) ?lateral, (W) proximal, and (X) distal views. Abbreviations as in text. Scale bar equals five cm. Photos by G. Bindellini.

**Holotype.** MSNM V3664, very fragmentary and disarticulated skeleton (Figs. 4–13), represented by the following elements (among brackets, number of fragments per bone): partial right splenial (2) and right prearticular (1); cervical (1) and dorsal (9) ribs; furcula (1), incomplete left scapula (16), right scapular glenoid (1), partial right coracoid (5), fragmentary right sternal plate (2); right humerus (2), and proximal half of left humerus (2); ?right ?distal carpal, right metacarpal II, right phalanx II-1, fragmentary right phalanx II-2, and tip of the ?second right ungual phalanx; complete third right manual digit (phalanges III-1 to III-4); right distal tarsals III and IV, proximal portions of right metatarsals II, III, IV, and V(2).

**Referred material.** MSNM V3659, one maxillary or dentary tooth (Figs. 4 and 5).

**Comments.** As noted above, the discovery of all skeletal elements at the same time in a very restricted spot, the fact that all of them are of matching size, and that fragmentary and anatomically adjacent elements are of matching morphology, leave no doubt that all bones referred to the holotype come from the same individual. We prudentially exclude from the holotype the single tooth, which was found relatively associated to the bones but lacking its root and any jaw bone connection, thus raising the doubt that it might represent a shed tooth.

**Type locality.** “Salnova” quarry, Saltrio, Varese Province, Lombardy (northern Italy).

**Horizon and Age.** Saltrio Fm. (sensu Gnaccolini, 1964), *bucklandi* Zone, early Sinemurian (199.3–197.5 mya) (Ogg & Hinnov, 2012).

**Diagnosis.** Mid-to-large sized ceratosaurian characterized by the following unique combination of anatomical features (autapomorphies marked by asterisk—see also Fig. 4): humerus with deltopectoral crest protruding craniomedially for more than twice the shaft diameter, with distal lamina forming an abrupt corner (about 90°) with the proximodistal axis of the humeral shaft; metacarpal II with hypertrofied semicircular extensor lip protruding over the condylar level* and bordering dorsolaterally a very deep and wide extensor pit; phalanx II-1 with flexor palmar groove which is deep and narrow*, and bearing a distinct bump distal to the dorsal extensor process*; manual ungual III with prominent flexor tubercle which is distinctly separated from articular facet by a concave cleft.

**Remarks.** *Saltriovenator* shares with **dilophosaurids** (e.g., *Cryolophosaurus*, *Dilophosaurus*): glenoid cavity directed mainly caudoventrally without lateral exposition; scapula and coracoid considerably thick at scapulocoracoid contact; coracoid with short and bluntly rounded caudoventral margin, and with bicipital tubercle developed as a subtriangular boss-like prominence; deep pit on dorsal end of metacarpal II allowing hyperextension of the proximal phalanx; manual phalanx III-3 longer than III-1 and 2 but shorter than their sum; distal tarsal IV bears a wing-like craniolateral margin; proximal end of metatarsal II lacks any process expanding the contact with metatarsal III; proximal ends of metatarsals II and III have a subequal transverse width.

*Saltriovenator* shares with **basal ceratosaurians** (e.g., *Ceratosaurus*, *Eoabelisaurus*): strap-like scapular blade; humerus straight in lateral view; humeral head not inflated neither dome-shaped; distal end of metacarpal II narrower than the proximal but abruptly expanded from the shaft and twisted, bearing asymmetrically-developed condyles, shelf-like margin of collateral fossae, pronounced flexor lip-and-pit complex on the dorsolateral side of metacarpal II (allowing a 65–70° hyperextension of the proximal phalanx); manual phalanges with diaphysis longer than distal epiphysis and well-developed proximal flexor processes; phalanx II-1 with dorsopalmar ridge obliquely and unequally partitioning the proximal articulation (causing a marked twisting inward of the bone axis during extension); phalanx III-1 with concavo-convex proximal articulation indicating asymmetry in the distal condyles of metacarpal III; distal tarsal IV bears a distinct subrectangular notch for metatarsal V; proximal end of metatarsal III lacks both a mediolateral plantar expansion and a middle constriction.

*Saltriovenator* shares with **abelisauroids** (including *Limusaurus*): humerus non-twisted; phalanx II-1 very short, half or less than half the length of metacarpal II, and abruptly narrower mediolaterally than the latter, with deep narrow palmar flexor groove.

*Saltriovenator* also shows the following derived features that are ambiguous apomorphies of **Neoceratosauria**: supraglenoid lip in lateral view almost hook-like; distal humeral condyles nearly flattened; deltopectoral crest longer than 45% the length of the humerus and oriented obliquely on the humeral shaft; proximal end of metacarpal I loosely appressed to metacarpal II; manual ungual phalanges with simple unforked collateral furrow.

*Saltriovenator* shares with the **basalmost tetanurans**: furcula with a distinct hypocleideum; humerus straight in lateral view; prominent quadrangular deltopectoral crest extended for about half of bone length; robust metacarpal II with enlarged distal end bearing a deep extensor pit and a robust lip.

## Description and Comparisons

### Skull

From a cranial element possibly comes a fragmentary bone with a very peculiar texture and high degree of vascularization (Figs. 5A and 5B). This bone is broken at any end, showing a T-shaped cross-section that at first glance recalls a vertebral transverse process with a deep and robust centrodiapophyseal lamina. However, the top of the T is perfectly flat and the two other bone surfaces are textured with fine ridges and pits, suggesting tight soft tissue attachments. This texture clearly differs from the parallel striations (i.e., muscle and ligament scars) seen on the vertebral processes (C. Dal Sasso & S. Maganuco, 2017, personal observation on *Allosaurus fragilis* MSNM V435). The internal structure also differs in being highly spongy rather than fibrolamellar, indicating a delicate, not robust structure. In addition, the purported centrodiapophyseal lamina widens toward its broken edge, suggesting a V-shaped branching or prosecution toward a wider portion of bone. One can hypothesize that this fragment was part of a cranial fenestra, but to relocate its anatomical position remains impossible.

### Lower jaw

Three fragments that can be referred to the lower jaw have been recovered closely associated from block B. Besides their thin bony wall and finely parallel ridged texture, oriented rostrocaudally, the three fragments share complex grooved surfaces, reminiscent of the vascular grooves that usually run along the medial and internal sides of the lower jaw bones.

**Splenial.** The largest bone piece (Figs. 5C and 5E) preserves two other small fragments in tight sutural contact, respectively, with its dorsal and ventral margin; both sutures run restrocaudally, paralleling the finely ridged texture. The main fragment has a possibly medial surface missing the cortex and exposing the internal bone structure, a ventral sharp margin, oriented at 90°, and a flat ?lateral side that houses a longitudinal groove near its dorsal end. We think that this laminar element may be part of the middle portion of a right splenial, just caudal to the Meckelian foramen (absent in our fragment), where the splenial is clasped dorsally and ventrally by the caudal ends of the bifurcating dentary.

The second jaw fragment is much narrower dorsoventrally but preserves a sharp ?ventral margin with an angle of 90°, just like the previous fragment, which suggests it might be the rostral continuation of the same bone. In facts, the splenial of coelophysoid-grade theropods (including *Dilophosaurus*) is more elongate and rostrally tapering than that of tetanurans like *Allosaurus*. Interestingly, the splenial of *Ceratosaurus nasicornis* (C. Dal Sasso, 2017, personal observation on AMNH FR 27631- cast of the right lower jaw of USNM 4735) at mid-length displays a labioventral margin which is sharp-squared, highly similar to the margin of our fragments.

**Prearticular.** The third jaw fragment (Figs. 5F–5G) is here interpreted as a piece of the right prearticular, thanks to its very peculiar cross-section. The medial side is slightly convex and the lateral side is slightly concave, with the same curvature; the narrow ventrolateral and dorsal margins house a shallow groove each, whereas dorsolaterally a deep narrow groove enters the bone until the middle, giving its dorsal section a Y-shaped aspect. Such complex profile was used as a fingerprint to relocate the anatomical position of this bone fragment on complete theropod skulls and lower jaws. The best match occurred with the lower jaw of *Allosaurus* MOR 693 (C. Dal Sasso, 2004, personal observation). Carefully examining its disarticulated bones, we found an almost identical arrangement of grooves and processes at mid-caudal length of the right prearticular (Fig. 5H). That diagnostic cross-section, inferred by manual drawing, was later confirmed by unpublished CT data of the same specimen (E. Rayfield, 2016, personal communication; Fig. 5I). The prearticular of *Ceratosaurus*, “in so far as one may judge from the parts preserved it is very similar to that of *Antrodemus*” (Gilmore, 1920). In facts, the Saltrio fragment matches the prearticular of MOR 693 even in size (both are 35 mm tall), thus it is consistent with a lower jaw about 80 cm long (Fig. 5J), and a body length of a subadult *Allosaurus fragilis* (see below).

Yates (2005: fig. 5C, D) illustrates and describes a fragment “from near the posterior end” of the right prearticular of *Dracovenator regenti* that further confirms our interpretation: “the lateral surface bears two tall sharp-edged ridges, which extend across the length of the fragment, although their height decreases toward the posterior end. At the anterior end these ridges are closely spaced creating a deep, V-shaped sulcus between them. Toward the posterior end they diverge creating a broad, triangular fossa,” just like the dorsolateral groove in the *Saltriovenator* fragment (Fig. 5F). Moreover, “a thin, ventrally directed crest arises from the ventromedial margin. This creates a ventrolaterally facing, elongate fossa for the reception of the angular”: this is the ventrolateral groove seen in Fig. 5G.

### Tooth

A single tooth (MSNM V3659) was found isolated within a small limestone block near block A. Considering the uniqueness of the find, we confidently refer this tooth to the same taxon represented by the assemblage of bones. The specimen, missing the root and the apex, is 43 mm long and 18 mm wide (thus the tooth crown height is 2.4 times the base length). The crown is typically ziphodont: elongate, pointed, distally recurved and laterally compressed, without basal constriction, and with denticulate carinae (Figs. 5L–5N).

Following Hendrickx, Mateus & Araújo (2015a), with a crown height ratio of 2.39 and a crown base ratio of 0.48 (Table 1), the tooth referred to *Saltriovenator* can be considered moderately elongated (category range 1.5–2.5) and moderately narrow (category range 0.5–0.6). At closer examination, the apicobasal curvature of the distal margin of the crown in labial/lingual view can be defined as marked, because the apex of the tooth is placed distally to the distal margin of the crown base, the mesial margin is clearly convex and the distal margin is concave.

**Table 1:** Selected numbers and measurements (in mm) of *Saltriovenator zanellai*.

Skeletal element	Dimension measured	Value
Splenial	Length	(103)
	Mediolateral width (at dentary suture)	11
	Dorsoventral width	(64)
Prearticular	Length	(61)
	Mediolateral width	16
	Dorsoventral width	(34)
Tooth	Crown height (CH)	43.46
	Crown basal length (CBL)	18.15
	Crown basal width (CBW)	(8.85)
	Number of denticles per five mm (denticle density) on mesial carina	–
	Mesial carina, denticle basal length	–
	Number of denticles per five mm (denticle density) on distal carina	12
	Distal carina, denticle basal length (DBL)	0.40
	Crown base ratio (CBW/CBL)	(0.48)
	Crown height ratio (CH/CBL)	2.39
Cervical rib*	lcr midshaft craniocaudal diameter	9.3
	lcr midshaft mediolateral diameter	9.8
Dorsal ribs*	ldr4 midshaft craniocaudal diameter	18.4
	ldr4 midshaft mediolateral diameter	31.6
	ldr3 midshaft craniocaudal diameter	25.0
	ldr3 midshaft mediolateral diameter	15.1
	ldr2 midshaft craniocaudal diameter	15.3
	ldr2 midshaft mediolateral diameter	26.6
	ldr1+5 midshaft craniocaudal diameter	21.7
	ldr1+5 midshaft mediolateral diameter	13.5
	rdr3 midshaft craniocaudal diameter	28.2
	rdr3 midshaft mediolateral diameter	11.4
	rdr1+2 midshaft craniocaudal diameter	24.0
	rdr1+2 midshaft mediolateral diameter	17.5
	rdr6 midshaft craniocaudal diameter	26.7
	rdr6 midshaft mediolateral diameter	15.7
	rdr4 midshaft craniocaudal diameter	23.8
	rdr4 midshaft mediolateral diameter	9.4
	rdr5 midshaft craniocaudal diameter	18.7
	rdr5 midshaft mediolateral diameter	6.7
Scapula	Length	L (670)
	Minimum width (at neck)	L (110)
	Maximum width of the blade	L 135
	Mediolateral width (thickness) at neck	L 24
	Mediolateral width (thickness) of glenoid	R 68
	Dorsoventral width of glenoid	R 58
Coracoid	Distance between bicipital tubercle and infraglenoid buttress (at centre top)	38
	Mediolateral width (thickness) near supracoracoid nerve foramen	17
	Mediolateral width (thickness) of the medial margin	14
	Mediolateral width of glenoid (thickness at infraglenoid buttress)	62
	Craniocaudal width of glenoid	65
Scapulocoracoid	Glenoid angle between scapula and coracoid	110°
Furcula	Width (arms span)	[232]
	Midshaft maximum transverse diameter	17
	Midshaft minimum transverse diameter	12
	Angle between the two arms	140°
Sternal plate	Fragment length	(110)
	Fragment mediolateral width	(60)
	Fragment dorsoventral width (minimum thickness)	4.7
Humerus	Length, proximal condyle to lateral distal condyle	L – R 358
	Mediolateral width at level of deltopectoral crest	L 55 R 52
	Craniocaudal width at level of deltopectoral crest	L 93 R 90
	Midshaft width	L 50 R 49
	Distal mediolateral width	L – R [106]
	Distal craniocaudal width	L – R 49
	Length of deltopectoral crest	L 98 R 94
Carpal bone	Maximum (?mediolateral) width	(45)
	Minimum (?craniocaudal) width	[35]
	Proximodistal lenght (thickness)	[18]
Metacarpal II	Length	129
	Proximal mediolateral width	60
	Proximal dorsoventral width	46
	Midshaft mediolateral width	30
	Distal mediolateral width	56
	Distal dorsoventral width	41
Manual phalanx II-1	Length	[65]
	Proximal mediolateral width	35
	Proximal dorsoventral width	52
	Midshaft mediolateral width	24
	Distal mediolateral width	[28]
	Distal dorsoventral width	34
Manual phalanx III-1	Length	44
	Proximal mediolateral width	(33)
	Proximal dorsoventral width	(21)
	Midshaft mediolateral width	[18]
	Distal mediolateral width	22
	Distal dorsoventral width	17
Manual phalanx III-2	Length	41
	Proximal mediolateral width	22
	Proximal dorsoventral width	30
	Midshaft mediolateral width	17
	Distal mediolateral width	22
	Distal dorsoventral width	18
Manual phalanx III-3	Length	56
	Proximal mediolateral width	[17]
	Proximal dorsoventral width	25
	Midshaft mediolateral width	12
	Distal mediolateral width	[14]
	Distal dorsoventral width	[16]
Manual phalanx III-4	Length	(38)
	Proximal mediolateral width	10
	Proximal dorsoventral width	25
	Dorsoventral width at flexor tubercle	35
	Midshaft mediolateral width	6.3
	Midshaft dorsoventral width	23
Digit III	Overall length	200
Distal tarsal III	Craniocaudal length	64
	Mediolateral length	72
	Maximum proximodistal width	21
	Minimum proximodistal width	3
Distal tarsal IV	Craniocaudal length	84
	Mediolateral length	57
	Maximum proximodistal width	30
	Minimum proximodistal width at “neck”	19
Metatarsal II	Length	(257)
	Mediolateral width at midshaft	[32]
	Mediolateral width at proximal end	46
	Craniocaudal width at proximal end	77
Metatarsal III	Length	(200)
	Mediolateral width at midshaft	–
	Mediolateral width at proximal end	50
	Craniocaudal width at proximal end	99
Metatarsal IV	Length	(217)
	Mediolateral width at midshaft	34
Metatarsal V	Length of proximal fragment	(56)
	Maximum (craniocaudal) width at proximal end	25
	Minimum (mediolateral) width at proximal end	16

**Notes:**

Where not specified, height or width or diameter are taken perpendicular to the length.

Symbols and abbreviations: (), preserved; [], calculated; –, measurement not possible; *, see Fig. 2 for ribs abbreviations; L, left; R (and if not specified), right.

The transverse cross-section of the crown is intermediate between lenticular and D-shaped types (*sensu* Hendrickx, Mateus & Araújo, 2015a), being moderately compressed but asymmetrical: both mesial and distal carinae face linguomesially and linguodistally, respectively, but the distal edge is sharper than the mesial one, and the labial side of the crown is more convex that the lingual one. Approaching the carinae, the crown edges remain convex either on the labial or on the lingual side, different from the condition seen in salinon-shaped and parlinon-shaped teeth (*sensu* Hendrickx, Mateus & Araújo, 2015a): the concave areas seen in Fig. 5L near the carinae are due to diagenetic crushing.

As in most basal theropods, the enamel surface texture is smooth without any wrinkles, also adjacent to the carinae, even at higher magnification (Figs. 5M–5N), and any ornamentation—such as flutes, longitudinal grooves or ridges, transverse, or marginal undulations—is absent.

The denticles are completely lost along the mesial carina, which is deformed, crushed, and eroded; small denticles (12 per 5 mm, i.e., 2.5 per mm) are preserved in a short medio-apical tract (7.3 mm long) along the less damaged distal carina (Figs. 5K, 5M and 5N). Following the morphological terms standardized by Hendrickx, Mateus & Araújo (2015a), the preserved denticles are chisel-shaped, apicobasally subrectangular, perpendicular to the carina, and symmetrically convex in the outline of the external margin; the interdenticular space is deep and narrow, the interdenticular slit—when not altered by erosion—seems shallow and triangular, without a lamina joining two neighboring denticles, and there are no interdenticular sulci (blood grooves).

The moderately compressed D-shaped cross-section and the lingually-sided carinae suggest a mesiolateral position for this tooth. In other words, it might be one of the first maxillary teeth from the upper right arcade, or one of the transitional dentary teeth from the lower left arcade. Comparison with the dentition of Early-Middle Jurassic theropod taxa allows to exclude affinity of the Saltrio tooth to known coelophysoids, which so far possess much smaller crowns (CH <15 mm) with minute denticles on the distal carina (>30 denticles per five mm; Buckley, 2009; Hendrickx & Mateus, 2014). *Dilophosaurus* is definitely more similar in denticle density (13 per 5 mm—C. Dal Sasso, 2004, personal observation on UCMP 37303), which in its turn is reported to be similar in *Sinosaurus* and *Cryolophosaurus* (Xing, 2012). On the other hand, the teeth of abelisaurids are usually low and weakly recurved, have a slightly concave, straight or convex distal profile, and irregular non-oriented enamel texture, and megalosaurid teeth are characterized by centrally-positioned carinae on both mesial and lateral crowns (Hendrickx, Mateus & Araujo, 2015b).

Affinities with the Ceratosauridae cannot be excluded, as the eroded lingual side in our specimen does not allow to verify the presence of the “diagnostic longitudinal grooves” described by Madsen & Welles (2000); however, in *Saltriovenator* it is absent “a wide concave area centrally positioned on the labial side of the crown,” mentioned as typical of this clade by Hendrickx, Mateus & Araujo (2015b). Similarity to allosaurid and metriacanthosaurid crowns is in the crown proportions, as well as denticle count (12 per mm—C. Dal Sasso, 2004, personal observation on *Allosaurus* MOR 693), but they differ in having apparent transverse undulations.

### Axial skeleton

The axial skeleton of *Saltriovenator zanellai* is totally lost, except for a dozen of rib fragments, all coming from block A (Fig. 2). Small pieces of vertebral processes might be present among the indeterminate material.

**Ribs.** Based on the literature (Allain, 2005; Madsen, 1976; Madsen & Welles, 2000) and on mounted skeletons of *Allosaurus fragilis* (MSNM V435) and *Tyrannosaurus rex* (MSNM V3902), we tentatively refer four fragments to left dorsal ribs, and five fragments to right dorsal ribs (Figs. 5O–5Q). Our interpretation is based on the curvature of the preserved fragments, taking the keeled margin and the (usually laterodorsal) most flattened face as reference sides to orient the rib pieces, and assuming that the thicker cross-sections are proximal and the thinner-flatter ones are distal.

These fragments range from 28 to 18 mm in maximum diameter, and from 15 to 8 in minimum diameter, which is consistent with mid-distal shaft rib size in a theropod about 25% larger than the 6-m-long *Allosaurus fragilis* MSNM V435. The bulkiest rib fragment (30 mm in diameter) has a subtriangular cross-section, a concavo-convex caudal side, a cranial ridge and a robust tapering keel projected medially. By comparison with the cross-section of a *Ceratosaurus* rib figured by Madsen & Welles (2000: plate 19) and by direct comparison with MSNM V435 we refer this fragment to the proximal portion of a left dorsal rib.

A tiny fragment with similar cross-section, less than 10 mm in diameter and preserving a very sharp medial keel, emphasized by a deep groove running caudomedially along its base, likely belongs to the midshaft of a left cervical rib.

The preserved ribs do not show any pneumatic recess. Four very fragmentary rib pieces remain indeterminate.

### Scapular girdle and forelimbs

This is the most represented portion of the appendicular skeleton of *Saltriovenator zanellai*, including the best preserved and most complete elements (right humerus, right manus). The bones of the scapular girdle and the two humeri come all from block A; the bones of the right manus and part of the left humerus have been extracted from block B.

**Scapula.** A total of 15 fragments of the left scapula have been recovered from block A (Fig. 2), and patiently reconnected into three main portions (Figs. 6A–6D). Although the broken edges of the three portions are not complementary, they can be referred to adjacent parts of the same bone thanks to similar size and craniocaudal diameter, flattened structure with continuous longitudinal ridged texture and continuous mediolateral curvature, lenticular cross-section with same cortical bone lamination and thickness, and macro-vacuolar aspect of the inner spongy bone. In addition, the presence of a longitudinal keel along a tapering thinner cranial edge, and of a thicker crest along a robust caudal edge, in all the three portions, allowed to orient them correctly (e.g., see the elongate drop-like cross-section in Madsen & Welles, 2000: p. 20). Reconstructed this way, the scapula of *Saltriovenator* results approximately two times longer than the humerus.

The distalmost portion of the left scapular blade is distinguished by its thinner cross-section, dorsally tapering in cranial and caudal view (Figs. 6B and 6D), dorsally diverging margins (Figs. 6A and 6C), and equally diverging surface texture. Possibly, five other small fragments showing similar flattening and texture (Fig. 2, sc label) are part of the same bone, or of the counterlateral element.

The costal (medial) surface of the scapular blade of *Saltriovenator* is flat. Only vascular pits and tracks, running on the medial surface of the acromion, are present. Below the neck, the scapula becomes much thicker (50 mm) and stouter along the caudal margin, whereas in cranial direction it tapers into the acromion. Only part of it is preserved in our specimen, with an axe-shaped fragment, that is, concave medially and convex laterally. Due to breakage, it is impossible to know how much was the acromion prominent, and if the scapulocoracoid was notched between acromion and coracoid (e.g., as it is in *Dilophosaurus*, unlike *Ceratosaurus*). On the lateral side of the scapula, a wide fossa proximal to the acromion and opposite to its medial concavity marks a powerful muscle attachment site, likely for the *M. supracoracoideus* (Burch, 2017). The maximum mediolateral diameter of the scapula (70 mm) is reached in the fragment that bears the glenoid face. The latter is intact, with an elongate D-shaped profile and a perfect line of contact (scapulocoracoid suture) with the glenoid of the right coracoid (Figs. 6E–6H), which fossilized close to it and to the right humerus (Fig. 2C). Given this, we refer the preserved scapular glenoid to the right scapula, albeit the fragments of the left scapula are by far most abundant. The well-preserved scapulocoracoid suture allows to restore the glenoid cavity, which appears directed mainly caudoventrally, without lateral exposition, as in basal neotheropods (Rauhut & Pol, 2017). The resulting glenoid angle, seen in lateral view, is a broad arc that measures about 110° (Fig. 6G). The scapular glenoid is wider (mediolaterally) than long (dorsoventrally), measuring 68 × 58 mm; its participation to the glenoid cavity is approximately equal to that of the coracoid. In medial and lateral view, the scapular glenoid shows a distinct outer lip that points caudally.

In the type specimen of *Dilophosaurus wetherilli* (C. Dal Sasso, 2004, personal observation on UCMP 37302) the scapular glenoid is squared rather than D-shaped, the angle formed by the glenoid with the articular surface for the coracoid is identical (140°), the glenoid angle is more open (125°), and the supraglenoid lip in lateral view is slightly more pronounced, almost hook-like, as in *Ceratosaurus* (Madsen & Welles, 2000) and *Majungaurus* (Carrano, 2007). In a subadult specimen of *Allosaurus fragilis* (C. Dal Sasso, 2004, personal observation on MOR 693) the scapular glenoid is subrectangular and much smaller than in *Saltriovenator* (49 mm long × 38 mm wide), as it is the coracoid glenoid (39 mm long × 40 mm wide), and they form a glenoid angle of 105°. In *A. fragilis* the scapula is dramatically narrower and more slender than in *Saltriovenator*, bladelike, with a dramatic proportional reduction of the coracoid. On the other hand, the scapula of *Dilophosaurus wetherilli* (Welles, 1984: fig. 25) has a subrectangular distal expansion, and a shaft with concave cranial and caudal edges.

Using the best preserved holotypic right scapula of *Dilophosaurus* to track a scaled reference silhouette in a tentative recomposition of the scapula of *Saltriovenator*, the latter fits a narrower, feebly cranially curved profile, without remarkable distal expansion: three important differences that make the scapula of *Saltriovenator* definitely more similar to those of *Ceratosaurus dentisulcatus* (Madsen & Welles, 2000: p. 20) and, secondarily, *Eoabelisaurus* (Pol & Rauhut, 2012).

**Coracoid.** A large thick, concavo-convex bone fragment (Figs. 6E–6J) is identified as the caudodorsal portion of the right coracoid, thanks to the preservation of the supracoracoid nerve foramen, the bicipital (also named lateral or coracoid) tubercle, the infraglenoid buttress, and the characteristic fossa than runs between these two prominent processes. As in several theropods, the bicipital tubercle is developed as a boss-like prominence: in some tetanurans, including *Allosaurus* (Madsen, 1976), this tubercle is extended along the lateral surface of the bone, forming a distinct ridge, but in *Saltriovenator* it is more prominent and forms a very elongate triangle, which is remiscent of the condition seen in several basal neotheropods, such as *Coelophysis rhodesiensis* (Raath, 1977), *Zupaysaurus* (Ezcurra & Cuny, 2007), and *Dilophosaurus* (see below), and different from the low rigde seen in *Ceratosaurus* (Madsen & Welles, 2000). The infraglenoid buttress seems taller and more pointed than the bicipital tubercle, but the latter is eroded, and the similar basal transverse diameter suggests that they were subequal in size, like in *Dilophosaurus wetherilli* (C. Dal Sasso, 2004, personal observation on UCMP 37302). In addition, in *Saltriovenator* the fossa is asymmetrical in the same way, with the bicipital side, which is subvertical, and the infraglenoid side oblique. In *Allosaurus* the infraglenoid-bicipital complex is much less pronounced, either in juvenile or adult specimens (C. Dal Sasso, 2004, personal observation on a growth series on loan to MOR from UUVP).

The coracoid of *Saltriovenator* lacks a lipped margin of the glenoid: the infraglenoid buttress forms a lip but it is directed laterally, not invading the glenoid margin. The supracoracoid nerve foramen continues in a groove, which is directed craniodorsally (dorsally in *Sinosaurus—*Hu, 1993; *Ceratosaurus dentisulcatus—*Madsen & Welles, 2000; *Majungasaurus*—Carrano, 2007), and still wide open at the broken end of the fragment. On the other hand, in *Dilophosaurus wetherilli* the supracoracoid nerve foramen widens in cranial direction, and with a more open angle, and does not show any groove or fossa (C. Dal Sasso, 2004, personal observation on UCMP 37302).

On the caudodorsal side of the bone, the coracoid glenoid is preserved as a smooth concave area, about 65 mm long (dorsoventrally) and 62 mm wide (mediolaterally). Laterally the glenoid is bordered by a rim, which extends in cranial direction from the infraglenoid buttress, and medially it becomes unclear because the bone cortex is missing. In facts, the nutrient foramen of the glenoid is widened by this lack of bone. The scapular face is deep and robust, remarkably similar to the “extremely thick contact with the scapula” described in *Sinosaurus* (Hu, 1993), also present in *D. wetherilli* (C. Dal Sasso, 2004, personal observation on UCMP 37302) and *Segisaurus halli* (Carrano, Hutchinson & Sampson, 2005: fig. 5).

The main coracoid fragment of *Saltriovenator* represents about one-third of the whole bone and preserves a good portion of the caudoventral margin, as shown by the ridge that borders the medial concavity. A second ridge marks the dorsomedial edge of the scapulocoracoid suture.

Four smaller bone pieces are referred to the flattened, fan-like portion of the coracoid as they show similar texture (fine parallel ridges), cross-section (concavo-convex bone, with one rounded margin), thickness (10–15 mm), and structure (thin-walled and finely spongy bone). Two of these fragments (rco2 and rco3 in Figs. 5E–5G) are likely the ventral continuation of the largest portion the right coracoid, as they were found overlapped onto it (Fig. 2B) and almost match each other along their fracture lines; the other ones, being thinner, are tentatively positioned more cranially (and might also belong to the left coracoid).

Based on preserved parts, the reconstructed coracoid appears proportionally smaller than expected from the size of the scapula, if compared to *Dilophosaurus*; the disproportion is minor in *Ceratosaurus* and *Eoabelisaurus*, and is the opposite in *Allosaurus*, due to its quite elongated scapula. Moreover, the coracoid of *Saltriovenator* is much longer parallel to the scapular suture than perpendicularly to it, and deep dorsoventrally (see depth of scapular facet in Table S1).

The caudoventral margin of the coracoid in *Saltriovenator* is gently rounded and lacks either a long pointed (e.g., *Allosaurus*) or distinctly hooked (e.g., *Limusaurus*) process, usually bound proximally by the infraglenoid buttress, which is present in *Elaphrosaurus* (Rauhut & Carrano, 2016), abelisaurids, and many averostrans, but not in *Ceratosaurus* (Rauhut, 2003). In facts, in *Ceratosaurus* the caudoventral margin is similarly curved, “short and bluntly rounded” (Tykoski & Rowe, 2004). In this aspect, the highest affinity is with the type specimens of *Dilophosaurus wetherilli* (C. Dal Sasso, 2004, personal observation on UCMP 37302) and *Cryolophosaurus ellioti* (Smith, Hammer & Makovicky, 2017; P. Makovicky, 2017, personal communication on FMNH PR 1821), both having the caudal margin of the coracoid regularly rounded with the same arch span. This suggests that also *Saltriovenator* had subelliptical rather than suboval coracoids, that is, it retained a rather plesiomorphic morphology, shared among basal saurischians.

**Furcula.** The furcula (Figs. 6K–6O) was extracted during acid treatment of block A, in close association to all other elements of the pectoral girdle (Fig. 2A). This bone cannot be misinterpreted as a gastral basket element because the two rami are stout, lack any longitudinal groove, and are medioventrally united in a clearly defined hypocleideum; furthermore, the complete right ramus terminates with a flat epicleideal facet (or epicleideum), which is typically spatulate and sulcated by ligamental scars, for articulation with the scapular acromion (Chure & Madsen, 1996; Carrano, Hutchinson & Sampson, 2005).

In the last two decades, furculae have been documented in nearly all but the most basal theropods (such as *Herrerasaurus*). The discovery of furculae in coelophysoids (Tykoski et al., 2002) has ruled out previous hypotheses on the phylogenetic position of *Saltriovenator* (Dal Sasso, 2001b), which were based on the idea that the fusion of the two clavicles occurred only in the Tetanurae. At present, the oldest known furculae belong to *Coelophysis bauri* and date back to the Late Triassic (Rinehart, Lucas & Hunt, 2007).

The furcula of *Saltriovenator* is V-shaped in ventral view (Fig. 6N) and U-shaped in cranial view (Fig. 6K) because, toward the symphysis, the dorsal margins of the two rami are concave, rather than straight. The preserved epicleideum is definitely twisted craniolaterally and expanded dorsoventrally at midlength of the facet, then it tapers to a pointed end. In cross-section (Fig. 6N), the two rami of the furcula are D-shaped in proximity to the symphysis, with the flat side facing dorsally; distally, the convex side develops a longitudinal ridge that eventually becomes the ventral edge of the epicleideum. The cross-section of the epicleideum is like a compressed D, with the flat side facing cranially. The hypocleideum of *Saltriovenator* projects caudoventrally seven to eight mm from the base of the clavicular rami, pointing to the left with a slight asymmetry. Interestingly, basal neotheropods such as *Segisaurus* and *Dracoraptor* lack (Carrano, Hutchinson & Sampson, 2005; Martill et al., 2016) or do not show (Chure & Madsen, 1996; Makovicky & Currie, 1998; Tykoski et al., 2002) prominent hypocleidea.

As commonly observed (Carrano, Hutchinson & Sampson, 2005), there is no trace of interclavicular suture between the two rami, which indicates a complete fusion. This was confirmed by CT analysis, which also excluded the presence of pneumatic openings and internal pneumatisation (Sereno et al., 2008), not to be confused with the wide medullary cavities visible especially inside the two rami.

With the method of measurement used by Nesbitt et al. (2009) we estimate an interclavicular angle of 140° for *Saltriovenator zanellai.* In coelophysoids, the furcula is variably U- or V-shaped and has an angle of 115–140°; the furcula is V-shaped also in allosauroids and ranges from 120° to 135° (Nesbitt et al., 2009). A “widely arched” furcula is present in *Limusaurus* (Xu et al., 2009).

**Sternum.** Remains of sternal plates were present in block A, partially mixed with other flat bone fragments of scapula and coracoid (Fig. 2B). In particular, we reconnected two fragments into a platelike, weakly curved bone margin (Figs. 5P–5R), which at first sight we hypothesized to be the distal end of the scapular blade, but eventually could not fit that position. This element shows a carinate (keeled) margin which is thinner than the thinnest preserved margin of the scapular blade, a similar spongy interior, but a different surface texture, which in facts is randomly oriented and finely pitted, well-vascularized, and it lacks the fine parallel striae that run all along the scapula. A couple of smaller fragments were recovered piled up on the former (Fig. 2A, st label) and share very similar shape and ornamentation. These features are also visible in the sternal plates MPG-KPC1 and 2, described by Sánchez-Hernández & Benton (2014: fig. 10) in *Camarillasaurus cirugedae*, a Cretaceous ceratosaurian from Spain. By comparison with the latter specimens, which are by far more complete, we suggest that our fragment may represent the caudolateral corner of the right sternal plate, and approximately one-eighth of the whole bone (Fig. 10B).

This would be the fourth time that sternal plates have been described in a ceratosaurian theropod, after *Carnotaurus* (Bonaparte, Novas & Coria, 1990), *Limusaurus* (Xu et al., 2009), and *Camarillasaurus* (Sánchez-Hernández & Benton, 2014).

**Humerus.** The humeri are the largest bones and the only paired elements known from both sides of *Saltriovenator zanellai* (excluding the clavicles that are fused into a furcula). The right humerus (Figs. 7A–7F) is by far more complete as it lacks only part of its head, and the adductor crest (=internal tuberosity of Madsen, 1976); the left humerus (Figs. 7G–7L) lacks not only the adductor crest, the extensor crest and part of the proximal diaphysis, but also the whole distal half. In both humeri, mainly on the fossae for the *M. coracobrachialis*, apparent subcircular marks are present; as written above (taphonomical section), these marks represent post-mortem damage (macroborings produced by marine invertebrates). The midshaft cut of the left humerus shows a wide open internal hollow, which occupies more than half of the diameter of the bone; CT analyses of the right humerus and right metatarsal II show that this relationship between cortex and medulla is present in the whole bone, even more marked towards and inside the epiphyses, as expected in the long bones of a theropod dinosaur.

The shaft torsion of the humerus of *Saltriovenator*, measured as the angle between the trasverse axes of proximal and distal ends when viewed proximally/distally, is about 74°. The main axis of the head is oriented transversally and collinear with the plane of the proximal expansion of the humerus, thus differing from some tetanurans (Benson & Xu, 2008) in which it forms a distinct acute angle with the main transversal plane of the proximal end. In proximal view, the head of the left humerus is more complete than the right one and appears ellipsoidal, expanded more lateromedially than proximodistally, that is, not inflated or dome-shaped, far from the globular shape seen in noasaurids and abelisaurids: thus remarkably plesiomorphic, as in *Eoabelisaurus* (Pol & Rauhut, 2012), and contrary to *Limusaurus* (Xu et al., 2009). The lateral tuberosity of the humerus is placed laterodistally to the head, at the level of the proximal end of the deltopectoral crest. It is well-developed, giving the lateral margin a straight profile in cranial and caudal view; in late-diverging ceratosaurians it is reduced, giving the humerus a broadly convex margin. The right humerus of *Saltriovenator* appears almost straight also in lateral and medial view, being just slightly bent in its distal third. Likely, the missing adductor crest was as slightly curved as the distal epiphysis, giving the whole bone a only moderately sigmoid shape, as in most Neoceratosauria (Tykoski & Rowe, 2004), and in some large-sized basal tetanuran taxa, such as *Poekilopleuron* (Allain & Chure, 2002; C. Dal Sasso & S. Maganuco, 2004, personal observation on plastotype MNHN 1897-2), *Acrocanthosaurus, Szechuanosaurus* (Gao, 1993), and *Xuanhanosaurus* (Novas, Aranciaga Rolando & Agnolín, 2016). It is also similar to *Dilophosaurus* (Welles, 1984), although in the latter the diaphysis is more slender and a little more bowed, with an arch which is continuous from the dorsal lamina to the entepicondylar crest (C. Dal Sasso, 2004, personal observation on UCMP 37302). In coelophysoids, the humerus shows a clearly sigmoid curvature, as well as torsion (Tykoski & Rowe, 2004). In *Allosaurus* (Madsen, 1976; C. Dal Sasso, 2004, personal observation on MOR 693) the humerus is markedly sigmoid, the diaphysis in craniocaudal view is narrow and bowed medially, and there is an increased torsion of the epiphyses, which are proportionally more enlarged.

The deltopectoral crest is the largest process of the humerus of *Saltriovenator*: proximally, it is not confluent with the humeral head, being separated from it by a shallow concavity that houses a thin extensor crest, like in *Allosaurus* (Madsen, 1976) and unlike *Dilophosaurus* (Welles, 1984). Distally, the deltopectoral crest becomes transversely inflated, and—remarkably and uniquely—it protrudes craniomedially for more than twice the midshaft diameter size, finally meeting the distal lamina abruptly, with an angle of 90°. The deltopectoral crest of *Saltriovenator* forms an angle of 50° with the plane of the distal condyles and it extends for more than 2/5 the humeral length, as in *Dilophosaurus wetherilli* (C. Dal Sasso, 2004, personal observation on UCMP 37303) and contrary to most tetanuran theropods, in which it extends in cranial direction. On the left humerus, the protruding end of the deltopectoral crest is much more pointed than in the right humerus, nearly hooked, being grown over the distal lamina—in this feature, it recalls *Acrocanthosaurus* (Currie & Carpenter, 2000). This condition, as well as the right-angled distal end, differs from the more gentle transition between the crest and the shaft seen in most theropods, including *Ceratosaurus* (Madsen & Welles, 2000) and *Dilophosaurus* (C. Dal Sasso, 2004, personal observation on UCMP 37303), and other taxa that possess a similarly protruding deltopectoral crest, such as *Poekilopleuron* (Allain & Chure, 2002), *Szechuanosaurus* (Gao, 1993), *Torvosaurus* (Galton & Jensen, 1979), and *Australovenator* (Novas, Aranciaga Rolando & Agnolín, 2016). A nearly perpendicular distal lamina of the deltopectoral crest can be seen only in *Segisaurus* (Carrano, Hutchinson & Sampson, 2005).

The proximodistal length of the remaining humeral shaft, between the deltopectoral crest and the distal condyles, is about five times the minimal shaft diameter. In this portion, the shaft does not bear any distinct tuber along the craniolateral surface, whereas on the caudolateral margin, at level of the distal lamina, an elongate scar for the *M. latissimus dorsi* is present, like in *Majungasaurus* (Carrano, 2007: fig. 3).

In cranial view, the humerus of *Saltriovenator* appears non-sigmoid, almost straight, similar to the holotype of *Ceratosaurus dentisulcatus* (Madsen & Welles, 2000: fig. B, D) but a little less bulky, with less pronounced, gently enlarged epiphyses; therefore it markedly differs from the midshaft-constricted holotype of *C. magnicornis* (Madsen & Welles, 2000: fig. A, C). In facts, in *Saltriovenator* the distally placed distal condyles are slightly less than twice larger than the diaphysis at its minimum transverse diameter.

In distal view, the partially eroded (or not completely ossified) condyles are only weakly convex (nor hemispherical, neither totally flattened) and subequal in size, the ectocondyle being slightly shorter in mediolateral direction, but deeper craniocaudally. The same condition is observed in *Dilophosaurus wetherilli* (C. Dal Sasso, 2004, personal observation on UCMP 37303) and *Cryolophosaurus ellioti* (Smith et al., 2007: fig. 14 C-D). The intercondylar sulcus is preserved only at its ends, it is shallower than in *Dilophosaurus*, mediolaterally narrow and slit-like in shape. The distal fossa is moderately developed. Although not hypertrophied, the ectepicondylar crest seems more developed than in *Dilophosaurus* and than the entepicondylar crest, but this may be an artifact of preservation, because the medial wall of the entocondyle is missing. In *Allosaurus* (Madsen, 1976; C. Dal Sasso, 2004, personal observation on MOR 693) the disproportions between the ecto- and the entocondyle increase, the latter becoming almost twice than the former in mediolateral length and much more compressed craniocaudally; the intercondylar groove markedly divides the two condyles and the ectepicondylar crest appears as robust as in *Saltriovenator*.

#### Manus: carpus and metacarpus

The preserved manual elements of *Saltriovenator* are one carpal, the right second metacarpal, the first phalanx and part of the second phalanx of the same finger, four phalanges that perfectly articulate each other when connected and are referred to the third finger, and the tip on an indeterminate ungual phalanx (Figs. 8–10 and 12). These bones were closely associated, although not in articulation, in the same limestone block, together with the right second metatarsal (Figs. 3H–3J).

The manual morphology of *Saltriovenator* demonstrates to be more similar to that of basal ceratosaurians (*Ceratosaurus*, *Eoabelisaurus*), and secondarily to that of dilophosaurids (*Dilophosaurus*) and allosauroids (*Allosaurus*, *Sinraptor*), than to the condition present in either late-diverging ceratosaurians (*Limusaurus,* abelisaurids), or in coelurosaurians. Interestingly, when rearticulated in neutral (straight) pose, the manual elements of both the second and third digit of *Saltriovenator* result in slightly side-bended fingers (Fig. 10C).

**Carpal bone.** At first sight this element (Figs. 13M–13R) appears as a little portion of a much larger bone, with a convex side slightly constricted medially, which recalls the articular condyles of a long bone. Actually, on the very eroded opposite side a small area of finished bone cortex is preserved, with a surface which is flat and parallels the other side, thus indicating that this bone was small and disk-like. Based on such shape, this element likely pertains to the carpus of *Saltriovenator*. No carpals have been found in the articulated hands of *Ceratosaurus*, *Limusaurus*, *Majungasaurus*, and *Aucasaurus*. Moreover, in *Ceratosaurus* USNM 4735 the whole arm is preserved in articulation, showing an apparent gap between the forearm and the metacarpals that indicates a non-ossified area (Carrano & Choiniere, 2016), as it is the case in *Limusaurus*. However, a large carpal 1 + 2 has been reported in a yet undescribed noasaurid from Niger (Sereno, Wilson & Conrad, 2004), a flattened discoidal bone was found associated with the proximal ends of metacarpals III and IV in *Dilophosaurus* (Welles, 1984), and only a single carpal is preserved in *Eoabelisaurus* (Pol & Rauhut, 2012), supporting the idea that *Saltriovenator* might have had a single carpal too. As it articulates to the metacarpal II, we consider the *Saltriovenator*’s carpal homologous to carpal 2 (Fig. 10B).

In sum, none of the known ceratosaurians has more than one ossified carpal bone: this leads us to suspect that most ceratosaurians had non-ossified, cartilaginous carpals. The presence of a single ossified carpal in *Saltriovenator* and *Eoabelisaurus* would represent an intermediate condition, before the complete loss of ossified carpals that can be observed in *Limusaurus* and in the Cretaceous ceratosaurians.

**Metacarpal II.** This bone from the right manus is almost complete and remarkably well-preserved (Figs. 8A–8F). Although metacarpal III was not recovered, its relationship with metacarpal II can be inferred from the anatomy of the latter: the distal end of metacarpal II unequivocally shows no evidence of a distal fusion with metacarpal III, and the contact for metacarpal III is limited to the lateral margin of the proximal portion of metacarpal lI. The same condition can be inferred for metacarpal I, whose contact projects proximomedially as a subrectangular facet at the apex of a low buttress, unlike tetanurans, and comparable to *Ceratosaurus* (Carrano & Choiniere, 2016: fig. 7), *Dilophosaurus* (Welles, 1984), and *Eoabelisaurus* (Pol & Rauhut, 2012). This condition was likely present—albeit not surely—also in *Berberosaurus* (Allain et al., 2007), pending confirmation (Carrano & Choiniere, 2016) that the preserved bone of *Berberosaurus* might instead represent a left metacarpal III (by affinity with the third metacarpal of *Ceratosaurus*: note that this interpretation is followed in the character score of the phylogenetic analysis used here).

The metacarpal II of *Saltriovenator* is peculiar in having dramatically enlarged and robust articulations, which give the bone an hourglass shape, especially in dorsal and palmar views, where the midshaft diameter reaches half the proximal transverse diameter; in lateral and medial views the shaft shows parallel margins, and its palmar side is flat, like in *Ceratosaurus* (Carrano & Choiniere, 2016), so that in cross-section it results subrectangular. As in *Berberosaurus* and *Ceratosaurus*, but not in *Limusaurus* (Xu et al., 2009), the proximal end is wider than the distal, and greatly expanded from the shaft. In this respect, as well as in size, the bone of *Saltriovenator* is more similar to that of an adult *Allosaurus* (Madsen, 1976) or *Sinraptor* (Currie & Zhao, 1993), than *Dilophosaurus* and coelophysoids (Welles, 1984), in which it is by far much more slender, with gently concave diaphyseal margins and moderately expanded epiphyses. The overall morphology of this bone suggests the presence of powerful manual muscles in *Saltriovenator*.

Although the dorsomedial process of the proximal articulation is missing, the original subtrapezoidal shape of the bone is still evident in proximal view, where the dorsal, palmar, and lateral sides are concave and three prominent bony processes, representing the contact limits for the adjacent metacarpals, mark the preserved articular apices. These processes continue in form of robust longitudinal ridges along the diaphysis, up to the midshaft. As in *Ceratosaurus* (Carrano & Choiniere, 2016), but less than in *Berberosaurus* (Allain et al., 2007), the most pronounced proximal concavity of *Saltriovenator* is the broad and triangular palmar flexor fossa, which occupies almost the entire width of the bone until the midshaft, and the second wide concavity is the dorsal extensor fossa. The similarly trapezoidal proximal articulation of *Allosaurus* (C. Dal Sasso, 2004, personal observation on MOR 693) differs in having a more excavated and more ventrally-facing contact for metacarpal III, a well-delimited articular facet for a carpal bone on the medioventral margin, and a depression for a second carpal on the proximolateral corner (both absent in *Saltriovenator*). In *Dilophosaurus* (Welles, 1984) the proximal end is also trapezoidal but less bulky, being compressed in dorsopalmar direction, having less pronounced dorsal and palmar concavities, and proximal ridges shorter and less prominent. In *Saltriovenator* the lateral margin of the proximal end meets the dorsal margin at an angle of about 80°, just like in *Ceratosaurus* (Carrano & Choiniere, 2016: fig.7E), forming a tab that, with metacarpal III in articulation, overlaps its proximomedial portion (Fig. 10C). A similar condition is also present in *Dilophosaurus* (Xu et al., 2009: fig. 2c; C. Dal Sasso, 2004, personal observation on UCMP 37302 and UCMP 37303).

In *Saltriovenator* the stout distal epiphysis terminates in a ginglymoid articulation which is asymmetrically and obliquely partitioned by the intercondylar sulcus into two condyles: a medial condyle, placed more proximally but deeper palmodorsally, centrally concave and with sharp medial edge; and a lateral condyle, more extended distally than the medial condyle, with convex (hemispherical) articulation and with rounded lateral edge. The same asymmetry and rotation of the distal condyles relative to the long axis of the bone (around 40°) is present in *Berberosaurus* (Allain et al., 2007) and *Ceratosaurus* (Carrano & Choiniere, 2016), and with minor degree (about 30°) in *Dilophosaurus* (Welles, 1984), *Eoabelisaurus* (Pol & Rauhut, 2012), *Limusaurus* (Xu et al., 2009), and some specimens of *Coelophysis* (Galton, 1971). It is absent in late-diverging abelisauroids as well as in most other theropods, including the tetanurans *Allosaurus* (Madsen, 1976) and *Sinraptor* (Currie & Zhao, 1993), which possess subequal, subparallel, and subvertical distal condyles, divided by a deeper intercondylar sulcus. In *Saltriovenator* both condyles are side-marked by well-developed fossae and pits for the collateral ligaments and extend in dorsal aspect, where the lateral condyle occupies almost two-thirds of the distal articulation. In lateral and medial view, the condylar surfaces further extend to the palmar side, tracing a semicircular excursion (as in *Ceratosaurus*) rather than three quarters of it (e.g., *Dilophosaurus*, *Allosaurus*); the medial condyle terminates in a pronounced “lip-like” projection directed proximopalmarly, a feature found only in *Berberosaurus* (Allain et al., 2007). In palmar view, the two condyles are divided by a shallow fossa for the flexor ligament. Continuous with the dorsal end of the intercondylar sulcus, a deep extensor ligament pit opens, bordered by an enlarged semicircular lip of bone that protrudes dorsolaterally over the condylar level. This lip delimits the dorsal excursion of the distal articular complex, functionally acting as a stop for the maximum extension/supination of the first phalanx of digit II (see below). The pit-and-lip complex is dramatically developed in *Saltriovenator*: more than in any other theropod, including *Berberosaurus* (Allain et al., 2007), *Ceratosaurus* (C. Dal Sasso & S. Maganuco, 2014, personal observation on USNM 4735), *Eoabelisaurus* (Pol & Rauhut, 2012), *Sinraptor* (Currie & Zhao, 1993), and *Dilophosaurus.* In the latter, the extensor ligament pit is not well-figured (Welles, 1984: fig. 37) and is described as six mm deep. Under direct observation (C. Dal Sasso, 2004, personal observation on UCMP 37302 and 37303), this pit results proportionally shallower and mediolaterally narrower than in *Saltriovenator*, and the lip reaches but not oversizes the dorsal limit of the condyles. On the other hand, in *Sinraptor* (Currie & Zhao, 1993: fig. 20) and *Allosaurus* (Madsen, 1976: pp. 43–45) the pit is wide and subcircular, extended towards the bone midshaft, and the lip is lower that the dorsal end of the distal condyles (*Sinraptor*) or absent (*Allosaurus*). Interestingly, the tetanurans *Acrocanthosaurus* (Currie & Carpenter, 2000), *Szechuanosaurus zigongensis* (Gao, 1993), *Xuanhanosaurus* (Dong, 1984), and *Torvosaurus* (Galton & Jensen, 1979) lack a protruding lip but retain a deep extensor ligament pit and asymmetrical distal condyles.

#### Manual phalanges

The manual phalanges of *Saltriovenator* share a series of features that are phylogenetically informative (see below): the ventral processes of the proximal ends are prominent and mediolaterally expanded; in no digit the diaphysis of one bone is shorter than its distal epiphysis (the opposite condition is seen in the abelisaurids); the distal epiphysis of the non-ungual phalanges has well-defined condyles that are asymmetrical in the proximal phalanges, with the lateral condyle projecting distally more than the medial one. In addition, with the exception of phalanx II-1, the collateral ligament pits are present but shallow, and weakly developed.

**Phalanx II-1.** The first phalanx of the right second manual digit lacks the palmar half of the distal end, due to a longitudinal oblique cut (Figs. 8G–8L). It is dramatically short and bulky: the proximodistal length is subequal to the 5/2 of the mediolateral width at mid-shaft, and the bone tapers in diameter rapidly as it extends distally, like in *Ceratosaurus,* but in proximal view and in transverse cross-section it differs from the latter in being subrectangular, taller than wide, rather than quadrangular.

In lateral view, albeit the shaft of this phalanx is deeper than that of its metacarpal, the articular facets of the two bones result definitely complementary, making a perfect gynglimoid joint. On the contrary, in dorsal and palmar view phalanx II-1 seems too narrow for such stout metacarpal II, but this condition is not uncommon among ceratosaurian theropods. For instance, in *Ceratosaurus* (Madsen & Welles, 2000; Carrano & Choiniere, 2016) and *Eoabelisaurus* (Pol & Rauhut, 2012) the two bones have very similar relative proportions, including the “unusually narrower” first phalanx.

In *Saltriovenator* the proximal facet is saddle-shaped, divided by a dorsopalmar ridge located on its medial third. The division is oblique and unequal, just like in phalanx II-1 of *Ceratosaurus* (Carrano & Choiniere, 2016: fig.10) and unlike the one of *Dilophosaurus* (Welles, 1984): mirroring the distal condyles of the second metacarpal, the lateral articular portion is almost twice wider than the medial one, and much more excavated. On the other hand, the palmar flexor groove runs along the midline of the bone, making the medial flexor process and the lateral flexor process equally developed in proximal view. Actually, in palmar and lateral views the lateral flexor process reveals to be shorter: as in *Ceratosaurus* (Carrano & Choiniere, 2016), it does not extend as far proximally, projecting laterally as a smaller bulbous protuberance, with a rugose attachment area that terminates in a well-marked lateral sulcus. A couple of nutrient foramina opens along the palmar flexor groove of *Saltriovenator*, which is deep and mediolaterally narrow, similar to that of *Ceratosaurus, Eoabelisaurus* (Pol & Rauhut, 2012), and *Aucasaurus* (Carrano & Choiniere, 2016). In the type specimen of *Dilophosaurus wetherilli* (C. Dal Sasso, 2014, personal observation on UCMP 37302) the lateral sulcus is absent, the medial flexor process is less developed, and the palmar flexor groove much less excavated. In *Allosaurus* (Madsen, 1976; C. Dal Sasso, 2004, personal observation on MOR 693), II-1 in proximal view is subpentagonal to triangular, narrowing in the dorsal half; the division of the articular facet is subequal, with the medial portion slightly narrower than the lateral, the palmar flexor groove is wide, shallow and regularly concave, and the lateral sulcus is present.

Unlike *Ceratosaurus* (Carrano & Choiniere, 2016: fig.10; C. Dal Sasso & S. Maganuco, 2014, personal observation on USNM 4735), distal to the dorsal extensor process, the phalanx II-2 of *Saltriovenator* displays a bump, not a fossa. Such mid-dorsal protuberance is also absent in other theropods, being not even homologous to the “distinct lateral process proximodorsally” present in *Limusaurus* (Xu et al., 2009).

The incomplete distal condyles seem subequally developed and slightly rotated counterclockwise, but quite less than in the metacarpal (about 20–25°) relative to the long axis of the proximal epiphysis. A shallow and wide extensor ligament pit is present above the distal articulation and is reached by the intercondylar sulcus; an equally wide pit for collateral ligaments flanks the lateral condyle.

In *Saltriovenator*, phalanx II-1 measures less than half the length of metacarpal II; in *Dilophosaurus* (Welles, 1984), it is 70% the length of the metacarpal and quite slender, not at all bulky. In tetanuran theropods such as *Allosaurus* (Madsen, 1976) and *Sinraptor* (Currie & Zhao, 1993) the two elements are more similar both in transverse diameter and length (no less than 80% of metacarpal II). However, interestingly, some basal tetanurans that retain a vestigial fourth metacarpal, such as *Szechuanosaurus* (Gao, 1993) and *Xuanhanosaurus* (Dong, 1984), also retain ceratosaurian-like short phalanges II-1, which are much shorter than in *Dilophosaurus* and approach *Saltriovenator* (40–45% of metacarpal II in length). This, coupled with morphological affinities from other skeletal elements (e.g., humerus, second metacarpal), gives support to the hypothesis that these taxa belong to early-diverging branches of the ceratosaurian-tetanuran node (Averostra) and share appendicular symplesiomorphies of this clade, features then modified or lost in late-diverging members of both Ceratosauria and Tetanurae (see Discussion, below).

**Phalanx III-1.** This fairly complete element, dorsoventrally compressed by diagenetic action, can be referred to the right manus (Figs. 9A–9F). Its length is about 4/5 of the proximodistal length of manual phalanx II-1, indicating that the third digit was probably comparable in size to the second digit, albeit more slender (as it is often the case in theropods). Our referral to the right manus is corroborated by a striking mirror-image resemblance to the left III-1 of the holotype of *Ceratosaurus nasicornis* USNM 4735 (Carrano & Choiniere, 2016: fig.11) when observing, in particular, the position of the lateral flexor process in proximal view, the medial condyle margin continuing the concavity of the medial side of the bone, and the lateral condyle abruptly protruding from the lateral side of the shaft, in dorsal and ventral views. The seeming homology of the distal condyles to the left element figured by Carrano & Choiniere (2016) is due to some deformation occurred to our specimen.

In dorsal and palmar view, the proximal articulation shows a sigmoid margin as in the homologous phalanx of *Ceratosaurus nasicornis*, but differs from it in the dorsal extensor process, which is not bulbous (Carrano & Choiniere, 2016: fig.11). In proximal view, the proximal articulation appears to be made mostly by a single concavity, but at closer examination, towards the medial margin it shows continuity with a semilunate convexity. The small lateral flexor process appears hook-like, and well-distinct from the larger medial flexor process by a deep palmar flexor groove, which, unlike the groove of phalanx II-1, does not extend onto the posterior surface of the shaft, just like in the homologous elements of *Ceratosaurus* (C. Dal Sasso & S. Maganuco, 2014, personal observation on USNM 4735). In dorsal, ventral and distal views, the distal condyles are evidently constricted by a well-developed intercondylar sulcus that extends onto the dorsal face of the bone, and are asymmetrically inverted due to deformation: the medial condyle appears narrower and projecting distally into a sharp pointed edge; the lateral condyle is wider and inclined laterodorsally. The lateral collateral ligament pit is deeper than the medial.

The strict morphological affinity with *Ceratosaurus* rules out previous deductions, based on the (wrongly interpreted) single concavity of the proximal articulation of III-1, which regarded the Saltrio theropod as bearing a simple metacarpo-phalangeal joint on the third finger (Dal Sasso, 2003). In facts, in the articulated manus of *Ceratosaurus* the proximal surface of phalanx III-1 is concavo-convex just like in *Saltriovenator*, and such sigmoid surface matches perfectly with the two distal condyles of metacarpal III, which are mediolaterally asymmetrical like those of metacarpal II (C. Dal Sasso & S. Maganuco, 2014, personal observation on USNM 4735). Consequently we infer the presence of asymmetrical distal condyles in the third metacarpal of *Saltriovenator* (Fig. 10C), perhaps similar to the condition in *Berberosaurus* (Allain et al., 2007). Furthermore, even considering our bone as not deformed, the proximal articular fossa of phalanx III-1 in our specimen was likely larger than deep, as it is in *Ceratosaurus*. In absolute size, phalanx III-1 of *Dilophosaurus* is longer than in *Saltriovenator*, its shaft being more slender and elongate. Nevertheless, the edges of the proximal end are gently sigmoid and the proximal facet is similar in having a wide—although shallower—concavity (C. Dal Sasso, 2004, personal observation on UCMP 37302). Preserving complete hand bones, the type specimen of *Dilophosaurus* confirms that a seemingly single proximal concavity is not evidence of a single condyle in the proximal bone element articulating with it. Actually, below the concavity a robust medial flexor process protrudes proximally toward the midline; this process, when pronation and supination movement is simulated, acts as a guide in the intercondylar sulcus of metacarpal III, preventing rotation of the bones along their elongation axis. In the phalanx III-1 of *Allosaurus* (C. Dal Sasso, 2004, personal observation on MOR 693) the proximal articulation is a single concavity, the dorsal extensor process is almost absent and the medial flexor process is faint, making the bone margin only slightly convex in palmar view; in facts, the distal condyles of metacarpal III function as a unit because the intercondylar sulcus divides them only on the palmar side.

**Phalanx III-2.** The second phalanx of the third digit is perfectly preserved in three dimensions (Figs. 9G–9L). Its proximodistal length is about 9/10 that of manual phalanx III-1. By comparisons with *Allosaurus* (Madsen, 1976; C. Dal Sasso, 2004, personal observation on MOR 693), this element pertains to the right manus. In facts, in the proximal end the medial flexor process is more developed than the lateral and the dorsopalmar ridge runs closer to the medial margin of the bone, making the medial articular facet narrower than the lateral one. The palmar flexor groove and the lateral sulcus are short and shallow. In the distal articulation the condyles are almost subequal, less asymmetrical than in III-1, and the collateral ligament pits are almost absent.

Like phalanx III-1, III-2 of *Dilophosaurus* is longer and more slender than the one of *Saltriovenator*. A more important difference is that in *Dilophosaurus* the dorsal extensor process and the medial flexor process in lateral/medial view are equally developed, whereas in *Saltriovenator* the former is less robust and definitely shorter than the medial flexor process, much less developed either in proximal and in dorsopalmar direction, as it is in *Allosaurus* (C. Dal Sasso, 2004, personal observation on MOR 693). Moreover, in *Dilophosaurus* the proximal articular surface is a unique undivided and deep concavity (C. Dal Sasso, 2004, personal observation on UCMP 37302), whereas in *Allosaurus* a dorsopalmar ridge divides the proximal articulation in two symmetrical concave facets.

**Phalanx III-3.** This bone has been compressed mediolaterally, and the articulations have been smashed and deformed (Figs. 9M–9R), therefore it is not easy to locate its left or right position. However, the size of the bone, the elongation and slenderness of the shaft, and the curvature of the proximal articular surface are consistent with a right III-3, fitting well with the above described III-2; moreover, the medial flexor process appears more developed than the lateral one, which would confirm this positioning. The medial flexor process is also much more developed that the dorsal extensor process, which terminates in a pointed end, enlarged only in mediolateral direction. In this aspect, and in the relative proportions of the extensor and flexor processes, the penultimate phalanx of *Dilophosaurus* is similar to that of *Saltriovenator*, being only slightly more slender in the diaphysis (C. Dal Sasso, 2004, personal observation on UCMP 37302).

This element is longer than each of the more proximal phalanges of the third manual digit, but shorter than the sum of the two, when articulated. This condition, previously unknown in neoceratosaurians due to the frequent lack of distal phalanges in the fossil record or because of secondary simplification of the phalangeal formula (Burch & Carrano, 2012), is present in *Herrerasaurus*, *Dilophosaurus*, *Coelophysis* spp., *Allosaurus*, and the Oviraptorosauria, whereas in almost all other tetanurans (including basal birds) phalanx III-3 is longer than III-1 + III-2 (Rauhut, 2003).

**Phalanx III-4.** This ungual phalanx lacks the distal end and seems slightly compressed mediolaterally by diagenetic action, the articular fossae being very narrow, and the ridge dividing them appearing too sharp (Figs. 9S–9X). The lateral compression, the shape and the size of the proximal articulation fit well with the above described III-3, and are consistent with their anatomical connection, as well with proximity of deposition, under the same diagenetic events.

The original, regularly curved and pointed shape of the bone can be inferred from the preserved curvature of the dorsal margin. The dorsal extensor process is in continuity with it, as in the homologous unguals of *Dilophosaurus* (Welles, 1984) and *Allosaurus* (Madsen, 1976), and unlike the slightly lipped process of phalanx III-4 of *Sinraptor* (Currie & Zhao, 1993).

The flexor tubercle is well-distinct from the flattened shaft and it is placed distal to the proximal surface of the ungual. It protrudes ventrally with a dorsoventral diameter which is 1/4 the depth of the proximal articular surface, tubercle included, terminating with a rounded hemispherical end, only partially eroded, which is more reminiscent of *Eoabelisaurus* and *Sinraptor*, than *Allosaurus*; in *Dilophosaurus* (Welles, 1984; C. Dal Sasso, 2004, personal observation on UCMP 37302) the flexor tubercle is definitely less developed and terminates with a pointed end.

To the right side, the base of the flexor tubercle is marked by a ridge, and by a groove that arches dorsally becoming a simple unforked collateral furrow, as in *Eoabelisaurus* (Pol & Rauhut, 2012: Fig. 7), *Segisaurus* and *Coelophysis* (Carrano, Hutchinson & Sampson, 2005), and unlike *Limusaurus*, in which the manual unguals have two vascular grooves on each side (Xu et al., 2009).

**Indeterminate ungual fragment.** The claw tip in Figs. 13S–13X does not belong to the same ungual of Figs. 9S–9X, its transverse section being thicker, and D-shaped. Remarkably, the dorsal edge of the flat side is very sharp, which excludes it to be a pedal ungual. By parsimony, and by the fact that this fragment was embedded in block B together with all other elements of the right manus, we tentatively regard it as the tip of the right second manual digit (II-3).

### Hind limbs

#### Tarsus

**Figure 14:**
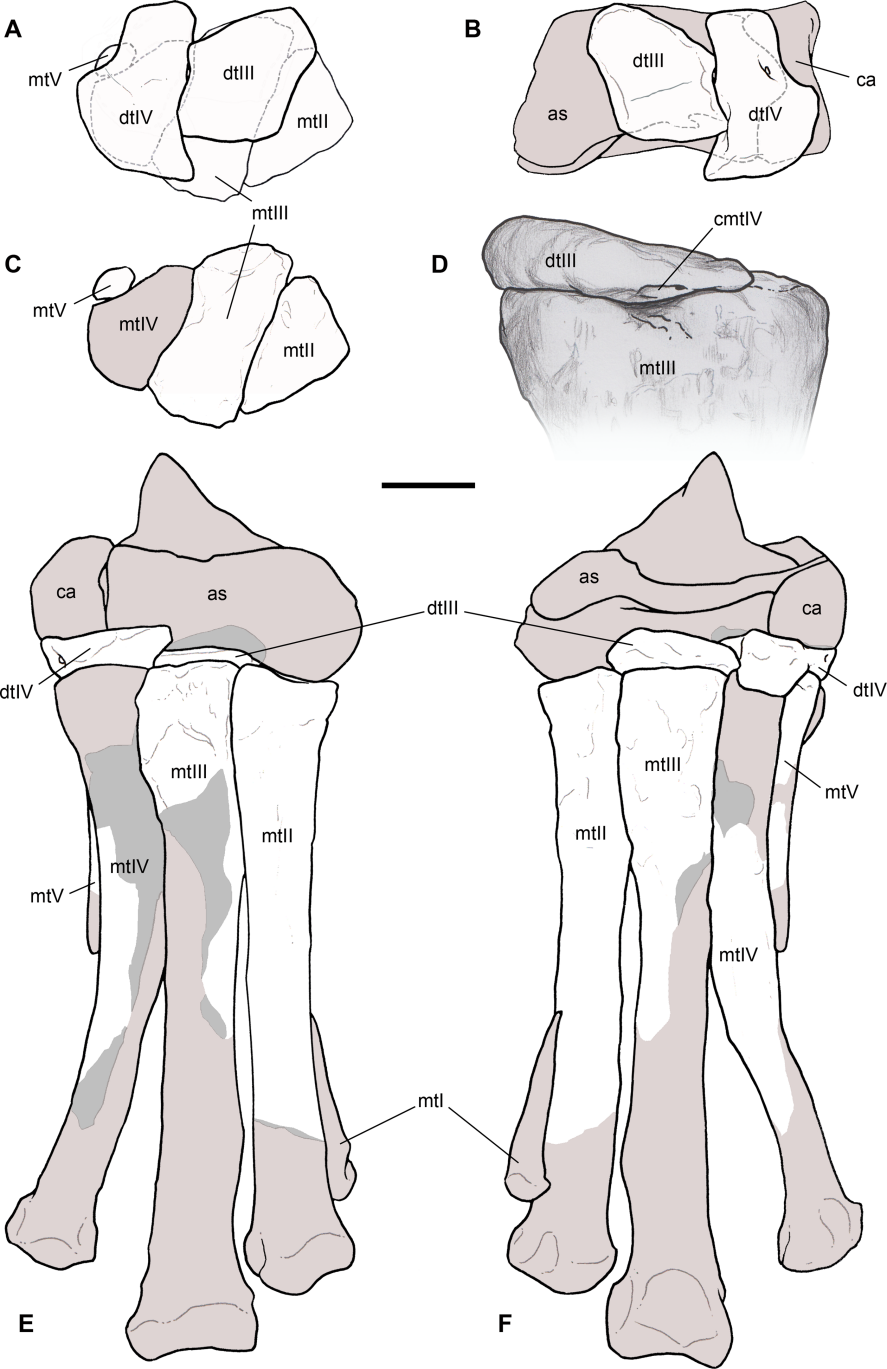
Reconstruction of the right ankle and foot of *Saltriovenator zanellai*. Distal tarsals superimposed and articulated to metatarsals in proximal view (A), and to astragalus and calcaneum in distal view (B); metatarsals II–V in proximal view (C); close-up of the perfect contact between distal tarsal III and metatarsal III in lateral view (D); tarsal and metatarsal elements fully reconstructed and articulated in cranial (E), and caudal (F) view. Abbreviations as in text, preserved elements in white (except in D), reconstructed bone in light gray, exposed inner bone in gray, hidden bone in dotted lines. Scale bar equals five cm. Scale bar equals five cm in (A)–(F), three cm in (D). Drawings by M. Auditore.

Distal tarsals are not often recovered with specimens because of delayed ossification and small size (Currie & Zhao, 1993). Remarkably, the right distal tarsals III and IV are among the few complete bones of *Saltriovenator*, and are beautifully preserved in three dimensions (Fig. 11), unfused to the metatarsals (Figs. 11 and 12). They articulate pretty well with each other, and with the proximal epiphyses of the preserved metatarsals. The whole complex belongs to the right ankle of *Saltriovenator*, which is reconstructed in Fig. 14.

Distal tarsals are well-preserved in the type specimen of *Dilophosaurus wetherilli*, but inadequately described and not figured in the formal description (Welles, 1984). Also *Dracoraptor* nicely preserves these bones (Martill et al., 2016: fig. 27), showing remarkable—but not discussed—affinity with the former taxon. The distal tarsals III and IV of *Dilophosaurus* are similar to the ones of *Saltriovenator*, both in general shape and relative size, but are about one fourth smaller; in addition, they are proportionately less developed in craniocaudal direction and the contacts with proximal tarsals and metatarsals appear less marked (C. Dal Sasso, 2004, personal observation on UCMP 37302). On the other hand, either in absolute and relative size, the distal tarsals of *Saltriovenator* are not significantly different from those of the medium-large Jurassic tetanurans *Allosaurus* and *Sinraptor.* In shape, distal tarsal III of *Saltriovenator* is somewhat similar to the compact distal tarsal III of *Allosaurus*, whereas distal tarsal IV is more angled than in these tetanuran theropods, showing a more complex morphology.

**Distal tarsal III.** This is a rhomboid and nearly flattened element, with two sides tapering cranially into a thinner side with a sharp margin, and one side which is definitely thicker and bulky, displaced caudally; consequently, in lateral and medial view this bone has a markedly cuneiform profile (Figs. 11A–11F).

The distal tarsal III of *Dilophosaurus* (C. Dal Sasso, 2004, personal observation on UCMP 37302) possesses a more inflated mediocaudal portion and a cranial edge which is thicker, round-finished and not at all sharpened, as in more basal coelophysoids (e.g., *Coelophysis rhodesiensis*—Raath, 1990). In *Allosaurus* (C. Dal Sasso, 2004, personal observation on MOR 693) this bone is more flattened along all sides, the caudal process is pointed rather than rounded, and the proximal face is straight. Nevertheless, similar to *Allosaurus*, the lateral side of distal tarsal III of *Saltriovenator* bears two pointed apices, divided by a concavity, which fit exactly the wavy medial side of distal tarsal IV; in particular, the craniolateral pointed articulation inserts in the large medial notch of distal tarsal IV (Figs. 11M and 11N).

In distal view, the distal tarsal III of *Saltriovenator* is crossed at mid-length by a ridged articulation for metatarsal III, directed mediolaterally, which is absent in *Allosaurus* (MOR 693) and reduced to a feeble bump in *Dilophosaurus* (UCMP 37302); the articulation for metatarsal II is an elongate depression that runs along the distomedial margin; two adjacent notches mark the contact with metatarsal IV along the distolateral side; the largest notch bears two pits at its bottom. All these reference points, coupled with the undeformed condition of this bone, allow to place perfectly distal tarsal III on top center of metatarsal III, with marginal mediolateral lapping over (Fig. 14A).

Besides the features listed above, the distal tarsal III of *Saltriovenator* differs from *Dilophosaurus* in the distal side: in *Dilophosaurus* it is depressed in the middle, in *Saltriovenator* it is convex and ridged in the middle; in *Dilophosaurus* the contact for metatarsal II is limited to the craniomedial corner, in *Saltriovenator* the slit for metatarsal II develops along the bone edge in caudal direction for most of its length (C. Dal Sasso, 2004, personal observation on UCMP 37302).

Interestingly, the convex cranial margin shown by the distal tarsal III of *Saltriovenator* has been recently described in *Powellvenator* (Ezcurra, 2017), contra the concave margin of *Coelophysis rhodesiensis* (Raath, 1977), and *Ceratosaurus* (USNM 4735), or the straight margin of *Dilophosaurus* (UCMP 37302) and *Dracoraptor* (NMW 2015.10G.1a/b). However, *Saltriovenator* differs from *Powellvenator* in other aspects: the concave contact for the astragalus is limited to a mid-medial portion of the proximal surface, rather than to the whole medial third, and there is not any caudal depressed surface, absent also in most other basal neotheropods (Ezcurra, 2017).

**Distal tarsal IV.** This is a blocky element, almost twice thicker than distal tarsal III, with a complex subtrapezoid shape and with proximodistal thickness varying considerably (Figs. 11G–11L): in medial view, the distal surface appears convex with a central concavity that likely matched the proximal articular surface of metatarsal IV (not preserved in our specimen); the proximal surface appears weakly sigmoid, with a caudal convexity and a cranial concavity for the astragalus, like in *Dilophosaurus* (UCMP 37302) and unlike the uniformly flattened aspect described in *Powellvenator* (Ezcurra, 2017). The latter taxon is also very different in having a strongly convex, almost subcircular cranial margin, whereas the tarsal IV of *Saltriovenator* has a wing-like margin expanded craniolaterally. An almost equally developed wing-like convex margin is present in *Dilophosaurus, Dracoraptor*, and other basal neotheropods (Ezcurra, 2017), whereas in *Ceratosaurus* the craniolateral expansion is reduced by squared margins (Madsen & Welles, 2000: fig. 10).

In proximal and distal views, emphasized by the wing-like expansion, the tarsal IV of *Saltriovenator* narrows into a subrectangular caudomedial portion and displays a broad, equally subrectangular caudolateral notch, which was considered an unambiguous apomorphy of the Ceratosauria (*sensu* Tykoski & Rowe, 2004, a clade including coelophysoids and neoceratosaurians: note that our preferred phylogeny does not support coelophysoids in Ceratosauria). In facts, in *Allosaurus* (MOR 693) the caudal portion of the bone is less constricted and the notch is not a squared corner but a gentle concavity, and in *Sinraptor* (Currie & Zhao, 1993) there is not even a concavity. In proximal view, the mid-lateral portion of the wing-like expansion bears a facet, delimited caudally by a transverse shallow groove. This is likely the articular contact with the calcaneum (Fig. 14B) and cannot be compared to the marked lateral spur seen in some maniraptorans. In distal view, the pointed craniomedial end of the wing-like expansion bears a flat triangular facet that, with the ankle and foot bones re-articulated, contacts the proximal craniolateral end of metatarsal III (Figs. 14A and 14E). A similar process is present in several basal neotheropods, such as the “Padian’s *Coelophysis*” (UCMP 129618), *Dracoraptor* (NMW 2015.10G.1a/b), *Dilophosaurus wetherilli* (UCMP 37302), but also in *Allosaurus* (C. Dal Sasso, 2004, personal observation on MOR 693); it is absent in *Powellvenator* (Ezcurra, 2017), *Coelophysis rhodesiensis* (Raath, 1977: fig. 19), *Segisaurus* (UCMP 32101), and “*Syntarsus*” *kayentakatae* (Rowe, 1989).

Four pits open on the bone edges, three on the caudolateral side and one on the medial. The latter, and the two largest lateral pits, open at the bottom of notches that are homologous to the three pitted notches of *Allosaurus* described and figured by Madsen (1976: fig. 25); the medial pitted notch is for the craniolateral pointed articulation of distal tarsal III; the large caudolateral pitted notch is the articulation for the proximal head of metatarsal V. This notch is associated to the subrectangular “ceratosaurian” bone margin described above. In facts, it is remarkably squared in *Ceratosaurus* (Madsen & Welles, 2000: fig. 10); slightly less excavated in *Dilophosaurus wetherilli* (UCMP 37302), *Dracoraptor* (NMW 2015.10G.1a/b), *Coelophysis rhodesiensis* (Raath, 1977, 1990), *Powellvenator* (Ezcurra, 2017), feeble in *Allosaurus* (Madsen, 1976) and almost absent in *Pandoravenator* (Rauhut & Pol, 2017) and *Sinraptor* (Currie & Zhao, 1993).

#### Metatarsus

The preserved metatarsus of *Saltriovenator* consists of: the proximal portion of the right metatarsal III; two incomplete bones formerly identified as a right fibula and an indeterminate long bone (Dal Sasso, 2001b, 2003), later respectively re-interpreted as the right metatarsals II and IV (Dal Sasso, 2004); and a fragmentary and much smaller bone, here tentatively interpreted as the proximal portion of the right metatarsal V. Besides morphological affinity and size consistency, this interpretation is strengthened by clearly matching articular contacts (metatarsals II–III), by the fact that most bones of the ankle have been recovered, and that all of them pertain to the right ankle (Fig. 14).

The tarsal and metatarsal elements of *Saltriovenator* show firm, sometimes interlocking mutual contacts, but no evidence of co-ossification nor fusion—including the proximal half of the shafts of the central metatarsals. In facts, they were found fully disarticulated in blocks A and B, some distance from one another (Figs. 2 and 3). Distal tarsals and metatarsals are unfused in known specimens of *Liliensternus*, *Dilophosaurus*, and *Elaphrosaurus* (Rauhut, 2003), as well as *Dracoraptor* (Martill et al., 2016).

Unlike *Allosaurus* (Madsen, 1976: fig. 25B) and like in the basal tetanuran *Pandoravenator* (Rauhut & Pol, 2017: fig. 8.5–6), in *Saltriovenator* metatarsal III and distal tarsal III fit at best by overlapping the latter until seeing alignment with the former along their lateral, rather than medial margins (Fig. 14A). This way, in distal tarsal III, the central ridge matches perfectly the proximal concavity of metatarsal III, the concave articulation with metatarsal IV (homologous to “1” in Currie & Zhao, 1993: fig 24) overhangs laterally metatarsal III enough to contact metatarsal IV (Fig. 14D), and the long contact for metatarsal II overhangs medially articulating with it (Figs. 14A and 14E). In *Ceratosaurus* (C. Dal Sasso & S. Maganuco, 2014, personal observation on USNM 4735), distal tarsal III is perfectly centered on metatarsal III, feebly overhanging equally the adjacent metatarsals.

In size, the tarsals of the subadult *Allosaurus* MOR 693 are identical to those of *Saltriovenator*, whereas the metatarsals are remarkably shorter (C. Dal Sasso, 2004, personal observation). Therefore, the ankle of *Saltriovenator* results similar in cross-section, but more elongate than that of *Allosaurus* and, in this aspect, almost as slender as in *Dilophosaurus* (Welles, 1984: fig. 36), with metatarsal IV more divergent. *Ceratosaurus* differs remarkably, at least in the best known specimen: Gilmore (1920) described the metatarsals *Ceratosaurus nasicornis* USNM 4735 as “firmly united to each other, […] evidently similar to that of a typical bird,” and “nearly a third shorter than the corresponding elements of a fully adult *Antrodemus* (=*Allosaurus*) specimen, though the relative lengths of the metatarsals to one another in the respective feet are very similar.” Following Rauhut (2003), this character is evidently related to individual variation and/or ontogeny (metatarsal fusion is also apparent in the largest—and likely fully adult—specimens of *Coelophysis*). In turn, *Limusaurus* likely shows a derived/specialized condition, having an almost straight metatarsal IV appressed against metatarsal III for its whole length, but metatarsal II unfused (Xu et al., 2009).

**Metatarsal II.** The former misintepretation of this bone was biased by its triangular epiphysis and by the presence of a depression below its flattened side, which was reminiscent of the fibular fossa of several theropods (Figs. 12A–12F). At closer look, that depression turned out to be an artifact of preservation, due to diagenetic crushing that caused a collapse of the hollow diaphysis and splitted in two a pre-existing semicircular macroboring. The distal epiphysis is missing. The interpretation of this bone as a metatarsal II is confirmed by a size criterion: as illustrated by Madsen (1976), its craniocaudal proximal diameter is almost the same of the distal tarsal IV, whereas, as a fibula, it would be expected to measure at least twice.

In proximal view the triangular proximal end shows a shallow central concavity oriented mediolaterally; the lateral flattened side is textured with thin vertical ridges and represents the contact with metatarsal III. The complete lateral overlap of metatarsal III over metatarsal II prevents any contact with metatarsal IV. The diaphysis is almost straight, gently bowed in craniomedial direction, and suboval in cross-section; distally it is broken transversely, showing a matrix-filled medullary cavity that occupies the 40–50% of the overall bone diameter.

The second metatarsal of *Saltriovenator* differs from that of *Dilophosaurus wetherilli* (C. Dal Sasso, 2004, personal observation on UCMP 37302), and most theropods, in the contour of the proximal articular surface, which is triangular rather than trapezoid (with flat medial side). In addition, in *Allosaurus* and other taxa metatarsal II is generally bulkier and possesses two wing-like processes that expand the proximal epiphysis cranially and caudally, increasing the area of articulation with metatarsal III. These processes lack in *Dilophosaurus*—as well as in *Saltriovenator—*and are moderately developed in *Sinraptor* (Currie & Zhao, 1993), *Ceratosaurus* (Gilmore, 1920) and *Eoabelisaurus* (Pol & Rauhut, 2012). A third difference is the very flat articular surface, instead of the central concavity seen in *Saltriovenator.*

**Metatarsal III.** Metatarsal III was embedded in block B, broken in two pieces and close to the right manual phalanges (Figs. 3H–3J), and now it can be easily recognized thanks to the wide contact areas visible on the sides of the intact proximal epiphysis (Figs. 12G–12L). Less than the proximal half of this element is preserved, the diaphysis being widely open along an extended oblique cut. Nevertheless, enough of the shaft is preserved to clearly show that it is well-ossified and lacks the arctometatarsalian condition. Slight vertical ridges and colored oxidation patterns that mirror the ones preserved on the lateral side of metatarsal II allow to re-articulate the two bones along their original contact surfaces.

In proximal view, metatarsal III has the shape of a right-angled trapezoid, with the oblique side placed caudally, and lateral and medial contacts for the adjacent metatarsals paralleling each other; the caudal side has almost the same width of the cranial side, and the latter is as similarly developed as that of metatarsal II, and aligned at the same level. Taken together, these conditions are present in *Ceratosaurus nasicornis,* although with a much higher robustness and degree of fusion (Gilmore, 1920; C. Dal Sasso & S. Maganuco, 2014, personal observation on USNM 4735), and likely in *Eoabelisaurus* (Pol & Rauhut, 2012: fig. 13). As in *Saltriovenator* and *Ceratosaurus*, the proximal ends of the metatarsals II and III have a subequal transverse width in most other neotheropods, including *Dracoraptor*, *Dilophosaurus, Liliensternus, Piatnitzkysaurus,* and *Powellvenator* (Ezcurra, 2017).

In *Allosaurus* (Madsen, 1976) and *Sinraptor* (Currie & Zhao, 1993), as well as in most Tetanurae, the proximal epiphysis of metatarsal III becomes biconcave and decreases in transverse width, sandwiched in between metatarsal II and IV. In *Saltriovenator* the craniocaudal diameter of the proximal end of the third metatarsal is only slightly longer than that of metatarsal II. The mediolateral diameter of the proximal epiphysis is similar to the mediolateral diameter of the shaft, and centrally concave is only the lateral side: unlike most tetanurans, and like in coelophysoids and ceratosaurians (e.g., *Majungasaurus*—Carrano, 2007), the metatarsal III of *Saltriovenator* is not really pinched and, in this respect, it is quite similar to that of *Dilophosaurus wetherilli*, in which the proximal epiphysis differs only in having a less pronounced lateral concavity for the metatarsal IV, and a rounded rather than flat cranial side (C. Dal Sasso, 2004, personal observation on UCMP 37302).

**Metatarsal IV.** The right metatarsal IV is more fragmentary, as it is represented by a portion of the diaphysis, open longitudinally to show a wide medullary cavity (Figs. 13A–13F). The bone shaft has the same anteroposterior diameter of metatarsal II but it is compressed mediolaterally and definitely curved, more than in *Ceratosaurus dentisulcatus* (Madsen & Welles, 2000: fig. 10) and *Dracoraptor* (Martill et al., 2016: fig. 27), and as it is in *Dilophosaurus wetherilli* (Welles, 1984: fig. 36), clearly showing that the diaphysis diverged laterodistally from the central longitudinal axis of the metatarsus.

In *Allosaurus fragilis* (Madsen, 1976; C. Dal Sasso, 2004, personal observation on MOR 693, C. Dal Sasso, personal observation, 2017 on AMNH FR 290 and 408), metatarsal IV is laterally bowed, with a curvature which is very much like the one observed in *Saltriovenator*, and varies in cross-section continuously: the proximal end is a scalene triangle, with the shortest side facing cranially; the diaphysis increases in diameter and becomes an equilateral triangle at midshaft, where the crest for metatarsal V begins. This confirms our interpretation: in our specimen, part of the flattened medial side that contacted metatarsal III is preserved proximally; below this enlarged portion, the cross-section of metatarsal IV is suboval, then (approximately at mid-shaft) the bone diameter increases, without becoming thicker than metatarsal II and III, and the cross-section becomes more compressed and drop-shaped, as a crest originates and runs along the caudolateral side of the bone. This crest is relatively short, not reaching the distal fourth of bone, and moderately tall. It can be interpreted as the scar for metatarsal V.

**Metatarsal V.** We refer to this bone two fragments that are not reconnectible but are remarkably compatible in size, cross-section, and texture (Figs. 13G–13L). Although fragmentary, this is clearly a long bone with a slender bar-like shaft ending with a convex proximal articular surface. Such surface is partially eroded but preserves a diameter and a pointed protuberance that fit very well, respectively, the fossa and the notch for the fifth metatarsal present on the right distal tarsal IV. Articulated this way, the shaft of this bone fragment also results properly oriented with its convex side facing externally (caudolaterally), the flat (and slightly twisted) side facing the shaft of metatarsal IV, and with a caudal ridge which is consistent with the “posterior ridge” of the metatarsal V described in *Dilophosaurus* by Welles (1984). In proximal view the proximal end of the bone is oval, as in *Powellvenator* (Ezcurra, 2017) but unlike most other taxa, including *Dilophosaurus*, in which it possesses a triangular shape.

If our interpretation of this element is correct, the metatarsal V of *Saltriovenator* appears almost as straight as in *Dilophosaurus*, but proportionately smaller and a bit shorter, if compared to the other metatarsals (Figs. 14E–14F). In *Segisaurus* and other coelophysoids, the distal end reaches down along one-third of the length of metatarsal IV (Carrano, Hutchinson & Sampson, 2005); in tetanuran theropods, such as *Allosaurus* (Madsen, 1976), *Sinraptor* (Currie & Zhao, 1993), *Acrocanthosaurus* (Currie & Carpenter, 2000), the fifth metatarsal is quite short, stout, and distally curved.

## Discussion

### Phylogenetic affinities of *Saltriovenator*

**Figure 15:**
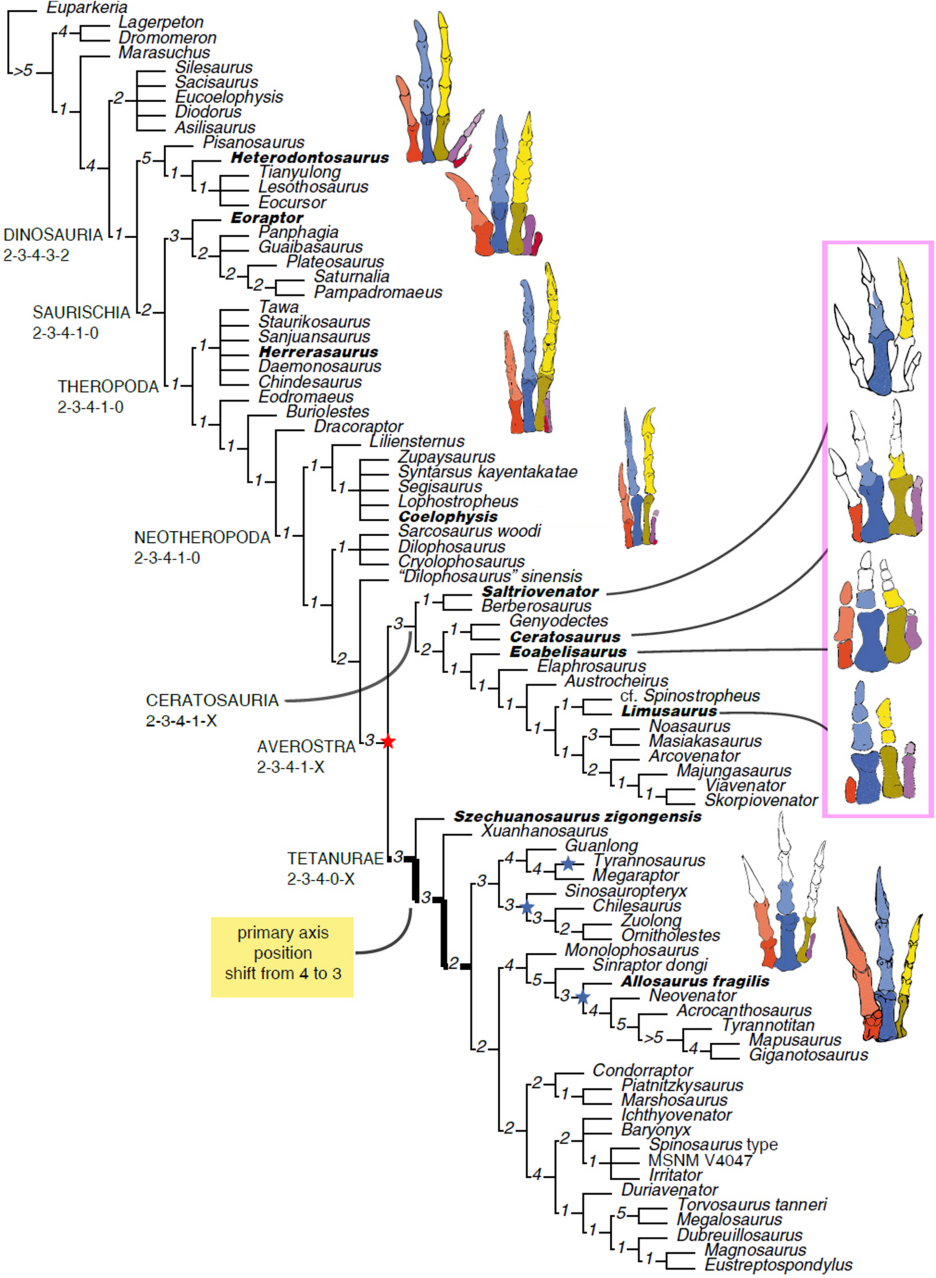
Phylogenetic affinities of *Saltriovenator* and evolution of the hand in Theropoda. Reduced strict consensus of the shortest trees found by the phylogenetic analysis after pruning of *Lewisuchus* and *Teleocrater*. Numbers at nodes indicate decay index. Inferred manual phalangeal formula for selected nodes indicated below clade names. Hands of representative members of the avian stem (bold names) in extensor view (*Herrerasaurus* in flexor view), medial side at left, missing elements in white based on ancestral states inferred at least inclusive node containing the taxon. Red star indicates loss of metacarpal V, blue stars indicate multiple independent losses of metacarpal IV among tetanurans. Drawings by A. Cau.

Our phylogenetic analysis (see also Data S1) found 624 shortest trees of 4,281 steps each (CI: 0.3069, RI: 0.5113). Exploration of the shortest topologies found shows that a large unresolved polytomy among the main dinosauriform lineages is due to the unstable position of *Lewisuchus* and *Teleocrater*, acting as “wildcards.” Once the two “wildcard” taxa have been pruned from the resulted topologies, the reduced strict consensus of the shortest trees found is well-resolved (Fig. 15) and places *Saltriovenator* as sister group of *Berberosaurus* along the basalmost ceratosaurian branch. Among theropods, the monophyly of Tetanurae, Averostra (the ceratosaurian-tetanuran clade excluding coelophysoids) and Neotheropoda is supported. Coelophysoid-grade theropods form a paraphyletic series leading to Averostra.

Unambiguous synapomorphies of the basal node of Ceratosauria (i.e., the least inclusive node containing *Saltriovenator* and other ceratosaurians) present in *Saltriovenator zanellai* are: a stout metacarpal II not longer than 5/2 its distal width; metacarpal II with distinctly shelf-like margin of collateral ligament fossa overhanging the fossae (particularly prominent along medial fossa); wide and prominent proximoventral processes in the manual phalanges, and reduction of the collateral ligament pits of the manual phalanges to shallow fossae not bordered by distinct lips. This node is also supported by the following synapomorphies present in *Berberosaurus* but not preserved in the Italian ceratosaurian: extensive pneumatization of the anterior presacral neural arches, and additional pneumatic foramen penetrating the posterior half of cervical centra.

A single unambiguous synapomorphy supports the sister-group relationship between *Berberosaurus* and *Saltriovenator*: the pronounced “lip-like” projection of the medial condyles of metacarpals II and III, which is directed proximopalmarly. This relatively weak support is explained by the limited anatomical overlap between the only two known specimens of *Berberosaurus* and *Saltriovenator*, restricted to the metacarpals.

*Saltriovenator* is excluded from Neoceratosauria (the ceratosaurid-abelisauroid clade) because it lacks: tooth crowns with flat or concave surfaces adjacent to the carinae; a relatively symmetrical and more equal development of the condyles of metacarpal II; a complete fusion of the tibiotarsus early in ontogeny; and a marked mediolateral expansion of the plantar margin of the proximal end of metatarsal III (“antarctometatarsalian” condition).

Enforcing *Saltriovenator* in Tetanurae (as originally suggested by Dal Sasso, 2001b), the shortest trees found are five steps longer than the unconstrained shortest trees: under this constraint, the Saltrio theropod is found in various alternative positions: as the basalmost tetanuran, as sister taxon of *Szechuanosaurus zigongensis*, as a coelurosaurian or within Megalosauridae. The latter two suboptimal scenarios are provisionally rejected also on stratigraphic ground, as they would imply several tetanuran branches currently unknown in the Early Jurassic (i.e., Megalosauridae, Spinosauridae, Allosauroidea, and Coelurosauria) to be extended back by at least 25 My (Carrano, Benson & Sampson, 2012).

Based on the phylogenetic framework of Wang et al. (2017), Delcourt (2018) proposed a radically alternative topology for Ceratosauria, where the noasaurid-elaphrosaurine grade taxa form the basalmost ceratosaurian clade which is sister group of Ceratosauridae + Abelisauridae. We re-run our phylogenetic analysis, enforcing the topology of Wang et al. (2017) and Delcourt (2018), setting *Saltriovenator* (not included in the analysis of Wang et al., 2017) as a floating taxon (i.e., its placement in the tree was not constrained by the enforced topology). The resulted shortest trees reconstructed under such constraint are 15 steps longer than our preferred scenario, and thus are rejected as not parsimonious interpretations of the data. Note that our data set is based on a larger character sample than the one of Wang et al. (2017)—1,781 characters vs 744—and includes several appendicular characters relevant in the placement of *Saltriovenator*. It is noteworthy that even under that alternative topological constraint, *Saltriovenator* resulted the basalmost ceratosaurian (i.e., sister group of Ceratosauroidea *sensu* Delcourt, 2018): thus, we conclude that our main evolutionary result (i.e., *Saltriovenator* representing the plesiomorphic condition of the ceratosaurian hand) is not biased by the data set used.

### Histology and ontogenetic status

The age and maturity of the holotypic specimen of *Saltriovenator zanellai* can be inferred by suture fusion and by analysis of histological thin-sections. In MSNM V3664, the scapulocoracoid is unfused and unsutured; the tarsal bones are fully ossified but not fused to metatarsals; the metatarsals do not show any sign of proximal co-ossification. These features might indicate that MSNM V3664 was a subadult, showing some but not all of the skeletal transformations that mark cessation of growth (Tykoski & Rowe, 2004). However, these and other common characters used to assess the ontogenetic stage of fossil tetrapods, such as surface texture of bones, and obliteration of the sutures in skulls and vertebrae, have been found to be ambiguous (Brochu, 1996; Werning, 2012; Bailleul et al., 2016). Moreover, skull and vertebrae are not preserved in our specimen. Therefore, histological analysis is the most reliable method for ontogenetic assessment and absolute estimation of the age of an individual (Chinsamy, 2005; Erickson et al., 2004; Erickson, 2005).

**Figure 16:**
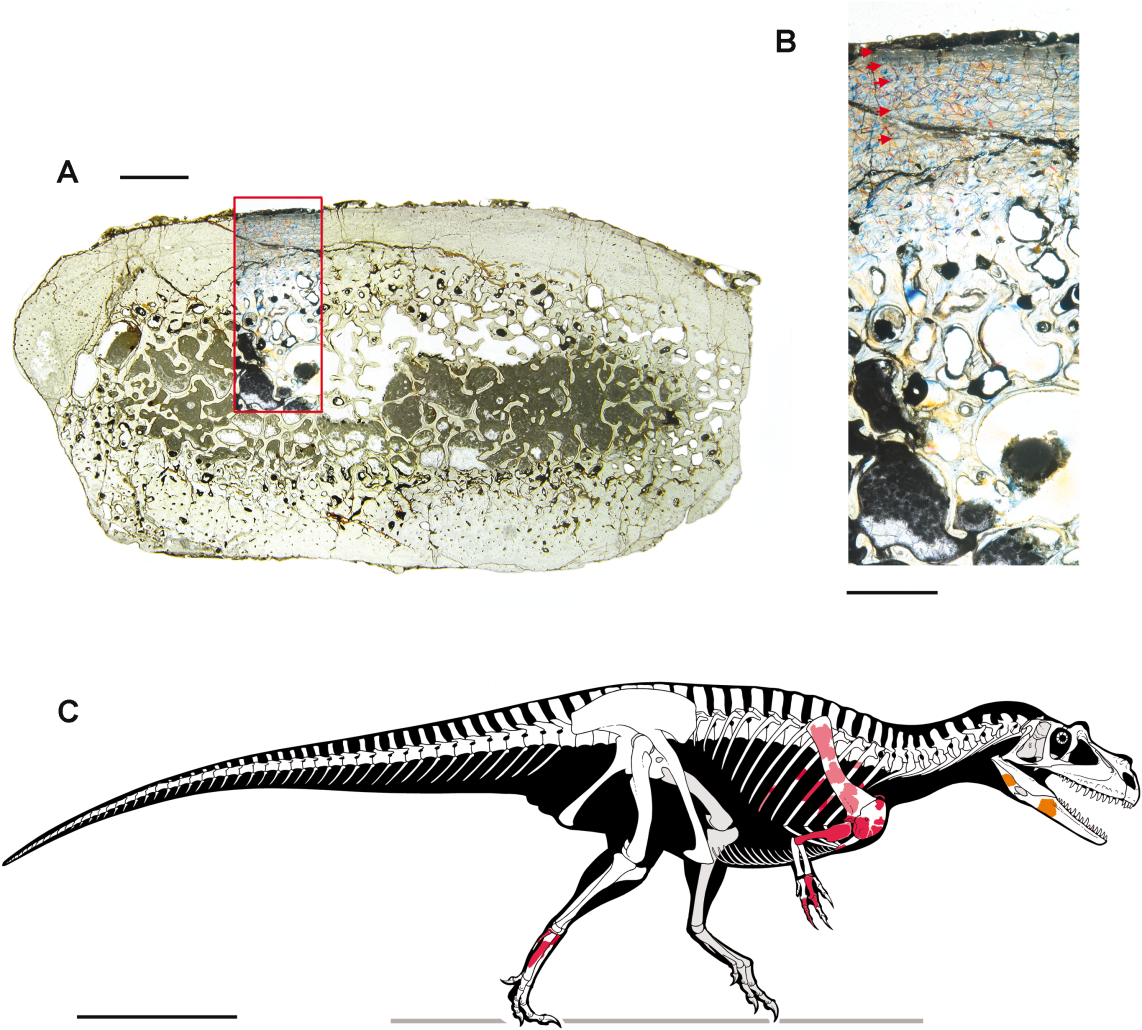
Bone microstructure (A–B) and skeletal reconstruction (C) of *Saltriovenator zanellai*. Mid-shaft thin section of a right dorsal rib. (A) Overall view; (B) close-up of the Haversian system in the compacta and of the microstructure of the outermost cortex showing the presence of an incipient EFS, interpreted on the basis of reduced vascular canals, presence of lamellar bone, and presence of closely spaced LAGs. The outer surface of the bone is at the top, red arrows point to the LAGs. Colors are emphasized due to photography under gypsum plate. (C) Skeletal reconstruction of *Saltriovenator zanellai* in right lateral view, realized using comparative anatomy and character state inference to predict a plausible range for size and proportions of the missing elements; known elements are mapped on the skeleton in different colors: right bones in red; counterlateral copies of the left bones in light red; bones from the medial side of the lower jaw in orange. Scale bars equal two mm in (A), one mm in (B), and one m in (C). Photos by M. Zilioli; drawing by M. Auditore.

We sampled the broken diaphysis of the left humerus, as well as a fragment of the shaft of a right dorsal rib (Figs. 16A and 16B). The type of microstructure, the density and type of vascular canals, the amount of remodelling, the number of LAGs, and the presence or absence of an EFS were the proxies used in this study to evaluate ontogenetic stage. Waskow & Mateus (2017) recently demonstrated that dorsal ribs record cyclical growth marks: sampling dorsal ribs 1–3 within the proximal third of the rib, but distal to the capitulum and tuberculum, recorded the most intact and complete history of LAGs. Our sample comes from the mid of the shaft and the LAGs record is therefore underestimated. Nonetheless, the data collected allowed to reliably infer the ontogenetic stage of the individual and if somatic and reproductive maturity were reached before death.

The sectioned bones do not differ in bone architecture to those of the other land-dwelling theropods (Erickson et al., 2004; Waskow & Mateus, 2017). In both sections, primary bone was observed in the outermost part of the compacta, towards the bone surface. It is woven fibrolamellar bone. In the rib section, most blood vessels are longitudinally oriented, and only a few ones are directed radially throughout the compacta. In the humerus the vascularization is more regularly organized, that is, radially directed in the inner portion and longitudinally directed towards the outermost cortex, suggesting a slowing down of the growth of the bone tissue and, as a consequence, of the animal. In the rib, the medulla is spongier in the middle, and the hollow medullary cavities are partly filled by matrix. In thin-sectioning the humerus, the medullary cavity was not sampled but the broken diaphysis shows that it is broad, open, and the passage between the medullary cavity and the compacta is abrupt.

Haversian systems are present and abundant in the inner cortex in both humerus and rib sections, and secondary osteons are visible closer to the outer surface. The latter elements are more abundant in the rib section. The amount of remodelling of the primary cortex clearly indicates that the animal was not juvenile. In the rib section, some LAGs are sometimes interrupted by secondary osteons but can be traced circumferentially. They are regularly spaced, except for the last (external, toward the outer cortex) LAGs, which are more closely spaced than the others. Due to the resorption of primary bone tissue caused by the expansion of the medullary cavity, only five LAGs are preserved in the cortical bone of the rib. As the rib section is not from the proximal third, this number of LAGs cannot be used to estimate its age at the time of death. Nonetheless, we performed a retrocalculation of the number of zones obscured from remodeling. For a consistent count of LAGs we followed Ibrahim et al. (2014) and employed three recognition criteria: the broadest zone, taken as representative of each missing band; the ultimate or penultimate zone; the mean interval between the three innermost zones. Using each criterion, respectively, we calculated 11, 27, and 19 missing LAGs, with a resulting mean of 19 years missing.

Thus, we estimate that the age of the holotype of *Saltriovenator zanellai* at the time of death was at least 24 years. The preservation of the humerus section does not allow to count LAGs. The combination of lamellar bone, reduced vascularization, and more closely spaced LAGs is here interpreted as an incipient EFS, more marked in the rib section. The presence of this incipient EFS and the remodelling observed in the compacta suggest that MSNM V3664 was a subadult approaching somatic maturity.

### Skeletal reconstruction and body size

Despite the incompleteness of our material, we attempted to reconstruct the skeleton of *Saltriovenator zanellai* n. gen. n. sp. using comparative anatomy and character state inference to predict a plausible range for the size and the proportions of the missing elements. Alternative methods, discussed in literature, were used, and their results compared.

#### Comparison with more complete specimens

**Figure 17:**
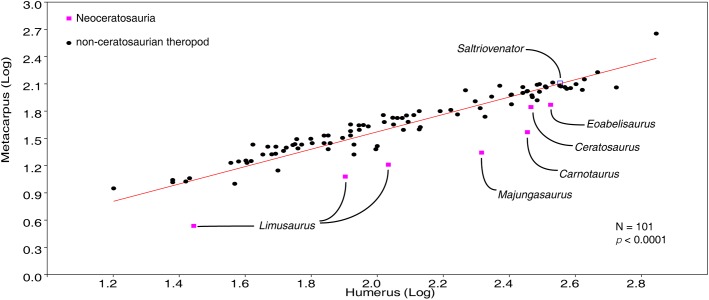
Plot of metacarpus vs humerus length in Theropoda. The metacarpus-humerus ratio in *Saltriovenator* perfectly fits the general theropod pattern, whereas more advanced ceratosaurians have the shortest metacarpi compared to the humerus among theropods. This suggests that the acquisition of a stout and robust metacarpus in Ceratosauria (present in *Saltriovenator*) preceded the relative size reduction of the metapodium (present in neoceratosaurs and extreme in abelisauroids). Data from Dececchi & Larsson (2011) and Wang et al. (2017). Diagram by A. Cau.

The scapula and humerus of *Saltriovenator zanellai* are, respectively, subequal and 10% longer than the corresponding elements of the articulated skeleton of a subadult *Allosaurus fragilis* (MOR 693), which is about 8 m long. In the hindlimb, the distal tarsals and the proximal end of metatarsals II and III of these two specimens are subequal in size. Compared to MOR 693, the forelimb elements in *Saltriovenator* are proportionally stockier and more robust (Figs. 16C and 17). We thus conservatively conclude that MSNM V3664 was at least seven to eight m long at the time of death.

Prior to the discovery of *Saltriovenator*, the coelophysoid-grade *Cryolophosaurus* was considered the largest Early Jurassic theropod (Smith et al., 2007). Although not enough comparable material with *Saltriovenator* is available (only the coracoids overlap), *Cryolophosaurus* is stated to be comparable in size to UMNH 5278, the largest specimen of *Ceratosaurus* (Smith et al., 2007; Madsen & Welles, 2000): based on comparable material with the latter, *Saltriovenator* is at least 25% larger.

#### Size estimation based on morphometric equations

Senter & Robins (2010) found that scapular length and humeral length correlate well (>90%) with the length of the hindlimb (HL, i.e., femur + tibia + metatarsal III), determining that the lengths of those forelimb elements have a good predictive value for the length of the hindlimb. As Senter & Robins (2010)’s formula is mostly based on tetanuran theropods and, among them, coelurosaurs, which usually have humeri considerably longer than in ceratosaurians, we used regression of the more conservative scapular length vs HL for *Saltriovenator*, finding a HL of 198 cm.

We also estimated the size of the femur of *Saltriovenator* using the morphometric data of Dececchi & Larsson (2011), focusing on the scapular length and humeral length of non-coelurosaurian theropods as proxies of femur length. In the sampled theropods, both measurements are robustly-correlated with femur length (scapular length: *p* < 0.0001, *n* = 18, *r*^2^ = 0.97; humeral length: *p* < 0.0001, *n* = 23, *r*^2^ = 0.92), and support a femur length for MSNM V3664 ranging between 822 and 887 mm, which is 5–13% longer than the femur of *Cryolophosaurus ellioti* holotype. Using the equation in Christiansen & Farina (2004), these values support a body mass range of 1,269–1,622 kg for the Italian theropod.

#### Body reconstruction

A skeletal reconstruction of *Ceratosaurus* (Madsen & Welles, 2000; C. Dal Sasso & S. Maganuco, 2014, personal observation on USNM 4735), the most complete and best known of the taxa closely related to *Saltriovenator zanellai*, was used as a blueprint, incorporating all the above mentioned data and all the known bones of our genus, to scale. Features and proportions of the unpreserved osteological elements represented in our skeletal reconstructions (Figs. 4Z and 16C) have been inferred based on character optimisation using the topology found in our phylogenetic analysis (i.e., assuming conservatively the missing elements of *Saltriovenator* from the ancestral state combination inferred at the basal node of Ceratosauria). A single median crest on the nasals and a distinct lacrimal crest are variably developed in basalmost averostrans known from cranial elements and belonging to both Ceratosauria (e.g., *Ceratosaurus*) and Tetanurae (e.g., *Monolophosaurus*, spinosaurids, proceratosaurids) (Carrano & Sampson, 2008; Carrano, Benson & Sampson, 2012). These features are optimized as averostran and ceratosaurian symplesiomorphies in our phylogenetic analysis. Accordingly, these ornamentations are depicted in our reconstruction of the Italian ceratosaurian, pending more complete material.

The outline of the flesh body in Fig. 16C was drawn on the basis of the usual distribution and attachment of the main muscular masses in theropods (Paul, 1988). The obtained whole reconstruction of *Saltriovenator* gives an approximate skull length of 80 cm, a total body length of about 730 cm and a hip height of about 220 cm. The body reconstruction supports a femur length of about 800–870 mm in the Saltrio theropod, using *Ceratosaurus nasicornis* as reference (Gilmore, 1920; Madsen & Welles, 2000), which suggests a body mass of about 1,160–1,524 kg (based on the equation of Christiansen & Farina, 2004).

#### Results

Given the fragmentary nature of the skeleton, and acknowledging the intrinsic uncertainties in any body reconstruction method, it is noteworthy that all estimations suggest the body mass of *Saltriovenator zanellai* close to or larger than 1,000 kg. Considering the age of the Saltrio Formation, which is early Sinemurian (199.3–197.5 mya), the holotype of *Saltriovenator zanellai* represents the most ancient large predatory dinosaur known from skeletal remains, and, in particular, the largest predatory dinosaur known from the Early Jurassic, surpassing in size the holotype of *Cryolophosaurus ellioti*, the latter with an estimated body length of 6.5 m and a mass estimated at 465 kg (Smith et al., 2007; note that using the equation of Christiansen & Farina, 2004, *Cryolophosaurus* mass is estimated at about 1,000 kg, again smaller than the mass range estimated with the same method for *Saltriovenator*) and rivalling with Late Jurassic specimens such as the holotype of *Sinraptor dongi* (Currie & Zhao, 1993) and *Allosaurus fragilis* MOR 693 (C. Dal Sasso, 2004, personal observation).

### Macroevolutionary implications of *Saltriovenator*

The holotypic skeleton of *Saltriovenator zanellai* belongs to a subadult individual approaching somatic maturity and shows a combination of coelophysoid-grade symplesiomorphies coupled with derived features shared with ceratosaurians and basal tetanurans (Figs. 4 and 16C). The strap-like scapular blade is comparable to basal ceratosaurians (e.g., *Ceratosaurus*; Madsen & Welles, 2000), differing from the broader fan-shape of coelophysoids (e.g., *Dilophosaurus*; Welles, 1984). The coracoid is similar to dilophosaurids (e.g., *Cryolophosaurus*; Smith et al., 2007) in the lack of a hooked caudoventral process and in the subtriangular prominent bicipital tubercle. The broad, V-shaped furcula bears a prominent hypocleideum. The humerus is robust and straight in lateral view, as in ceratosaurians and some basal tetanurans (Gao, 1993; Madsen & Welles, 2000), and the quadrangular hypertrophied deltopectoral crest is extended for half of bone length; in later ceratosaurians, the deltopectoral crest is variably reduced (Carrano & Sampson, 2008; Xu et al., 2009). The humeral condyles are stout but flattened distally as in most ceratosaurians (Carrano & Sampson, 2008). The partially-preserved hand combines a unique mosaic of tetanuran and ceratosaurian features. The second metacarpal is stout and robust, as in all ceratosaurians (Xu et al., 2009; Burch & Carrano, 2012; Carrano & Choiniere, 2016), whereas the high metacarpus/humerus ratio fits those of non-ceratosaurian theropods (Fig. 17). The distal end of the second metacarpal is abruptly expanded and twisted, bearing asymmetrically-developed condyles, as in *Berberosaurus* (Allain et al., 2007). The extensor ligament pit is very pronounced and bound proximally by a prominent lip, a combination of features recalling the longest metacarpal of basalmost tetanurans (Madsen, 1976; Dong, 1984; Gao, 1993; Senter & Robins, 2005): in most ceratosaurians, the pit is shallower and the lip poorly marked (Xu et al., 2009; Carrano & Choiniere, 2016). The diaphysis of the first phalanx of the second finger is very short and stout, as in ceratosaurians, and differing from the more slender and elongate phalanx seen in coelophysoid-grade theropods and basal tetanurans (Welles, 1984; Dong, 1984; Gao, 1993). As in many ceratosaurians and basal coelurosaurs, the proximal flexor processes are prominent (Novas, 1998; Carrano & Choiniere, 2016). Although the third metacarpal is lost, the third finger is completely preserved. It recalls the third finger of coelophysoid-grade theropods and most tetanurans in bearing four functional phalanges (Madsen, 1976; Welles, 1984), including an elongate penultimate phalanx and the ungual with a distinct articular surface and prominent flexor tubercle. This mix of plesiomorphic conditions is absent in other ceratosaurians, in which the third finger has variably-reduced phalangeal formulae (Xu et al., 2009), short distal phalanges and poorly-developed articular surfaces (Burch & Carrano, 2012). In the foot, the distal tarsals are not co-ossified neither fused to the metatarsals, a plesiomorphic condition that we do not consider biased by the relatively mature ontogenetic stage of the specimen. The fourth distal tarsal bears a distinct subrectangular notch for metatarsal V, as reported in some coelophysoids (Ezcurra, 2017). The proximal surface of the third metatarsal lacks both the mediolateral plantar expansion shared by coelophysoids and some ceratosaurians (Tykoski & Rowe, 2004), and the middle constriction present in basal tetanurans (Madsen, 1976). The fourth metatarsal is curved laterodistally, suggesting graviportal adaptations in the foot.

### The evolution of the hand in Ceratosauria and Tetanurae

Using the strict consensus of the shortest trees found, we infer the ancestral state of the manual phalangeal formula along the internodes of the avian stem lineage leading to Tetanurae (Fig. 15): Dinosauria, 2-3-4-3-2; Saurischia, 2-3-4-1-0 (loss of the fifth finger and reduction of the fourth finger to a single phalanx); Theropoda, 2-3-4-1-0 (retention of the saurischian ancestral condition); Neotheropoda, 2-3-4-1-0 (retention of the saurischian ancestral condition); Averostra, 2-3-4-1-X (loss of the fifth metacarpal); Tetanurae, 2-3-4-0-X (loss of the fourth finger); several lineages among Tetanurae (i.e., Allosauria, advanced tyrannosauroids, and maniraptoromorphs, including birds), 2-3-4-X-X (loss of the fourth metacarpal).

The analysis supports a step-wise lateral reduction trend along the avian stem, leading to a tetrametacarpal and tridactyl condition at the root of Tetanurae. The presence of a complete formula for finger I in *Eoabelisaurus*, for finger II in *Limusaurus*, and for finger III in *Saltriovenator* (and the retention of a phalanx in finger IV in *Limusaurus* and *Majungasaurus*) implies that the ancestral ceratosaurian formula must be 2-3-4-1-X. Relevant for the discussion on the homology of the manual elements in tetanurans, *Saltriovenator* demonstrates the morphological similarity between the second metacarpal of basalmost ceratosaurians and the longest metacarpal of basal tetanurans (e.g., compare *Saltriovenator* with *Acrocanthosaurus*, *Szechuanosaurus zigongensis*, and *Xuanhanosaurus*; Dong, 1984; Gao, 1993; Senter & Robins, 2005), thus filling the gap between the latter taxa and the other ceratosaurians (e.g., *Ceratosaurus*, Carrano & Choiniere, 2016). This result confirms that the longest metacarpal in tetanurans is homologous to metacarpal II of other theropods, and not to metacarpal III (*contra* Xu et al., 2009). Furthermore, the relatively gracile but fully-functional third finger of *Saltriovenator* (which bears three pre-ungual phalanges and a claw-like ungual phalanx) closely fits both the third finger of non-averostran theropods and the lateral finger of tetanurans, strongly supporting the homology between these elements (Fig. 15, yellow finger). In their review of the alternative homology patterns for the theropod hand, Xu et al. (2014a) discuss four alternative models (i.e., “frame-shift,” “lateral-shift,” “axis-shift,” and “central loss,” Xu et al., 2014a, fig. 4A): the morphological consistence of both metacarpal and phalangeal patterns between earliest ceratosaurians and tetanurans dismisses all the homology frameworks alternative to the axis-shift model, which results the most robust scenario for the evolution of the hand along the avian stem.

The phalangeal formula of *Limusaurus* (0-3-3-1-X) is thus markedly modified compared to the ancestral ceratosaurian (and averostran) formula and cannot be considered an ancestral stage for that of the three-fingered tetanurans, *contra* Xu et al. (2009).

Phylogenetic analysis places *Saltriovenator* as sister taxon of the other Early Jurassic averostran *Berberosaurus*: this lineage results the basalmost ceratosaurian branch and the oldest averostran radiation (Carrano & Sampson, 2008; Carrano, Benson & Sampson, 2012). The combination of pronounced extensor pits and hypetrophied dorsal lips, deeply gynglimoid articular surfaces and prominent extensor/flexor processes and fossae in the metacarpus and phalanges of *Saltriovenator* (Figs. 4, 8 and 9; Supplemental Movie 1) shows that the hand of the basalmost ceratosaurians was well-adapted to struggle and grasp and to resist digital dislocation during violent movements by manually ensnared prey (see below), as in allosauroids (Senter & Robins, 2005). This is interpreted as the symplesiomorphic condition of all averostran forelimbs. The shorter and atrophied hand in *Limusaurus* (and abelisaurids) is thus a secondary condition restricted to late-diverging ceratosaurians, and is not directly related to the evolution of the tridactyl hand of tetanurans. In this scenario, the basalmost tetanurans (Dong, 1984; Gao, 1993) bear metacarpals I–IV and a robust metacarpal II sharing an enlarged asymmetrical distal end with a deep extensor pit and a robust lip, as in *Saltriovenator.* A vestigial metacarpal IV is retained in several tetanuran lineages, supporting 2-3-4-0-X as the ancestral phalangeal formula for that clade (Bever, Gauthier & Wagner, 2011). The persistence of a robust metacarpal IV eventually bearing one phalanx, even in the late-diverging ceratosaurians with atrophied hands (Xu et al., 2009; Burch & Carrano, 2012) suggests that a developmental constraint kept the primary axis of the hand in digit 4 position in all non-tetanuran theropods (Vargas et al., 2008; Tamura et al., 2011; Xu et al., 2014a). On the contrary, the independent reduction to only three metacarpals in allosaurians, tyrannosauroids, and maniraptoromorphs may indicate that a medial shift of the primary axis (from digit position 4 to digit 3) had occurred along the basal branch of Tetanurae after the complete loss of the fourth finger (Bever, Gauthier & Wagner, 2011), which then allowed multiple losses of the vestigial metacarpal IV in tetanuran subclades (Fig. 15). Accordingly, the evolution of the tridactyl hand of birds is more parsimoniously explained by lateral loss of elements among non-tetanuran dinosaurs, followed by a single medial shift of the primary axis at tetanuran root once the fourth finger was lost, and the retention of the ancestral fingers I–II–III along the whole avian stem.

The sister-taxon relationships between *Saltriovenator* and the other Early Jurassic ceratosaurian, *Berberosaurus*, indicates a previously unknown early radiation of averostrans along the western margin of the Tethys. We estimate *Saltriovenator* length at ∼7.5 m, thus resulting the largest Early Jurassic theropod based on skeletal remains (Smith et al., 2007; Carrano & Sampson, 2008). With a body size comparable to many Middle and Late Jurassic tetanurans (Carrano, Benson & Sampson, 2012), *Saltriovenator* pre-dates the occurrence of large theropods (body mass approaching 1,000 kg) by over 25 My (Benson, 2010; Carrano, Benson & Sampson, 2012), reinforcing a scenario recently suggested on the basis of ichnological evidence (Sciscio et al., 2017). The radiation of larger and relatively stockier averostran theropods earlier than previously known may represent one of the factors that ignited the trend toward gigantism in Early Jurassic sauropods (Sander et al., 2011; McPhee et al., 2018).

### Remarks on the functional morphology of the manus

The phalangeal formula inferred for *Saltriovenator* gives solid ground to the prediction (Carrano & Choiniere, 2016) that the basal ceratosaurian manus retained a morphology similar to that of *Dilophosaurus*, with reduction in length of the hand initially occurring through shortening of each phalanx, while a full complement of phalanges (including unguals) was still present. As stated above, this also argues against identifying the basal node of Averostra as the phylogenetic location for a major shift in digit identity or homology (*contra* Xu et al., 2009; Bever, Gauthier & Wagner, 2011). The second metacarpal of *Saltriovenator* shows—and somewhat emphasizes—functionally-related similarities with those of *Ceratosaurus* and *Dilophosaurus*: the distal end exhibits well-developed articular surfaces of comparably wide extent, showing that the proximal phalanx was capable of similar ranges of flexion and extension; the same occurs in the deeply gynglimoid articulations of the preserved distal phalanges.

According to Welles (1984), the pit on metacarpal II of *Dilophosaurus* allowed a 90° hyperextension of the proximal phalanx. The pit-and-lip complex of *Saltriovenator* allowed a 65° hyperextension, and contemporarily a firm hold-in-place (Supplemental Movie 1). In other words, similar to the basal tetanuran *Acrocanthosaurus*, *Saltriovenator* was adapted to struggle and grasp and to resist digital dislocation during violent movements by manually ensnared prey (Senter & Robins, 2005). Although a relatively shorter manus may imply a reduction in the size of objects that could have been grasped, the proportionally stouter manual elements in *Saltriovenator* may represent an adaptation to sustain during predation mechanical loads more intense than those sustained by the more gracile-limbed coelophysoid-grade theropods.

A second important similarity with some medium and large-bodied neotheropods is the asymmetry and rotation of the metacarpal condyles. In *Dilophosaurus* the rotation of 30° with respect to the vertical, which is comparable to that of *Saltriovenator* (Supplemental Movie 1), causes the proximal phalanx to project about 15° medially at full extension (Welles, 1984). In *Ceratosaurus*, this flexion would turn the three first digits of the hand medially, instead of extending straightforward (Gilmore, 1920). This feature is widespread in different neotheropod taxa, and may represent a symplesiomorphy of this predatory clade, evidently giving functional advantage. For example, in *Acrocanthosaurus,* the asymmetrical joint allows the first phalanx of the second digit to hyperextend as much as it flexes (about 40°), and turns the digit so that the tip of the claw would have rotated medially during flexion and laterally during hyperextension (Currie & Carpenter, 2000). A comparable adaptation is also reported in megaraptorids (see White et al., 2015).

## Conclusions

*Saltriovenator zanellai* gen. et sp. nov. is a new theropod dinosaur from the Lower Jurassic of Northern Italy. It represents the third named species of non-avian dinosaur from Italy, the first of Jurassic age. *Saltriovenator* shows a combination of ceratosaurian and tetanuran features, supporting close relationships between the two averostran lineages with the exclusion of coelophysoid-grade theropods. It also represents the first skeletal material supporting the occurrence of large and robustly-built predatory dinosaurs just at the aftermath of the Triassic–Jurassic boundary extinction events. Accordingly, the Italian ceratosaurian fills a stratigraphic and ecomorphological gap between the relatively more gracile coelophysoid-grade neotheropods (known from the Late Triassic to the Early Jurassic) and the large-bodied averostrans that occupied the majority of the apex predatory roles in the terrestrial ecosystems between the Middle Jurassic and the end of the Cretaceous.

The phylogenetic framework integrated with the new combination of features present in *Saltriovenator* dismisses the “II–III–IV homology pattern” in the interpretation of the tetanuran (and avian) hand, and suggests a complex process leading to the atrophied forelimb of later ceratosaurians. The evolution of a stocky and robust hand occurred in ceratosaurians before the relative shortening and the loss of predatory function: such a step-wise scenario raises intriguing perspectives on what adaptive and developmental factors led from a “*Saltriovenator*-like” condition to the aberrant condition present in *Limusaurus* and abelisaurids.

## Supplemental Information

10.7717/peerj.5976/supp-1 Supplemental Information 1 Animation of finger range of motion in Saltriovenator. ‘In the second finger of the Italian ceratosaurian, the strongly asymmetrical and deeply gynglimoid metacarpo-phalangeal joint allows the first phalanx to hyperextend as much as it flexes. Note that the tip of the claw would have rotated medially during flexion and laterally during hyperextension. In addition, the pit- and-lip complex of *Saltriovenator* ensured the base of the finger a firm hold-in-place at full extension. (Photogrammetry and animation by G. Bindellini)’.

10.7717/peerj.5976/supp-2 Supplemental Information 2 *Data matrix*. ‘Data matrix in Nexus format (raw data) including character scores and character statements of the phylogenetic analysis used in this study’.

## Anatomical Abbreviations

I–IV: first to fourth manual digit
1–4: first to fourth manual phalanx
ac: acromion
an: angular
ar: articular
as: astragalus
asc: concave contact with astragalus
b: bump
bt: bicipital tubercle
ca: calcaneum
cac: contact surface for calcaneum
cap: humeral head
car: carpal
cas: convex articular surface
cbf: fossa for the *M. coracobrachialis*
cf: collateral furrow
clp: collateral ligament pit
co: coracoid
cogl: coracoid glenoid
cr: cervical rib
csa: contact surface for surangular
d: dentary
dca: distal carina
de: tooth denticles
dep: dorsal extensor process
df: distal fossa
def: dorsal extensor fossa
dg: dorsal groove
dl: distal lamina of the deltopectoral crest
dlg: dorsolateral groove
dpc: deltopectoral crest
dpr: dorsopalmar ridge
dr: dorsal rib
dtIII: distal tarsal III
dtIIIc: concave contact with distal tarsal III
dtIIIn: notch for distal tarsal III
dtIV: distal tarsal IV
dtIVc: pointed contact with distal tarsal IV
ec: ectocondyle
ect: ectepicondylar crest
ell: extensor ligament lip
elp: extensor ligament pit
en: entocondyle
ent: entepicondylar crest
epc: epicleideum
exc: extensor crest
flf: flexor ligament fossa
ft: flexor tubercle
fu: furcula
g: groove
gl: glenoid fossa
hss: histological sampling section
hu: humerus
hyc: hypocleideum
ib: infraglenoid buttress
ics: intercondylar sulcus
idsl: interdenticular slit
idsp: interdenticular space
k: keel
l: left
la: labial side
lco: lateral condyle
lds: scar for *M. latissimus dorsi*
li: lingual side
lfp: lateral flexor process
llp: lip-like projection
ls: lateral sulcus
lt: lateral tuberosity
mbr: macroboring
mcI–IV: metacarpal I–IV
mcIc: contact surface for metacarpal I
mcIIIc: contact surface for metacarpal III
mca: mesial carina
mco: medial condyle
mfp: medial flexor process
mtI–V: metatarsal I–V
mtIIc: contact surface for metatatarsal II
mtIIIc: contact with metatatarsal III
mtIIIrc: ridged contact with metatarsal III
mtIVc: contact with metatarsal IV
mtVc: contact crest with distal half of metatarsal V
mtVn: notch for metatarsal V
nfgl: nutrient foramen of the glenoid
no: notch
p: pit
pff: proximal palmar flexor fossa
pfg: palmar flexor groove
pra: prearticular
q: quadrate
r: right
ri: ridge
sa: surangular
sc: scapula
scc: contact surface for scapula
scgl: scapular glenoid
scof: fossa for the *M. supracoracoideus*
sp: splenial
snf: supracoracoid nerve foramen
st: sternal plate
t: tab
vlg: ventrolateral groove.

## Institutional Abbreviations

AMNH: American Museum of Natural History, New York
FMNH: Field Museum of Natural History, Chicago
MPG: Museo Paleontológico de Galve, Teruel
MOR: Museum of the Rockies, Bozeman
MNHN: Muséum National d’Histoire Naturelle, Paris
MSNM: Museo di Storia Naturale di Milano, Milano
MWC: Museum of Western Colorado, Grand Junction, Colorado
NMW: National Museum of Wales, Cardiff
UCMP: University of California Museum of Paleontology, Berkeley
UMNH: Utah Museum of Natural History, Salt Lake City
USNM: National Museum of Natural History, Washington DC
UUVP: University of Utah Vertebrate Paleontology, Salt Lake City.

## Additional Information and Declarations

### Data Availability

The following information was supplied regarding data availability: The phylogenetic data matrix is available as a Supplemental File. The fossil material described in the manuscript is stored in the Paleontological Collection of the Museo di Storia Naturale di Milano (MSNM), with the following accession numbers: MSNMV3659 (tooth); MSNMV3664 (disarticulated skeleton).

### New Species Registration

The following information was supplied regarding the registration of a newly described species: Publication LSID: urn:lsid:zoobank.org:pub:DBF732EB-6D24-48D2- 8E5E-1C83EB380FD2. Genus name Saltriovenator Dal Sasso, Maganuco & Cau LSID: urn:lsid:zoobank.org:act:8C9F3B56-F622-4C39-8E8B-C2E890811E74. Species name, Saltriovenator zanellai LSID: urn:lsid:zoobank.org:act:BDD366A7-6A9D-4A32-9841-F7273D8CA00B.
